# The effect of nutrition-specific and nutrition-sensitive interventions on the double burden of malnutrition in low-income and middle-income countries: a systematic review

**DOI:** 10.1016/S2214-109X(23)00562-4

**Published:** 2024-01-29

**Authors:** Nora A Escher, Giovanna C Andrade, Suparna Ghosh-Jerath, Christopher Millett, Paraskevi Seferidi

**Affiliations:** Public Health Policy Evaluation Unit, School of Public Health, Imperial College London, London, UK; Centre for Epidemiological Studies in Health and Nutrition, University of São Paulo, São Paulo, Brazil; The George Institute for Global Health, New Delhi, India; Public Health Policy Evaluation Unit, School of Public Health, Imperial College London, London, UK; NOVA National School of Public Health, Public Health Research Centre, Comprehensive Health Research Center, NOVA University Lisbon, Lisbon, Portugal; Instituto de Estudos para Políticas de Saúde, São Paulo, Brazil; Public Health Policy Evaluation Unit, School of Public Health, Imperial College London, London, UK

## Abstract

**Background:**

Low-income and middle-income countries (LMICs) experiencing nutrition transition face an increasing double burden of malnutrition (DBM). WHO has urged the identification of risks and opportunities in nutrition interventions to mitigate the DBM, but robust evidence is missing. This review summarises the effect of nutrition-specific and nutrition-sensitive interventions on undernutrition and overnutrition in LMICs.

**Methods:**

We searched four major databases and grey literature for publications in English, French, Portuguese, and Spanish from Jan 1, 2000, to Aug 14, 2023. Eligible studies evaluated nutrition-specific or nutrition-sensitive interventions on both undernutrition and overnutrition, employing robust study designs (individually randomised, cluster randomised, and non-randomised trials; interrupted time series; controlled before–after; and prospective cohort studies). Studies were synthesised narratively, and classified as DBM-beneficial, potentially DBM-beneficial, DBM-neutral, potentially DBM-harmful, and DBM-harmful, using vote counting. This review is registered with PROSPERO (CRD42022320131).

**Findings:**

We identified 26 studies evaluating 20 nutrition-specific (maternal and child health [MCH] and school-based programmes) and six nutrition-sensitive (conditional cash transfers and other social policies) interventions. Seven of eight MCH interventions providing food-based or nutritional supplements indicated possible DBM-harmful effects, associated with increased maternal or child overweight. Most school-based programmes and MCH interventions that target behavioural change were considered potentially DBM-beneficial. Two studies of conditional cash transfers suggested DBM-beneficial effects in children, whereas one indicated potentially harmful effects on maternal overweight. A study on a family planning service and one on an education reform revealed possible long-term harmful effects on obesity.

**Interpretation:**

There is considerable scope to repurpose existing nutrition interventions to reduce the growing burden of the DBM in LMICs. In settings undergoing rapid nutrition transition, specific policy attention is required to ensure that food-based or supplement-based MCH programmes do not unintentionally increase maternal or child overweight. Consistent reporting of undernutrition and overnutrition outcomes in all nutrition interventions is essential to expand the evidence base to identify and promote interventions maximising benefits and minimising harms on the DBM.

**Funding:**

President’s Scholarship (Imperial College London) and National Institute for Health and Care Research.

## Introduction

The proclamation of the UN’s Decade of Action on Nutrition. includes a call to scale up global efforts to eliminate all forms of malnutrition by 2030. In low-income and middle-income countries (LMICs), the focus has long been on addressing undernutrition. However, rapid nutrition transition driven by economic growth, urbanisation, globalisation, and technological change has led to a growth of the ultra-processed food market and a shift away from traditional healthy diets.^1–5^ This shift has sowed the seeds for the coexistence of undernutrition with overweight, obesity, and diet-related non-communicable diseases (NCDs) within the same individuals, households, or countries—a situation defined as the double burden of malnutrition (DBM).^6^

More than one in three LMICs are affected by the DBM at the country level, with high prevalence of undernutrition (stunting >30%, wasting >15%, or female thinness >20%) and overweight (>20%), which is especially prominent in countries in the Asia-Pacific region and sub-Saharan Africa.^7–9^ In Latin America, child stunting decreased by more than half from 25% in 1990 to 11% in 2015, whereas child and adolescent overweight rates rose from 22% in 2000 to 31% in 2016, marked by large regional disparities.^10,11^ The World Bank warns that without appropriate interventions, the risk of NCDs linked to the DBM in LMICs could surpass the burden of NCDs in developed countries in the future.^12^

WHO has called for double-duty actions to tackle the DBM. Double-duty actions refer to nutrition interventions that address undernutrition and overnutrition simultaneously by leveraging shared drivers, such as food environments, socioeconomic factors, early childhood nutrition, and diet quality.^13,14^ Nutrition interventions, including nutrition-specific and nutrition-sensitive interventions, can directly or indirectly affect malnutrition. Nutrition-specific interventions target immediate causes of malnutrition, such as food intake or childcare practices, whereas nutrition-sensitive interventions focus on underlying factors, such as resource availability and accessibility.^15,16^

There is an urgent policy need to understand the effect of nutrition interventions on all forms of malnutrition.^14^ Previous reviews have focused on the implementation and effect of actions specifically targeting the DBM—ie, including double-sided objectives.^17,18^ These reviews concluded that implementation remains scarce and the effect on the DBM unclear due to heterogeneity in measured outcomes.^17,18^ However, WHO has recognised that double-duty actions are not limited to newly designed interventions;^14^ they can also be retrofitted from existing single-sided actions, ensuring that they do not have unintended harmful consequences on other forms of malnutrition.^14^ Therefore, this review summarises the effects of both nutrition-specific and nutrition-sensitive interventions, irrespective of their objective, on undernutrition and overnutrition in LMICs, to identify risks and opportunities for double-duty actions.

## Methods

### Search strategy and selection criteria

This systematic review adhered to the PRISMA guidelines and we registered our review protocol with PROSPERO (CRD42022320131).^19,20^ We searched MEDLINE, Embase, Global Health, Web of Science, and grey literature using Virtual Health Library, Global Index Medicus, the World Bank Open Knowledge Repository, the Food and Agriculture Organization, the World Food Programme, the Pan American Health Organization, and the Instituto Brasileiro de Defesa do Consumidor (appendix 4 pp 13–17). We used a search strategy developed with an information specialist and synonyms for undernutrition, overnutrition, and nutrition interventions, informed by a conceptual framework (appendix 4 p 4).^21^ Searches were limited to articles in English, French, Portuguese, and Spanish published between Jan 1, 2000, and Aug 14, 2023. Articles were uploaded to Covidence, which removed duplicates. Title and abstract and full-text screening were independently conducted by NAE and GCA. Disagreements were resolved by PS. When full texts were not available, we contacted corresponding authors. We hand-searched citations of included studies for additional relevant studies.

We included studies reporting the effect of nutrition-specific or nutrition-sensitive interventions on at least one indicator of overnutrition and one indicator of undernutrition, not necessarily in the same target population. Indicators of overnutrition included overweight, obesity, macrosomia, and large-for-gestational age. We verified whether the studies adhered to the definitions recommended by WHO. Indicators of undernutrition included stunting, underweight, wasting, thinness, anaemia, low birthweight, small-for-gestational age, height-for-age Z score, and haemoglobin concentrations (cutoff values for these categories can be found in appendix 4 [p 2]). Studies reporting only continuous anthropometric outcomes where overnutrition and undernutrition cannot be defined, such as a change in BMI, were only included if effect estimates were stratified by nutritional status at baseline (eg, change in BMI stratified by underweight, healthy weight, and overweight at baseline). Eligible studies were those from LMICs targeting the general population of all ages as well as pregnant or lactating people. We excluded studies only including participants with diagnosed illnesses (eg, diabetes and HIV).

We defined inclusion criteria for study design in line with the recommendations by the Cochrane Effective Practice and Organization of Care (EPOC) group;^22^ this includes all individually randomised controlled trials (RCTs), interrupted time series, cluster RCTs, non-randomised trials, and controlled before–after studies with more than one intervention and control site. In addition, we included prospective cohort studies due to their documented suitability to distinguish cause and effect,^23,24^ and cluster RCTs, non-randomised studies, and controlled before–after studies with only one intervention or control site, as a narrative synthesis is used in this review. We excluded non-comparative, cross-sectional, and uncontrolled before–after studies.^22^ Contrary to the review protocol, case-control and retrospective cohort studies were excluded due to limitations in establishing causal relationships (appendix 4 p 2).

### Data analysis

NAE conducted data extraction and quality assessment in Covidence using a template (appendix 4 pp 18–21), and GCA verified the extraction of all data and independently repeated the extraction for effect estimates and quality assessment. In cases of discrepancies, PS was consulted. For studies reporting multiple effect estimates, only effects from unadjusted and most adjusted models and those from the last follow-up were extracted. For quality assessment, we used the Effective Public Health Practice Project (EPHPP) Quality assessment for Quantitative Studies, considering selection bias, study design, confounding, data collection, withdrawals and drop-outs, intervention integrity, and analyses (appendix 4 pp 20–21).^25^

Studies were grouped into nutrition-specific and nutrition-sensitive interventions based on a conceptual framework (appendix 4 p 4).^21^ Nutrition-specific interventions target immediate causes of malnutrition and included maternal and child health (MCH) interventions, delivered to pregnant or lactating people, newborns, or children up to school age; and school-based interventions, delivered to children in schools. Within both categories, interventions either involved altering the food environment or implementing behavioural strategies. Nutrition-sensitive interventions addressed underlying determinants of nutrition and included conditional cash transfers (CCTs) and other social policies.

Given the heterogeneity of included interventions and outcomes, we used narrative synthesis^26^ and vote counting,^27^ following guidance from the Cochrane Handbook for summarising findings when meta-analysis was not feasible.^28^ First, we grouped reported outcomes into two domains of undernutrition and overnutrition. We then determined each intervention’s effect on both outcome domains, counting outcomes based on the direction of effect, regardless of magnitude or statistical significance.^27^ Interventions were assigned an overall effect direction if 70% or more of the effects for different outcomes within one domain pointed in the same direction, and there were no changes or conflicting findings were assigned otherwise.^27^ Finally, we reported the number of total and statistically significant outcomes (p<0·05) contributing to the overall effect direction of each domain. Based on the effect direction and statistical significance, we assigned each study’s effect on the DBM: DBM-beneficial, defined as a decrease in both domains with significant effect estimates, potentially DBM-beneficial, defined as a decrease in both domains without significant effect estimates; DBM-neutral, defined as no changes or conflicting findings for both domains or an overall decrease in only one domain; potentially DBM-harmful, defined as an increase in one or both domains without significant effect estimates; and DBM-harmful, defined as an increase in one or both domains with significant effect estimates.

We performed a sign test (binominal probability test) using the binom_test function in RStudio (version 2023.06) to calculate the proportion of beneficial and harmful effects. Under the null hypothesis, the proportion of decreased (*vs* no effect and increased) and increased (*vs* no effect and decreased) is 0·5, which would be the expected outcome under random chance.^28^ For each type of intervention, we performed separate sign tests for undernutrition and overnutrition outcomes and presented the proportion of decreased and increased outcomes with the respective 95% CIs and p values.

We presented effect estimates for categorical and continuous outcomes, specifying the type of effect measure used (appendix 4 pp 6–11). We used 95% CI as a measure of uncertainty, estimating it using SE or p values when needed.^29^ If data were missing or implausible, we contacted the corresponding author. When a corresponding author did not respond and the data provided in the paper did not allow the calculation of effect estimates or 95% CIs, we displayed the data as available in the paper (appendix 4 pp 6–11).

For vote counting, we considered categorical outcomes, unless a study only reported changes in continuous outcomes stratified by nutritional status at baseline. Only overall effects (not subgroup-specific) were considered. Three studies presented stratified results without providing an overall estimate.^30–32^ If effects consistently pointed in the same direction for all subgroups and both outcome domains, only that effect direction was presented. If effect directions were different between subgroups, we assigned DBM implications for each subgroup separately.

### Role of the funding source

The funders of the study had no role in study design, data collection, data analysis, data interpretation, or writing of the report.

## Results

**Figure 1:**
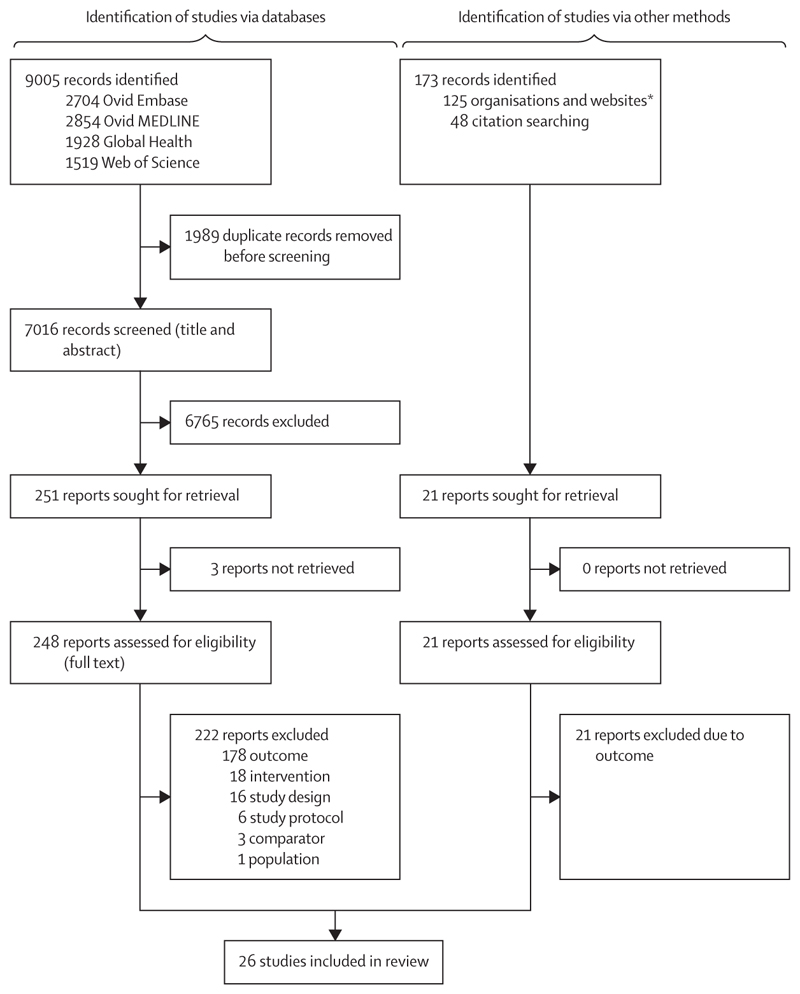
PRISMA flow diagram for study selection *Virtual Health Library, Global Index Medicus, World Bank Open Knowledge Repository, Food and Agriculture Organization, WHO, World Food Programme, Pan American Health Organization, and Instituto Brasileiro de Defesa do Consumidor were searched using keywords and filters.

Our database search identified 7016 unique records, of which 248 reports were retrieved after title and abstract screening, and 26 remained after full-text review (figure 1). At the full-text stage, most reports were excluded because of one-sided or continuous outcomes that could not be attributed to undernutrition or overnutrition. Study design and reported outcomes for each study are presented (table 1). All studies reported DBM at the population-level. One study additionally assessed DBM at the household-level,^55^ and one at the individual-level.^40^ Quality according to the EPHPP rating was high in 14 studies,^32–36,38,40,43–46,50,52,54^ moderate in five studies,^30,31,39,42,53^ and low in seven studies (appendix 4 p 5).^37,41,47–49,51,55^

Ten studies covered interventions in Latin America,^37,38,42–44,51–55^ nine in Africa,^33–36,39,40,46,47,50^ six in Asia,^30–32,41,45,48^ and one in Oceania (table 2).^49^ Nutrition-specific studies cover 13 MCH interventions and seven school-based interventions. Among the MCH interventions, eight targeted the food environment by offering food-based or nutritional supplements,^32–39^ and five solely employed behavioural strategies such as parenting classes or nutrition education.^40–44^ Among MCH interventions, 11 targeted undernutrition,^32–41^ one aimed to improve gestational weight gain,^44^ and one targeted the DBM.^42^ Among school-based programmes, four modified the food environment,^45,47–49^ making healthy foods more available or affordable; two were behavioural interventions related to nutrition education and physical activity promotion;^50,51^ and one employed either nutritional supplementation or behaviour change strategies depending on the intervention group.^46^ Three school-based programmes focused on the DBM,^46,47,50^ three on overweight and obesity,^48,49,51^ and one on undernutrition.^45^ We further included six studies on nutrition-sensitive interventions. Four of these studies evaluated CCTs delivered to families with young children and implemented by the governments of Mexico,^52^ Colombia,^53^ and Peru;^54,55^ and two studies evaluated other social policies—ie, a national education reform in Türkiye and a family planning service to women in Bangladesh (table 2).^30,31^

**Figure 2:**
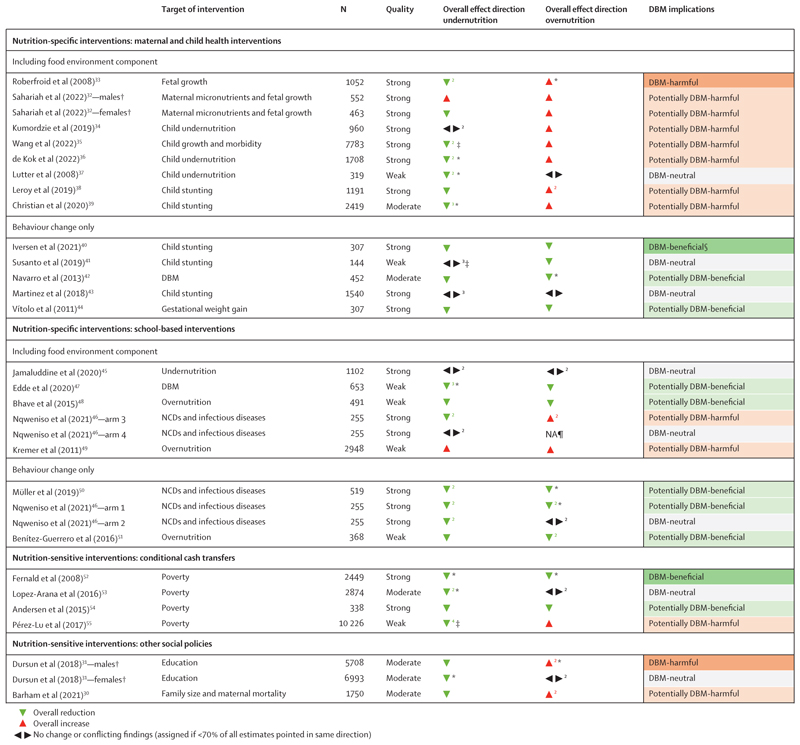
Effect direction plot summarising findings of included studies Overall effect direction shows results from vote counting. DBM=double burden of malnutrition. NA=not applicable. NCD=non-communicable disease. Numbers indicate the number of outcomes contributing to overall effect direction. *One statistically significant effect estimate. †Sex-stratified results are reported only when no overall estimate was available and there were different effect directions for males and females. ‡Two statistically significant effect estimates. §Iversen (2021) was classified as DBM-beneficial because DBM at the individual level (concurrent stunting and overweight within the same child) was significantly reduced, while stunting and overweight were reduced separately, but not significantly. ¶No effect estimates available due to low prevalence of overnutrition in sample.

MCH interventions were DBM-beneficial (n=1),^40^ potentially DBM-beneficial (n=2),^42,44^ DBM-neutral (n=3),^37,41,43^ potentially DBM-harmful (n=6),^32–36,38,39^ and DBM-harmful (n=1; figure 2).^33^ All interventions with DBM-harmful or potentially DBM-harmful effects were related to increased childhood or maternal overweight, included the provision of food-based or nutritional supplements to pregnant women, and were evaluated in high-quality or moderate-quality studies. For example, micronutrient supplementation to pregnant women to enhance fetal growth increased the risk of large-for-gestational age (odds ratio [OR] 1·58, 95% CI 1·04–2·38) in Burkina Faso.^33^ Similarly, providing monthly fortified food rations to pregnant women led to greater postpartum weight retention in Guatemala.^38^ For food-assisted or supplement-assisted MCH interventions, sign test results indicated that 89% of all overnutrition outcomes were negatively influenced (table 3). In contrast, MCH interventions that were potentially DBM-beneficial followed behavioural strategies, most of which included educational sessions on infant and young child feeding practices.^40,42,44^

Among school-based interventions, four were potentially DBM-beneficial,^46–48,51^ one DBM-neutral,^45^ and one potentially DBM-harmful.^49^ A high-quality study in South Africa evaluated behavioural interventions as part of the Disease, Activity, and Schoolchildren’s Health (DASH) programme, finding a reduction in overweight (OR 0·21, 95% CI 0·07–0·66). Stunting (0·76, 0·09–6·53) and anaemia (0·93, 0·38–2·30) were not significantly affected.^50^ A similar behavioural intervention in other South African schools, also part of DASH, significantly reduced body fat by 10% in initially overweight participants; BMI showed an increase in initially underweight individuals; however, the limited number of people initially underweight did not allow statistical testing.^46^ When the same intervention was combined with micronutrient supplementation, the direction of effect on overweight participants changed, although not significantly.^46^ Two low-quality studies evaluated interventions that provided support and incentives to increase the availability of healthy foods in schools in combination with behavioural strategies, resulting in reduced anaemia (0·44, 0·30–0·65).^47^ Overweight in Burkina Faso (0·63, 0·19–2·10)^47^ and underweight (0·92, 0·61–1·37) and overweight (0·85, 0·43–1·70) in India^48^ did not significantly change. For behavioural school-based interventions, the sign test reveals a 100% reduction in undernutrition outcomes (p=0·02), indicating a greater reduction than would be expected by chance. Overnutrition outcomes decreased by 86%, although not reaching statistical significance (p=0·13); school-based interventions that targeted the food environment showed no consistent direction of effect (table 3).

One nutrition-sensitive CCT was DBM-beneficial,^52^ one potentially DBM-beneficial,^54^ one DBM-neutral,^53^ and one potentially DBM-harmful.^55^ A high-quality evaluation of the Mexican CCT Oportunidades showed a significant 10% decrease in stunting and a significant 8% decrease in overweight.^52^ A high-quality study of the Peruvian CCT Juntos suggested a potential dual benefit for children, with non-significant overall reductions in both stunting and overweight, a significant 23% reduction in overweight in girls, and a reduction in height-for-age Z score in boys (0·43, 95% CI 0·09–0·77).^54^ A separate low-quality study on Juntos reported a significant 61% reduction in maternal underweight and a 7% decrease in child anaemia but showed a non-significant 6% increase in maternal overweight.^55^ Although 86% of all undernutrition outcomes of CCTs were improved, there was not enough evidence to indicate that this is not due to chance (p=0·13; table 3).

Among other nutrition-sensitive social policies, a national education reform in Türkiye was DBM-harmful in men due to increased obesity (OR 1·04, 95% CI 1·01–1·06) 18 years after its implementation.^31^ Similarly, a family planning service in Bangladesh was potentially DBM-harmful in women of reproductive age.^30^ Although the programme led to a decrease in underweight, it was also linked to a rise in overweight; however, the estimates on both sides did not reach statistical significance.^30^

## Discussion

This systematic review summarises evidence on the effect of nutrition-specific and nutrition-sensitive interventions on undernutrition and overnutrition. Based on 26 studies, most of which were of high quality or moderate quality, we identified risks and opportunities for double-duty actions. Nutrition-specific MCH interventions providing food-based or nutritional supplements improved child underweight and growth but indicated a potential risk of inadvertently increasing maternal or child overweight. In contrast, interventions targeting nutrition-related behaviours in mothers, infants, or schoolchildren and modifying school food environments showed lower risks of unintended consequences and potential opportunities for double-duty actions. Among nutrition-sensitive interventions, two evaluations of CCTs suggested beneficial effects on the DBM in children, but one study raised concerns about potential harmful effects on maternal overweight. Evaluations of two other social policies indicated unintended long-term consequences related to overweight or obesity.

We found seven MCH studies that provided food-based or nutritional supplements during pregnancy, six of which were of high quality, suggesting a harmful or potentially harmful effect on the DBM due to increased maternal or child overnutrition.^32–36,38,39^ Six studies also identified improved undernutrition-related outcomes,^33,35–39^ reaffirming the well established importance and beneficial effect of food-based or supplement-based MCH interventions in alleviating child underweight and promoting growth.^56,57^ However, in a rapidly evolving food environment with rising obesity rates in women and children younger than 5 years,^21^ the incorporation of dual objectives is essential to ensure that tackling child undernutrition does not exacerbate unhealthy weight gain in mothers and obesity in children. Given that the first 1000 days present a prime window for undernutrition interventions, and the emerging evidence of its importance in preventing obesity, we emphasise prioritising and leveraging dual benefits through MCH interventions with careful consideration of the nutritional context.^58,59^

Schools have been repeatedly described as a promising platform for achieving double-duty objectives, as interventions can be delivered during a period that is considered nutritionally vulnerable due to the rapid physiological and psychological changes of childhood and adolescence.^13,60,61^ Healthy dietary habits and increased levels of physical activity adopted in youth tend to be maintained, leading to long-lasting health benefits.^62,63^ Although most school-based interventions in this review were potentially DBM-beneficial, our conclusions were based on effect directions only, given the absence of statistically significant outcomes. Most interventions had follow-up periods of only less than a year, with the shortest being 3 months, potentially explaining the limited effect on anthropometry. A recent systematic review of various school-based interventions targeting DBM found that 75% of studies had a follow-up period of less than 1 year.^64^ The study also indicated a mixed effect on anthropometric outcomes, with multi-component interventions showing more favourable diet-related outcomes than nutrition education alone, suggesting the potential of integrated school-based approaches and a possible lag in observing anthropometric changes.^64^

Two high-quality studies evaluating CCTs in Peru and Mexico suggested beneficial effects on DBM in children,^52,54^ whereas a low-quality study in Peru indicated potential harmful effects on maternal overweight.^55^ Consistent with our findings, a Cochrane review established the effectiveness of CCTs in reducing stunting and underweight in LMICs, but few studies have examined their effects on overnutrition.^65,66^ An assessment of the Mexican CCT Oportunidades, not meeting our inclusion criteria due to reporting of single-sided outcomes, associated the programme with higher rates of overweight, obesity, and diastolic blood pressure in adults.^67^ The effect was moderated by greater exposure to conditionalities, including health screening and educational workshops, and it was hypothesised that components other than cash transfers (eg, distribution of fortified food supplements) contributed to the adverse effects.^67^ Despite challenges in assessing the independent effects of CCT benefits and conditionalities, more evidence is needed to understand how different components of CCTs affect various target groups and different forms of malnutrition.

We observe DBM-harmful effects for two long-term studies on nutrition-sensitive social policies.^30,31^ In Bangladesh, a family planning service reduced maternal underweight after 19 years but increased the risk of overweight in the same target group after 35 years.^30^ The authors suggested that unobserved factors related to a faster nutrition transition and an increased secular rise in BMI in the intervention areas might have offset the long-term benefits.^30^ Short-term evaluations of family planning interventions have shown their effectiveness in reducing undernutrition and their potential as an economic lever for ensuring quality nutrition for all family members.^15,57,68^ In Turkey, an education reform that increased compulsory schooling was associated with increased obesity in men 18 years after implementation, potentially explained by elevated computer use and a sedentary lifestyle.^31^ Our review revealed potential long-term adverse effects and differential effects over different evaluation periods of nutrition-sensitive social policies. As multiple forms of malnutrition can manifest differently over the life course, the effects on the DBM could vary depending on the follow-up period, emphasising the need for both short-term and long-term evaluations.^60^

Our review offers insights to harness double-duty opportunities and address risks in nutrition interventions. First, for interventions providing food-based or nutritional supplements, incorporating double-duty objectives is crucial to prevent potential spillover effects on overnutrition. This approach might involve integrating postpartum weight management into MCH programmes or tailoring supplementation based on recipients’ dietary needs, as exemplified by India’s Integrated Child Development Service, which transitioned from uniform fortified food prescriptions to prescriptions based on age and physiological condition.^69^ Second, to maximise the leverage of schools as a platform for mitigating the DBM, it is vital to guarantee accessibility, availability, and affordability of nutritious foods in schools—this should be coupled with the provision of educational support to promote the establishment of healthy habits of children and adolescents. Third, integrating nutritional targets into nutrition-sensitive policies could amplify effect and mitigate unintended consequences. Despite not being initially designed for tackling malnutrition, these programmes have the potential to reduce the DBM by addressing common underlying drivers and creating enabling environments. A previous *Lancet* review on MCH nutrition suggests that improved nutrition can also support nutrition-sensitive programmes in achieving their inherent objectives, fostering reinforcing cycles.^15^ Finally, to ensure the effectiveness of CCTs in settings undergoing nutrition transition, it is essential to ensure that the additional household income is not spent on unhealthy food. This endeavour can be supported by promoting a healthy food environment, encouraging better choices through other fiscal policies, or adjusting CCT benefits, such as providing healthy food vouchers.^70–72^

There are several limitations to our systematic review. First, as a necessity for assessing the effect of interventions on the DBM, we only included studies that reported double-sided outcomes. Therefore, selective reporting bias was unavoidable. Second, due to the heterogeneity of interventions and outcomes, we were unable to conduct a meta-analysis. However, adhering to Cochrane guidelines, we synthesised our findings using vote counting, ensuring a transparent presentation of the available evidence underlying our conclusions.^28^ Third, we were unable to do subgroup analyses, as only a few studies reported stratified results. Nonetheless, we extracted effect sizes for all subgroups and mentioned variations from the overall effect in the results. Fourth, our search had linguistic constraints, although we managed to capture 97% of all available literature from searched databases by including studies in English, Spanish, Portuguese, and French. Although we searched for grey literature through multiple established sources, our search was not exhaustive. However, given our inclusion criteria for rigorous study designs and the methodological limitations often present in health policy evaluations found in grey literature, it is unlikely that many studies were missed.^73^ Fifth, the quality assessment and effect estimates were independently obtained by two reviewers, but the remaining descriptive study information was extracted by NAE and reviewed by GCA, potentially introducing a risk of bias or oversight. Finally, for some interventions, undernutrition and overnutrition outcomes might be reported in different studies, which are not included in the review. To address this limitation, we searched citations in all included studies and discussed any additional studies on the same interventions with conflicting findings. Despite limitations, this is the first review to respond to WHO’s call to identify risks and opportunities for double-duty actions from previous nutrition interventions.^14^

We conclude that there is considerable potential for adapting pre-existing nutrition interventions to mitigate the escalating challenges posed by the DBM. In settings undergoing rapid nutrition transition, food-based or supplement-based MCH interventions appear to require specific policy attention, with a crucial need to pursue dual objectives and to ensure that they do not inadvertently increase overweight or obesity. Behavioural strategies targeting mothers and children and the promotion of healthy school food environments represent promising opportunities for double-duty actions in LMICs. We further emphasise the importance of consistently measuring and reporting both undernutrition and overnutrition outcomes in all nutrition interventions, regardless of the intervention’s target and in line with the indicators established under Sustainable Development Goal 2.2, which aims to eliminate all forms of malnutrition.^74^ Increased reports of the two-sided effects of nutritional interventions will generate required evidence for informed policy and programme development. For this goal to be achieved, more research resources should be directed towards LMICs, where the DBM poses the greatest threat to public health due to rapidly changing food environments and lifestyle practices.

## Supplementary Material

Appendix 1

Appendix 2

Appendix 3

Appendix 4

## Figures and Tables

**Table 1:** Study design, publication language, and reported outcomes of included studies

	EPOC criteria met	Study design	Language	Stunting	Thinness	Wasting	Under-weight	Anaemia	Over-weight	Obesity	Continuous outcomes	DBM-level of measured outcomes
**Nutrition-specific interventions: maternal and child health interventions**												
Including food environment component												
Roberfroid et al (2008)^33^	Yes	RCT	English	Yes	No	No	Yes	No	Yes*	No	Birthweight; birth length; Rohrer index; haemoglobin; arm, chest, and head circumferences	Population-level
Sahariah et al (2022)^32^	Yes	RCT	English	Yes	No	Yes	Yes	No	Yes	No	HAZ, BMIZ, skinfolds, fat mass, lean mass, % fat mass, and cardiometabolic risk markers	Population-level
Kumordzie et al (2019)^34^	Yes	RCT	English	Yes	No	No	Yes	No	Yes	No	HAZ, WAZ, BMIZ, MUAC, % fat mass, % FFM, height, weight, and MUAC	Population-level
Wang et al (2022)^35^	Yes	RCT	English	Yes	No	No	Yes	No	Yes	No	Birthweight, birth length, and haemoglobin concentration	Population-level
de Kok et al (2022)^36^	Yes	RCT	English	Yes	No	No	Yes	No	Yes	No	Weight and height	Population-level
Lutter et al (2008)^37^	Yes	Controlled before–after	English	Yes	No	Yes	Yes	Yes	Yes	No	Weight and height	Population-level
Leroy et al (2019)^38^	Yes	Cluster RCT	English	No	No	No	Yes	No	Yes	Yes	BMI and weight	Population-level
Christian et al (2020)^39^	No^†^	Controlled before–after	English	Yes	No	Yes	No	Yes	Yes	No	HAZ, WHZ, BMIZ, haemoglobin concentration, length, weight, MUAC, and head circumference	Population-level
Behaviour change only												
Iversen et al (2021)^40^	Yes	Cluster RCT	English	Yes	No	Yes	No	No	Yes	No	None	Individual-level, population-level
Susanto et al (2019)^41^	Yes	Cluster RCT	English	Yes	No	Yes	Yes	No	Yes	No	None	Population-level
Navarro et al (2013)^42^*	Yes	Non-randomised trial	English	Yes	No	No	No	No	Yes	No	HAZ and BMIZ	Population-level
Martinez et al (2018)^43^	Yes	RCT	English	Yes	No	Yes	Yes	Yes	Yes	No	WAZ, WHZ, BMIZ, HAZ, and head circumference-for-age Z score	Population-level
Vitolo et al (2011)^44^	Yes	RCT	Portuguese	No	No	No	Yes	No	Yes	No	Weight	Population-level
**Nutrition-specific interventions: school-based interventions**												
Including food environment component												
Jamaluddine et al (2020)^45^	Yes	Cluster RCT	English	Yes	Yes	No	No	Yes	Yes	Yes	BMIZ, HAZ, and haemoglobin concentration	Population-level
Nqweniso et al (2021)^46^	No^†^	Cluster RCT	English	No	No	No	No	No	No	No	BMI, BMIZ, and % fat mass stratified by nutritional status at baseline (underweight, healthy weight, and overweight)	Population-level
Elkhouri Edde et al (2020)^47^	Yes	Controlled before–after	English	Yes	Yes	No	No	Yes	Yes	No	None	Population-level
Bhave et al (2016)^48^	No^‡^	Non-randomised trial	English	No	No	No	Yes	No	Yes	No	Height, BMI, BMIZ, and waist circumference	Population-level
Kremer et al (2011)^49^	Yes	Non-randomised trial	English	No	Yes	No	No	No	Yes	Yes	Weight, BMI, BMIZ, and % fat mass	Population-level
Behaviour change only												
Müller et al (2019)^50^	Yes	Cluster RCT	English	Yes	No	No	No	Yes	No	No	BMIZ and skinfolds thickness	Population-level
Benítez-Guerrero et al (2016)^51^	Yes	Controlled before–after	Spanish	No	No	No	Yes	No	Yes	Yes	BMI	Population-level
**Nutrition-sensitive interventions: conditional cash transfers**												
Fernald et al (2008)^52^†	Yes	Cluster RCT	English	Yes	No	No	No	No	Yes	No	HAZ, BMI-for-age percentile, and haemoglobin concentration	Population-level
Lopez-Arana et al (2016)^53^	Yes	Controlled before–after	English	Yes	Yes	No	No	No	Yes	Yes	BMIZ, HAZ, and BMI	Population-level
Andersen et al (2015)^54^	No^‡^	Prospective cohort	English	Yes	No	No	No	No	Yes	No	HAZ and BMIZ	Population-level
Pérez-Lu et al (2017)^55^	Yes	Controlled before–after	English	No	No	Yes (children)	Yes (children and mothers)	Yes (mothers)	Yes (mothers)	No	None	Household-level, population-level
**Nutrition-sensitive interventions: other social policies**												
Dursun et al (2018)^31^	Yes	Controlled before—after	English	No	No	No	Yes	No	Yes	No	BMI	Population-level
Barham et al (2021)^30^	Yes	Non-randomised trial	English	No	No	No	Yes	No	Yes	Yes	BMI	Population-level

Stunting refers to low height-for-age (>2 SD below the WHO Child Growth Standards median). Thinness refers to low BMI-for-age (>2 SD below the WHO Child Growth Standards median). Wasting refers to low weight-for-height (>2 SD below the WHO Child Growth Standards median). Underweight refers to low weight-for-age (>2 SD below the WHO Child Growth Standards median). Anaemia refers to low haemoglobin. Overweight (weight-for-height >2 SD above the WHO Child Growth Standards median in children younger than 5 years, BMI-for-age ≥1 SD above for children aged 5–19 years, and BMI ≥25 and <30 kg/m^2^ in adults) and obesity (weight-for-height >3 SD above WHO Child Growth Standards in children younger than 5 years, BMI-for-age ≥2 SD above in children aged 5–19 years, BMI ≥30 kg/m^2^ in adults) refer to high BMI-for-age or high weight-for-height. DBM-level indicates the target population in which outcomes were measured; population-level indicates outcomes measured within the same group; household-level indicates outcomes measured in different household members; individual-level indicates multiple outcomes assessed simultaneously within the same person. BMIZ=BMI-for-age Z score. DBM=double burden of malnutrition. EPHPP=Effective Public Health Practice Project. EPOC=Effective Practice and Organisation of Care. FFM=fat-free mass. HAZ=height-for-age Z score. MUAC=mid-upper-arm-circumference. RCT=randomised controlled trial. WAZ=weight-for-age Z score. WHZ=weight-for-height Z score.

* Definitions for overweight differ in these studies.

† Only one intervention and one control site per intervention.

‡ Prospective cohort studies.

**Table 2:** Description of nutrition-specific and nutrition-sensitive interventions investigated in included studies

	Country (region)	Implementing organisation	Aim of intervention	Target population	Intervention description	Intervention duration and evaluation follow-up time
**Nutrition-specific interventions: maternal and child health interventions**						
Including food environment component						
Roberfroid et al (2008)^33^	Burkina Faso (Houndé)	Research organisation	Improve fetal growth	Pregnant women, outcomes measured in children	Daily provision of UNICEF, WHO, and UNU international multiple micronutrient preparation (UNIMAP) until 3 months after delivery	Duration: 1·5 years, follow-up: 24 h after childbirth
Sahariah et al (2022)^32^	India (Mumbai)	Research organisation	Maternal micronutrient status, fetal growth, and LBW	Pregnant women, outcomes measured in children aged 5–10 years	Provision of daily food-based micronutrient supplement 3 months before conception and during pregnancy in the form of a snack made in local test kitchens (Mumbai Maternal Nutrition Project)	Duration: 5 years, follow-up: 5–10 years after start
Kumordzie et al (2019)^34^	Ghana (Yilo and Lower Manya Krobo)	Research organisation	Improve child growth and prevent undernourishment	Pregnant women older than 18 years and offspring once aged 6 months, outcomes measured in children	Administration of daily lipid-based nutrient supplements during pregnancy and to infants (International Lipid-Based Nutrient Supplements Project)	Duration: 2 years, follow-up: 4–6 years after childbirth
Wang et al (2022)^35^	Tanzania (Dar es Salaam)	Research organisation	Improve child growth and prevent morbidities	Pregnant women (starting from second trimester), outcomes measured in children	Provision of daily oral supplements containing multiple micronutrients; the doses were twice the recommended dietary allowance for vitamin E and 6–10 times for vitamin C and B vitamins	Duration: from 2nd trimester of pregnancy to 6 weeks postpartum, follow-up: at childbirth
de Kok et al (2022)^36^	Burkina Faso (Houndé)	Research organisation	Improve birth outcomes	Intervention to pregnant girls and women aged 15–40 years, outcomes measured in children	Administration of daily energy-protein supplement fortified with multiple micronutrients and an iron-folic acid tablet (MISAME-III trial); the supplements were consumed under the supervision of trained community workers during home visits; the workers also encouraged pregnant women to attend at least four antenatal care consultations	Duration: start to end of pregnancy, follow-up: 12 h after childbirth
Lutter et al (2008)^37^	Ecuador (periurban and rural communities)	Government	Prevent undernutrition	Children aged 6–11 months	Provision of daily fortified complementary food (Mi Papilla), monitoring and evaluation of intake, training of health workers in IYCF, and raising awareness for nutrition in early childhood to families (National Food Nutrition Program 2000)	Duration: 11 months, follow-up: 11 months after start
Leroy et al (2019)^38^	Guatemala (Alta Verapaz)	NGO	Prevent undernutrition and micronutrient deficiencies	Women older than 18 years and 3–7 months pregnant	Provision of monthly rations to households, containing fortified foods or supplements conditional on attending behaviour change communication sessions and health check-ups (PROCOMIDA)	Duration: 2 years, follow-up: 2 years after start
Christian et al (2020)^39^	Malawi (rural district)	Government	Reduce child stunting	Children aged 6–23 months	Distribution of a daily small quantity lipid-based supplement to children and monthly counselling sessions on IYCF and WASH practices to primary care givers using a social and behaviour change communication strategy (The Right Foods at the Right Time)	Duration: 3 years, follow-up: 3 years after start
Behaviour change only						
Iversen et al (2021)^40^	Uganda (Kabale and Kisoro)	Research organisation	Prevent child stunting	Mothers of children aged 6–8 months, outcomes measured in children	Three group education sessions focusing on cooking, hygiene, child stimulation demonstrations, and education on breast and complementary feeding to mothers followed by monthly village meetings	Duration: 6 months, follow-up: yearly up to 5–6 years after start
Susanto et al (2019)^41^	Indonesia (Jember, East Java)	Research organisation	Promote child growth and development	Parents of children aged 0–72 months, outcomes measured in children	Fortnightly parenting classes by local nurses at health centres focusing on child nutrition, immunisation, IYCF, height and weight monitoring, child health, communication, child stimulation, and schooling needs	Duration: 14 weeks, follow-up: 14 weeks after start
Navarro et al (2013)^42^	Dominican Republic (eight different areas)	Community organisation	Reduce undernutrition and overweight	Pregnant women, outcomes measured in children aged 3–24 months	Fortnightly group classes and monthly home visits conducted by a community organisation for pregnant women focusing on IYCF, immunisation, micronutrient supplementation, child health, child stimulation, and growth monitoring	Duration: 2 years, follow-up: 3–24 months after childbirth
Martinez et al (2017)^43^	Bolivia (El Alto)	NGO	Improve feeding practices, hygiene, and nutritional status	Households with pregnant women and children younger than 12 months, outcomes measured in children	Monthly home visits by health community workers focusing on IYCF and WASH practices through a behaviour change strategy based on participatory play (Community Child Nutrition project)	Duration: 30 months, follow-up: 30 months after start
Vítolo et al (2011)^44^	Brazil (Porto Alegre)	Research organisation	Improve gestational weight gain	Pregnant women with a gestational age of <29 weeks	Pregnant women were given dietary guidelines focusing on improving eating habits and controlling weight gain; these guidelines were individually tailored to the nutritional status of the women before pregnancy; a follow-up session was done 1 month later to consolidate and reinforce the initial dietary recommendations	Duration: 2 sessions within 1 month, follow-up: on average 5 months later
**Nutrition-specific interventions: school-based interventions**						
Including food environment component						
Jamaluddine et al (2020)^45^	Lebanon (Palestinian refugee schools)	Community organisation	Improve nutritional and educational outcomes	Children aged 5–15 years	Establishment of two community kitchens as social enterprises run by community-based women’s organisations, preparing daily subsidised snacks for UNRWA schools, with snacks consisting of at least three food groups (dairy, complex carbohydrates, meat, vegetables, fruit)	Duration: 8 months, follow-up: 8 months after start
Nqweniso et al (2021)^46^	South Africa (Port Elizabeth)	Research organisation	Reduce NCDs and infectious diseases	Children aged 8–11 years	Classroom education (health, hygiene, and nutrition); provision of micronutrient supplements, deworming, and PA intervention (two PE classes, one movement to music class per week, in-class activity breaks and setting up movement and active game stations in school); four intervention groups with different combinations (PA and deworming; PA, education, and deworming; education, supplementation, and deworming; and PA, education, supplementation, and deworming); intervention is part of Disease, Activity and Schoolchildren's Health multidimensional PA intervention programme	Duration: 10 weeks, follow-up: 4 months after start
Elkhouri Edde et al (2020)^47^	Burkina Faso (Ouagadougou)	Multilateral organisation (WHO)	Reduce undernutrition and overweight	Children aged 8–14 years	Accreditation system with label nutrition friendly conditional on nutrition policies, capacity and awareness building, nutrition and health promoting curricula, supportive school environment, school nutrition, and health services (Nutrition-Friendly School Initiative)	Duration: 5 years, follow-up: 5 years after start
Bhave et al (2015)^48^	India (Pune)	Research organisation	Reduce adiposity and improve fitness and lifestyle	Children aged 7–10 years	Increase of PE lessons to six per week, making PE a scoring subject; daily breathing exercises; support of school kitchens to develop healthy meals; removal of fast food sellers outside schools; and termly health workshops	Duration: 5 years, follow-up: 5 years after start
Kremer et al (2011)^49^	Fiji (Nasinu on Viti Levu)	Government	Reduce unhealthy weight gain and obesity-promoting behaviours	Adolescents aged 3–18 years	Promotion of reducing the consumption of sugary drinks and high-energy snacks and increasing PA and fruit consumption; workshops to empower parents to support their children; social marketing; and ensuring that healthy meals are provided in school canteens (Pacific Obesity Prevention in Communities project)	Duration: 2 years, follow-up: 2 years after start
Behaviour change only						
Müller et al (2019)^50^	South Africa (Port Elizabeth)	Research organisation	Reduce NCDs and infectious diseases	Children aged 9–12 years	PA intervention with two PE classes and one movement to music class per week, in-class activity breaks, and set up of movement and active game stations in school, part of a multidimensional PA programme (Disease, Activity and Schoolchildren's Health)	Duration: 2 x 10 weeks, follow-up: 9 months after start
Benítez-Guerrero et al (2016)^51^	Mexico (Nayarit)	Research organisation	Reduce overweight	Children aged 9–11 years	Development of two health education programmes focused on nutrition and PA in 12 different primary schools	Duration: 3 months, follow-up: 3 months after start
**Nutrition-sensitive interventions: conditional cash transfers**						
Fernald et al (2008)^52^	Mexico	Government	Poverty reduction and human capital development	Families with children aged 4–68 months, outcomes measured in children	CCT (Oportunidades) with amount depending on household composition and conditional on family member receiving preventive care and children attending >85% of all school lessons; additional fortified food supplements given to mothers, children aged 0–2 years, and malnourished children aged 3–5 years	Duration: 5 years, follow-up: 5 years after start
Lopez-Arana et al (2015)^53^	Colombia	Government	Poverty reduction and human capital development	Families with children younger than 18 years, outcomes measured in children	CCT (Familias en acción) with amount depending on household composition and conditional on children younger than 7 years receiving vaccination and attending growth and development examinations, and children aged 7–17 years attending attending >80% of all school lessons	Duration: 4 years, follow-up: 4 years after start
Andersen et al (2015)^54^	Peru	Government	Poverty reduction and human capital development	Families with children aged 4–6 years, outcomes measured in children	CCT (Juntos) with fixed amount for every household and conditional on children younger than 5 years, pregnant or lactating women attending health care, and children younger than 5 years attending >85% of all school lessons	Duration: 4 years, follow-up: 4 years after start
Pérez-Lu et al (2017)^55^	Peru	Government	Poverty reduction and human capital development	Families with children aged 4–6 years, outcomes measured in children and mothers	CCT (Juntos) with fixed amount for every household, conditional on children younger than 5 years, pregnant and lactating woman attending health care, and children younger than 5 years attending >85% of all school lessons	Duration: 3 years, follow-up: 3 years after start
**Nutrition-sensitive interventions: other social policies**						
Dursun et al (2018)^31^	Türkiye	Government	Extend schooling duration	Turkish citizens aged 8–34 years	National education reform increasing compulsory schooling from age 5 to 8 years	Duration: 18 years, follow-up: 18 years after start
Barham et al (2021)^30^	Bangladesh (Matlab)	Research organisation	Reduce family size and maternal mortality	Girls and women aged 4–39 years	Family planning intervention providing advice and means for contraception to married women by community workers	Duration: 35 years, follow-up: 35 years after start

CCT=conditional cash transfer. IYCF=infant and young child feeding. LBW=low birthweight. NCD=non-communicable disease. NGO=non-governmental organisation. PA=physical activity. PE=physical education. UNRWA=UN Relief and Works Agency for Palestine Refugees in the Near East. UNU=UN University. WASH=water, sanitation, and hygiene.

**Table 3:** Effectiveness of intervention categories on undernutrition and overnutrition

	Undernutrition					Overnutrition				
	Outcomes (N)	Proportion (95% CI) of decreased (beneficial) outcomes	p value	Proportion (95% CI) of increased (harmful) outcomes	p value	Outcomes (N)	Proportion (95% CI) of decreased (beneficial) outcomes	p value	Proportion (95% CI) of increased (harmful) outcomes	p value
Maternal and child health interventions (overall)	26	0·69 (0·48–0·86)	0·08	0·08 (0·01–0·25)	<0·01	14	0·29 (0·08–0·58)	0·18	0·57 (0·29–0·82)	0·80
Maternal and child health interventions (including food environment component)	17	0·71 (0·44–0·90)	0·14	0·06 (0·00–0·29)	<0·01	9	0·00 (0·00–0·34)	<0·01	0·89 (0·52–0·99)	0·04
Maternal and child health interventions (behaviour change only)	9	0·67 (0·30–0·93)	0·51	0·11 (0·00–0·48)	0·04	5	0·8 (0·28–0·99)	0·38	0·00 (0·00–0·52)	0·06
School-based interventions (overall)	12	0·58 (0·28–0·84)	0·77	0·25 (0·05–0·57)	0·15	9	0·56 (0·23–0·86)	1·00	0·22 (0·03–0·60)	0·18
School-based interventions (including food environment component)	9	0·44 (0·14–0·79)	1·00	0·22 (0·03–0·60)	0·18	7	0·42 (0·10–0·82)	1·00	0·29 (0·04–0·71)	0·45
School-based interventions (behaviour change only)	7	1·00 (0·59–1·00)	0·02	0·00 (0·00–0·41)	0·02	7	0·86 (0·42–0·97)	0·13	0·14 (0·00–0·57)	0·13
Conditional cash transfers	7	0·86 (0·42–0·97)	0·13	0·14 (0·00–0·58)	0·13	5	0·6 (0·15–0·95)	1·00	0·4 (0·05–0·85)	1·00
Other social policies	2	1·00 (0·16–1·00)	0·50	0·00 (0·00–0·84)	0·50	4	0·00 (0·00–0·60)	0·50	0·5 (0·07–0·93)	0·50

For each intervention category, the proportion of decreased (vs no effect and increased) and increased (vs no effect and decreased) outcomes for undernutrition and overnutrition were computed, as well as the p value and 95% CI using a sign test (binomial probability test). Outcomes contributing to undernutrition included stunting, underweight, thinness, anaemia, low birthweight and small-for-gestational age, and outcomes contributing to overnutrition included overweight, obesity, and macrosomia.

## Data Availability

All data relevant to the study are included in the Article or uploaded as supplementary information.

## Research in context

### Evidence before this study

We searched PubMed and Web of Science using search terms related to or synonymous with “double-duty actions”, “nutrition policies”, “food programmes”, “double burden of malnutrition”, “overnutrition” and “undernutrition” from Jan 1, 2000, to Aug 14, 2023. We identified several systematic reviews examining associations between nutrition interventions and undernutrition or overnutrition separately. Only one scoping review summarised the effect of seven novel double-duty actions, with the conclusion that there is a dearth of studies evaluating policies that explicitly target the double burden of malnutrition (DBM). Another recently published systematic review included 43 studies that evaluated the effect of double-duty and triple-duty actions—ie, actions that concurrently address different forms of malnutrition and environmental sustainability on nutrition-related, climate-related, and health-related outcomes. The review concluded that the variety of outcomes measured prevented comparison of different interventions in terms of their effectiveness. From the available evidence, we concluded that previous reviews focused on interventions that explicitly included double-duty objectives, and that assessing their effectiveness in alleviating the DBM was hampered by the heterogeneity of outcomes measured. However, there is a gap in knowledge about how nutrition interventions, regardless of their objective, affect the DBM, using consistent outcome measures for undernutrition and overnutrition.

### Added value of this study

This systematic review provides important new insights on the effect of nutrition-specific and nutrition-sensitive interventions on both undernutrition and overnutrition outcomes, and it responds to WHO’s call to identify risks and opportunities for double-duty actions from previous nutrition interventions. We identified interventions that have the potential to improve both undernutrition and overnutrition simultaneously, including three behavioural interventions in mothers and children, five school-based interventions, and two conditional cash transfers. We further identified 13 interventions that, while effectively addressing undernutrition, display indications of unintended adverse effects on the DBM concerning overweight and obesity. Most of these interventions were maternal and child health interventions that involved the provision of food-based or nutritional supplements. The large number of studies excluded due to an absence of reported outcomes on both undernutrition and overnutrition highlights that existing evaluations of nutrition interventions are not commonly designed to assess double-sided outcomes.

### Implications of all the available evidence

Since WHO proclaimed the importance of double-duty actions in 2015, considerable attention has been paid to their implementation. Existing nutrition-specific and nutrition-sensitive interventions have the potential to effectively address the DBM. However, some types of interventions that focus on only one dimension of malnutrition carry a higher risk of unintended harm, and might require reframing. There is a need to raise awareness of the risks associated with one-sided interventions. To improve the basis for informed policy decisions, it is crucial to standardise the measurement and reporting of undernutrition and overnutrition outcomes in all nutrition interventions, consistent with the Sustainable Development Goal 2.2, which seeks to eliminate all forms of malnutrition. This standardisation will facilitate the identification and promotion of interventions that maximise benefits and minimise harms on the DBM.
