# Supplementary material for: The effect of nutrition-specific and nutrition-sensitive interventions on the double burden of malnutrition in low-income and middle-income countries: a systematic review
Source: Lancet Glob Health. Author manuscript; Available in PMC 2024 May 31. (PMC7616050; doi:10.1016/S2214-109X(23)00562-4)
Supplement: Appendix 1 [file EMS196083-supplement-Appendix_1.pdf]

THE LANCET
Global Health

Supplementary appendix 1

This translation in Portuguese was submitted by the authors and we reproduce it as supplied. It has not been peer reviewed. *The Lancet*'s editorial processes have only been applied to the original in English, which should serve as reference for this manuscript.

Esta tradução em português foi submetida pelos autores e nós não fizemos quaisquer alterações. Esta versão não foi revista por pares. O processo editorial do The Lancet só foi aplicado à versão original em inglês, que deve servir como referência para este artigo.

Supplement to: Escher NA, Andrade GC, Ghosh-Jerath S, Millett C, Seferidi P.
The effect of nutrition-specific and nutrition-sensitive interventions on the double burden of malnutrition in low-income and middle-income countries: a systematic review. *Lancet Glob Health* 2024; published online Jan 29. https://doi.org/10.1016/S2214-109X(23)00562-4.

O impacto das intervenções específicas e sensíveis à nutrição no duplo fardo da malnutrição nos países de baixo e médio rendimento: uma revisão sistemática

Introdução

Países de baixa e média renda em transição nutricional enfrentam o desafio do crescente aumento na dupla carga de malnutrição (DCM). Embora a Organização Mundial da Saúde aponte a urgência de identificar potenciais riscos e oportunidades de intervenções a fim de mitigar a DCM, evidências científicas sólidas ainda são necessárias. A revisão conduzida no presente estudo tem como objetivo investigar o impacto de intervenções específicas e sensíveis à nutrição na ocorrência concomitante de desnutrição e excesso de peso em países de baixa e média renda.

Métodos

Quatro bases de dados e a literatura cinzenta foram utilizadas como ferramenta de busca para identificação de publicações em inglês, francês, português e espanhol, entre1º de janeiro de 2000 a 14 de agosto de 2023. Estudos elegíveis avaliaram o impacto de intervenções específicas e/ou sensíveis à nutrição em marcadores de desnutrição e excesso de peso. Foram incluídos ensaios clínicos (individuais, de cluster ou não randomizados), séries temporais interrompidas, estudos controlados antes e depois, e estudos de coorte prospectivos. As publicações foram sintetizadas e classificadas como: benéficos à DCM, potencialmente benéficos à DCM, neutros à DCM, potencialmente prejudiciais à DCM e prejudiciais à DCM. A revisão está registrada no PROSPERO, CRD42022320131.

Resultados

No total, 26 estudos foram incluídos, dos quais 20 avaliaram intervenções específicas para nutrição (saúde materno-infantil (SMI) e programas escolares) e seis avaliaram intervenções sensíveis à nutrição (programas de transferência de renda e outras políticas sociais). Sete entre oito estudo de intervenções que forneceram suplementos alimentares ou nutricionais para crianças ou gestantes foram classificadas como potencialmente prejudiciais à DCM, sendo associados ao aumento do excesso de peso materno ou infantil. A maioria dos programas escolares e intervenções de mudança comportamental com foco em SMI foram classificadas como potencialmente benéficos para a DCM. Dois estudos que avaliaram programas de transferências de renda mostraram impactos benéficos na DCM entre crianças, enquanto um estudo indicou efeitos potencialmente prejudiciais sobre o excesso de peso materno. Avaliações de serviços de planejamento familiar e de intervenções educacionais foram consideradas potencialmente prejudiciais à obesidade a longo prazo.

Interpretação

Há uma importante oportunidade para redesenhar intervenções nutricionais existentes visando reduzir a crescente prevalência da DCM em países de baixa e média renda. A rápida transição nutricional requer uma atenção política específica para garantir que os programas de SMI baseados na suplementação de alimentos ou nutrientes não aumente involuntariamente o excesso de peso materno ou infantil. É também essencial que estudos de intervenção nutricional reportem simultaneamente desfechos em desnutrição e excesso de peso, para melhor identificar e promover intervenções que maximizem benefícios e minimizem danos relacionados à DCM.

Financiamento

President's Scholarship (Imperial College London) e National Institute for Health and Care Research (NIHR).
