# Supplementary material for: The effect of nutrition-specific and nutrition-sensitive interventions on the double burden of malnutrition in low-income and middle-income countries: a systematic review
Source: Lancet Glob Health. Author manuscript; Available in PMC 2024 May 31. (PMC7616050; doi:10.1016/S2214-109X(23)00562-4)
Supplement: Appendix 2 [file EMS196083-supplement-Appendix_2.pdf]

THE LANCET
Global Health

Supplementary appendix 2

This translation in Spanish was submitted by the authors and we reproduce it as supplied. It has not been peer reviewed. *The Lancet*'s editorial processes have only been applied to the original in English, which should serve as reference for this manuscript.

Los autores nos proporcionaron esta traducción al español y la reproducimos tal como nos fue entregada. No la hemos revisado. Los procesos editoriales de *The Lancet* se han aplicado únicamente al original en inglés, que debe servir de referencia para este manuscrito.

Supplement to: Escher NA, Andrade GC, Ghosh-Jerath S, Millett C, Seferidi P. The effect of nutrition-specific and nutrition-sensitive interventions on the double burden of malnutrition in low-income and middle-income countries: a systematic review. *Lancet Glob Health* 2024; published online Jan 29. https://doi.org/10.1016/S2214-109X(23)00562-4.

El impacto de las intervenciones específicas y sensibles a la nutrición en la doble carga de malnutrición en países de ingresos bajos y medios: una revisión sistemática

Contexto

Los países de ingresos bajos y medios (PIBM) en transición nutricional se enfrentan a una creciente doble carga de malnutrición (DCM). La Organización Mundial de la Salud ha recomendado la identificación de riesgos y oportunidades en las intervenciones nutricionales para mitigar la DCM, pero faltan pruebas sólidas. Esta revisión resume el impacto de las intervenciones específicas y sensibles a la nutrición en la desnutrición y la sobrealimentación en los PIBM.

Métodos

Buscamos en cuatro bases de datos principales y en la literatura gris publicaciones en inglés, francés, portugués y español desde el 1 de enero de 2000 hasta el 14 de agosto de 2023. Los estudios elegibles evaluaron intervenciones específicas y/o sensibles a la nutrición en desnutrición y sobrealimentación utilizando diseños de estudio robustos (ensayos aleatorios individuales, aleatorios por conglomerados, no aleatorios, series temporales interrumpidas, estudios controlados antes-después y cohortes prospectivas). Se resumieron los estudios en forma narrativa y, utilizando el método de recuento de votos, se asignó un impacto en la DCM (beneficioso, potencialmente beneficioso, neutro, potencialmente desfavorable y desfavorable). La revisión está registrada en PROSPERO, CRD4202232320131.

Resultados

Identificamos 26 estudios que evaluaron 20 intervenciones específicas de nutrición (programas de salud materno-infantil (SMI) y escolares) y seis intervenciones sensibles a la nutrición (transferencias condicionales de efectivo y otras políticas sociales). Siete de las ocho intervenciones de SMI que proporcionaron suplementos alimenticios indicaron efectos potencialmente adversos para el DCM, asociados con sobrepeso materno o infantil. La mayoría de los programas escolares y las de intervenciones de SMI dirigidas a cambiar el comportamiento fueron potencialmente beneficiosos para el DCM. Dos evaluaciones de transferencias condicionales de efectivo sugirieron impactos beneficiosos en niños, mientras que una indicó efectos potencialmente adversos en el sobrepeso materno. Las evaluaciones de un servicio de control de la natalidad y de una reforma educativa revelaron efectos adversos en la obesidad.

Interpretación

Las intervenciones nutricionales existentes ofrecen una oportunidad de adaptación para mitigar el crecimiento del DCM. En contextos de rápida transición nutricional, se requiere una atención específica para garantizar que los programas de SMI basados en alimentos o suplementos no aumenten involuntariamente el sobrepeso. Es importante que las futuras evaluaciones de las intervenciones nutricionales informen consistentemente sobre los efectos en la sobrealimentación y la desnutrición para ampliar la base de pruebas y promover intervenciones que minimicen los riesgos de DCM.

Financiación

President's Scholarship (Imperial College London) y National Institute for Health and Care Research (NIHR).
