# Supplementary material for: The effect of nutrition-specific and nutrition-sensitive interventions on the double burden of malnutrition in low-income and middle-income countries: a systematic review
Source: Lancet Glob Health. Author manuscript; Available in PMC 2024 May 31. (PMC7616050; doi:10.1016/S2214-109X(23)00562-4)
Supplement: Appendix 3 [file EMS196083-supplement-Appendix_3.pdf]

THE LANCET
Global Health

Supplementary appendix 3

This translation in French was submitted by the authors and we reproduce it as supplied. It has not been peer reviewed. *The Lancet*'s editorial processes have only been applied to the original in English, which should serve as reference for this manuscript.

Cette traduction en français a été proposée par les auteurs et nous l'avons reproduite telle quelle. Elle n'a pas été examinée par des pairs. Les processus éditoriaux du *Lancet* n'ont été appliqués qu'à l'original en anglais et c'est cette version qui doit servir de référence pour ce manuscrit.

Supplement to: Escher NA, Andrade GC, Ghosh-Jerath S, Millett C, Seferidi P. The effect of nutrition-specific and nutrition-sensitive interventions on the double burden of malnutrition in low-income and middle-income countries: a systematic review. *Lancet Glob Health* 2024; published online Jan 29. https://doi.org/10.1016/S2214-109X(23)00562-4.

L'impact des interventions spécifiques et sensibles à la nutrition sur le double fardeau de la malnutrition dans les pays à revenu faible et intermédiaire : une revue systématique

Contexte

Les pays à revenu faible et intermédiaire (PRFI) en transition nutritionnelle rapide font face à un double fardeau de la malnutrition (DFM) croissant. Les risques et opportunités des interventions nutritionnelles sur le DFM sont trop peu répertoriés. Cette revue résume l'impact des interventions spécifiques et sensibles à la nutrition sur la sous- et suralimentation dans les PRFI.

Méthodes

Nous avons consulté quatre bases de données et la littérature grise de janvier 2000 au 14 août 2023 pour identifier des études en anglais, français, portugais et espagnol qui ont évalué l'impact d'interventions nutritionnelles sur la sous- et suralimentation en utilisant des méthodes d'étude rigoureuses (études individuelles ou de groupe randomisées, études non randomisées, séries temporelles interrompues, études contrôlées avant-après et études de cohortes prospectives). Les études ont été synthétisées narrativement et classées en bénéfiques, potentiellement bénéfiques, neutres, potentiellement nuisibles et nuisibles pour le DFM, selon le décompte des votes. La revue est enregistrée dans PROSPERO, CRD42022320131.

Résultats

Sur les 26 études identifiées, 20 sont des interventions spécifiques à la nutrition (santé maternelle et infantile (SMI), programmes scolaires) et six sont des interventions sensibles à la nutrition (transferts monétaires conditionnels, politiques sociales). Sept des huit interventions de SMI fournissant compléments nutritionnels étaient potentiellement nuisibles pour le DFM, associés à une augmentation du surpoids maternel ou infantile. La plupart des programmes scolaires et des interventions sur le comportement en SMI ont été potentiellement bénéfiques. Deux évaluations de transferts monétaires conditionnels ont suggéré des effets bénéfiques sur le DFM, tandis qu'une a indiqué des effets potentiellement nuisibles sur le surpoids maternel. Les évaluations d'un service de planning familial et d'une réforme éducative ont révélé des effets nuisibles sur l'obésité à long terme.

Interprétation

Les interventions nutritionnelles existantes pourraient être réajustées pour atténuer la croissance du DFM dans les PRFI. Là où la transition nutritionnelle est rapide, une attention particulière est nécessaire pour s'assurer que les programmes de SMI basés sur des compléments alimentaires ne contribuent pas involontairement au surpoids. Un suivi cohérent de l'effet des futures interventions nutritionnelles sur la sous- et suralimentation est crucial pour élargir la base de données et encourager des interventions réduisant les risques associés au DFM.

Financement

President's Scholarship (Imperial College London) et National Institute for Health and Care Research (NIHR).
