# Supplementary material for: The effect of nutrition-specific and nutrition-sensitive interventions on the double burden of malnutrition in low-income and middle-income countries: a systematic review
Source: Lancet Glob Health. Author manuscript; Available in PMC 2024 May 31. (PMC7616050; doi:10.1016/S2214-109X(23)00562-4)
Supplement: Appendix 4 [file EMS196083-supplement-Appendix_4.pdf]

THE LANCET
Global Health

Supplementary appendix 4

This appendix formed part of the original submission and has been peer reviewed. We post it as supplied by the authors.

Supplement to: Escher NA, Andrade GC, Ghosh-Jerath S, Millett C, Seferidi P. The effect of nutrition-specific and nutrition-sensitive interventions on the double burden of malnutrition in low-income and middle-income countries: a systematic review. *Lancet Glob Health* 2024; published online Jan 29. https://doi.org/10.1016/S2214-109X(23)00562-4.

Appendix 4: Supplementary Material

Table of Contents

Appendix 4.1 Supplementary Figures and Tables ... **2**

Supplementary Table 1. Inclusion and exclusion criteria. ... 2
Supplementary Table 2A. Studies not retrieved or excluded from database search, grouped by reason for exclusion. ... 3
Supplementary Table 2B. Studies identified via other methods and excluded, grouped by reason for exclusion. ... 3
Supplementary Figure 1. Conceptual representation of types of interventions included in this review, adapted from a previous framework on nutrition actions. [247] ... 4
Supplementary Figure 2. Quality assessment for included studies (n=26). ... 5
Supplementary Table 3. Effect estimates and 95% CI for categorical outcomes. ... 6
Supplementary Table 4. Effect estimates and 95% CI for continuous outcomes. ... 9
Supplementary Table 5. Covariates that were adjusted for in most adjusted model for each study. ... 12

Appendix 4.2 Search strategy ... **13**

Appendix 4.2.1 Search strategy for Ovid MEDLINE. ... 13
Appendix 4.2.2 Search strategy for Ovid Embase ... 14
Appendix 4.2.3 Search strategy for Ovid Global Health. ... 15
Appendix 4.2.4 Search strategy for Web of Science. ... 16

Appendix 4.3 Data extraction ... **18**

Appendix 4.3.1 Data extraction template for descriptive information of studies. ... 18
Appendix 4.3.2 Data extraction template for categorical variables. ... 18
Appendix 4.3.3 Data extraction template for continuous variables. ... 18
Appendix 4.3.4 Data extraction template for stratified results for categorical variables. ... 19
Appendix 4.3.5 Data extraction template for stratified results for continuous variables. ... 19

Appendix 4.4 Quality assessment template [276] ... **20**

References ... **22**

Appendix 4.1 Supplementary Figures and Tables

Supplementary Table 1. Inclusion and exclusion criteria.

	Inclusion criteria	Exclusion criteria
**Population**	General population and pregnant or lactating people from low-income and middle-income countries	Studies only including participants with diagnosed conditions (e.g. cardiovascular disease, diabetes, human immunodeficiency virus)
**Intervention**	All interventions directly and/or indirectly impacting nutrition (nutrition-specific and/or nutrition-sensitive)	
**Outcome**	At least one indicator for undernutrition and one for overnutrition:<br>• Undernutrition: stunting (height-for-age <-2 SD from the WHO Child Growth Standards median), underweight (weight-for-age <-2 SD), wasting (weight-for-height <- 2 SD), thinness (BMI-for-age <-2 SD), anaemia (low haemoglobin), low birth weight (<2500 grams), small-for-gestational age (birth weight <10th percentile for gestational age), height-for-age Z-score, haemoglobin concentration<br>• Overnutrition*: overweight (weight-for-height >2 SD from the WHO Child Growth Standards median in children younger than 5 years, BMI-for-age >1 SD for children aged 5–19 years, and BMI ≥25 and <30 kg/m2 in adults), obesity (weight-for-height >3 SD in children younger than 5 years, BMI-gor-age >2 SD in children aged 5–19 years, BMI ≥30 kg/m2 in adults), macrosomia (birthweight >4000 grams), large-for-gestational age (birth weight >90th percentile for gestational age)<br>• Change in continuous outcomes stratified by baseline nutritional status (underweight, normal weight, overweight): body mass index-for-age, body mass index, waist circumference, percentage body fat	Single-sided categorical outcomes, continuous outcomes that cannot be attributed to undernutrition or overnutrition (e.g. overall change in body mass index)
**Study Design**	Randomised controlled trials, cluster-randomised controlled trials, non-randomised trials, interrupted time series, repeated measure studies, controlled before-after studies, prospective cohort studies	Non-comparative studies, cross-sectional studies, uncontrolled before-after studies, case-control studies, retrospective cohort studies

Note: * Studies applying different definitions for overnutrition indicators were included nonetheless but this was noted during the data extraction.

Supplementary Table 2A. Studies not retrieved or excluded from database search, grouped by reason for exclusion.

**Full-text could not be retrieved (n=3)**
Burlandy (2010), [1] Sanchez (2000), [2] Okezie (2007) [3]
**Study protocol (n=6)**
Fenn (2015), [4] Gonzalez Acero (2020), [5] Kittisakmontri (2020), [6] Mahmudiono (2016), [7] Pesu (2021), [8] Sangalang (2021) [9]
**Comparator (n=3)**
Malpeli (2013), [10] Sekiyama (2017), [11] Varea (2011) [12]
**Wrong intervention (n=18)**
Al-Zeidaneen (2017), [13] Cheng (2019), [14] DeLacey (2021), [15] Dong (2019), [16] Faber (2015), [17] Gurung (2018), [18] Haschke (2017), [19] Ibrahim (2022), [20] Khonje (2020), [21] Lee (2021), [22] Locks (2017), [23] Po (2020), [24] Potdar (2014), [25] Santos (2018), [26] Silva (2021), [27] Tian (2016), [28] Zhang (2016), [29] Zheng (2020) [30]
**Wrong outcomes (n=178)**
Abreu (2021), [31] Abril (2009), [32] Adom (2010), [33] Agdeppa (2022), [34] Agrasada (2005), [35] Aguila (2023), [36] Ahmad (2020), [37] Akeredolu (2014), [38] Aldana-Parra (2020), [39] Andrade (2016), [40] Angeles-Agdeppa (2011), [41] Arsenault (2009), [42] Argaw (2023), [43] Arunambika (2021), [44] Ashorn (2015), [45] Assis (2015), [46] Augusto (2010), [47] Azimi (2020), [48] Barber (2008), [49] Barber (2010), [50] Barennes (2022), [51] Barth-Jaeggi (2015), [52] Beckmann (2022), [53] Behrman (2009), [54] Bisimwa (2012), [55] Bliss (2016), [56] Bliznashka, [57] Briaux (2020), [58] Brits (2017), [59] Brown (2007), [60] Brown (2010), [61] Callaghan-Gillespie (2017), [62] Cameron (2019), [63] Campbell (2016), [64] Cao (2013), [65] Carrasco Quintero (2013), [66] Carvalhaes (2005), [67] Chanani (2019), [68] Chen (2023), [69] Christian (2015), [70] Chowdhury (2022), [71] Colunga (2021), [72] Da Silva Rocha (2011), [73] Dawood (2019), [74] DeBoer (2022), [75] Deepthi (2014), [76] Delisle (2013), [77] Dennis (2019), [78] Dereje (2023), [79] Desalegn (2021), [80] Destaw (2022), [81] Dhaded (2020), [82] Dijkhuizen (2001), [83] Dijkhuizen (2009), [84] Ding (2023), [85] Ebrahimi (2006), [86] Echague (2019), [87] Ekoe (2020), [88] Engebretsen (2014), [89] Fabiansen (2018), [90] Fatima (2018), [91] Fenn (2021), [92] Fernald (2009), [93] Fianu (2016), [94] Filio (2023), [95] Finkelstein (2019), [96] Frith (2015), [97] Galasso (2019), [98] Ganmaa (2022), [99] Gibson (2003), [100] Gitau (2005), [101] Goyena (2018), [102] Hall (2007), [103] Harris (2011), [104] Heo (2020), [105] Hettiarachchi (2008), [106] Huda (2020), [107] Hung (2005), [108] Hurley (2021), [109] Hyder (2007), [110] Iannotti (2015), [111] Inayati (2012), [112] Kandpal (2016), [113] Khan (2021), [114] Kiplagat (2022), [115] Kowalski (2023), [116] Krahenbuhl 1998, [117] Kulwa (2023), [118] Labib (2019), [119] Labrecque (2018), [120] Langendorf (2014), [121] Lechtig (2009), [122] Lelijveld (2021), [123] Leroy (2021), [124] Li (2023), [125] Lind (2004), [126] Lind (2008), [127] Lisboa (2022), [128] Long (2020), [129] Longfils (2008), [130] Lucas (2022), [131] Mahdavi (2015), [132] Mahmudiono (2018), [133] Mamiro (2004), [134] Manary (2004), [135] Marquis (2015), [136] Marquis (2018), [137] Maselko (2015), [138] Matias (2017), [139] Mayhew (2014), [140] McLellan (2013), [141] Medeiros(2015), [142] Megally (2020), [143] Mehta (2022), [144] Miller (2020), [145] Mousa (2004), [146] Mridha (2016), [147] Muller (2003), [148] Muthayya (2012), [149] Mwale (2022), [150] Nabwera (2017), [151] Nahar (2009), [152] Nesamvuni (2005), [153] Nikiema (2017), [154] Obatolu (2006), [155] Ojha (2020), [156] Ouedraogo (2009), [157] Owais (2017), [158] Paes-Sousa (2011), [159] Parakh (2008), [160] Penny (2005), [161] Perera (2021), [162] Phu (2012), [163] Phuong Hong (2017), [164] Pickering (2015), [165] Prawirohartono (2011), [166] Prawirohartono (2013), [167] Quinones (2023), [168] Rahman (2008), [169] Rahman (2022), [170] Ramakrishnan (2003), [171] Rasmussen (2010), [172] Rockers (2016), [173] Rosado (2011), [174] Roux (2014), [175] Saville (2018), [176] Schroeder (2002), [177] Seneviratne (2021), [178] Shaheen (2006), [179] Siddiqua (2022), [180] Singh (2017), [181] Siswati (2022), [182] Smuts (2019), [183] Som (2021), [184] Somasse (2018), [185] Soni (2022), [186] Sreeparna Ghosh (2019), [187] Stewart (2009), [188] Su (2020), [189] Sunawang (2009), [190] Suprapto (2012), [191] Susiloretni (2021), [192] Tamana (2021), [193] Taneja (2022), [194] Teo (2021), [195] Thakur (2011), [196] Thakur (2012), [197] Tomedi (2012), [198] Tran Thuy (2009), [199] Vellakkal (2015), [200] Wang (2012), [201] Wang (2021), [202] Ward (2019), [203] Xie (2023), [204] Xu (2020), [205] Yeudall (2002), [206] Yue (2023), [207] Zhang (2010), [208]
**Wrong population (n=1)**
Anindo (2019) [209]
**Wrong study design (n=16)**
Ballout (2021), [210] Dabone (2011), [211] das Chagas (2013), [212] Egbi (2020), [213] Fall (2009), [214] Ford (2018), [215] Garcia-Guerra (2019), [216] Gurri (2015), [217] Kusum (2020), [218] Ravi (2017), [219] Sanchez-Bernal (2017), [220] Shaveta (2007), [221] Silveira (2011), [222] Sperandio (2017), [223] Vasquez-Garibay (2018), [224] Vinod (2012) [225]
**Total not retrieved (n=3)**<br>**Total excluded in full-text screening (n= 222)**

Supplementary Table 2B. Studies identified via other methods and excluded, grouped by reason for exclusion.

**Wrong outcomes (n=21)**
Bhandari (2004), [226] Christian (2003), [227] Cogswell (2003), [228] Cook (2023), [229] Cruz (2022), [230] Draper (2010), [231] De la Cruz (2021), [232] Friis (2004), [233] Gupta (2007), [234] Joshi (2013), [235] Kaul (2018), [236] Kouam (2018), [237] Osrin (2005), [238] Luzot (2023), [239] Penny (2005), [240] Ramakrishnan (2003), [241] Rivera (2004), [242] Robertson (2013), [243] Taylor (2007), [244] Vaidya (2008), [245] Zagré (2007) [246]
**Total (n=21)**

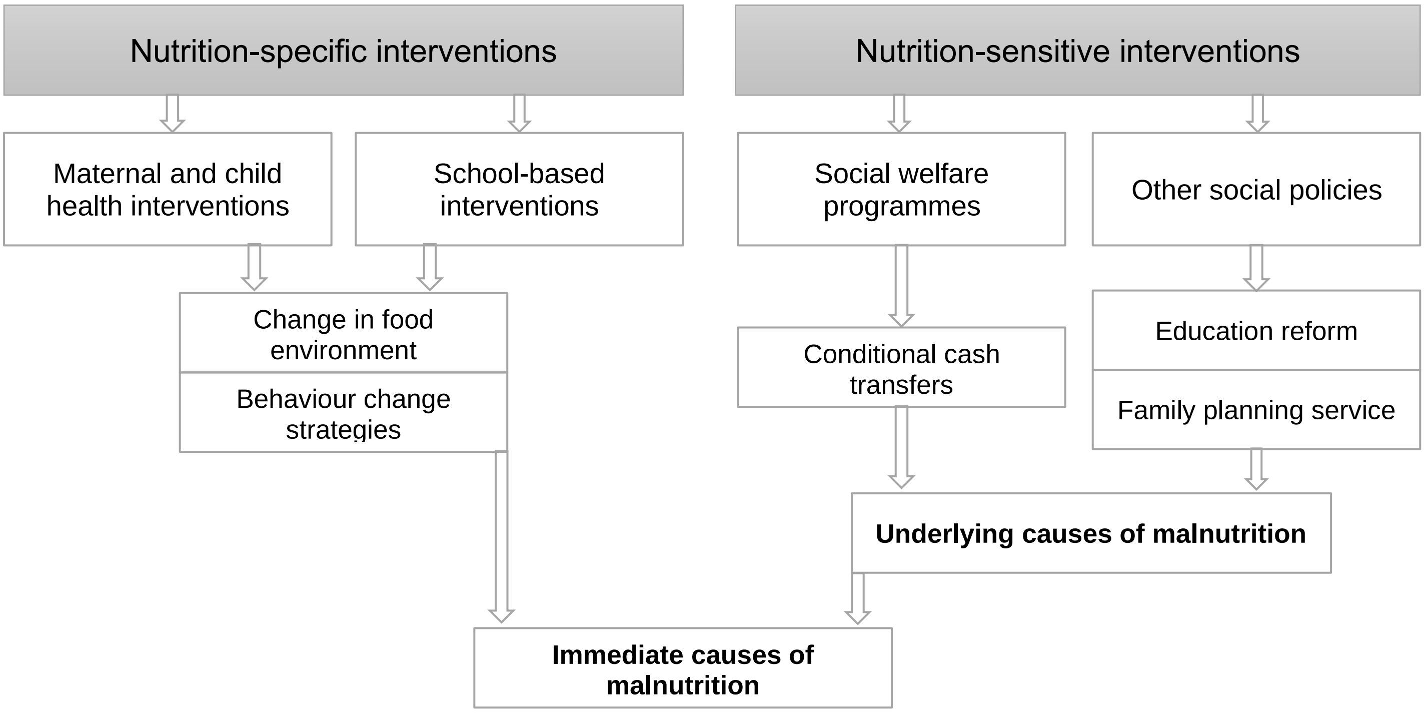

Supplementary Figure 1. Conceptual representation of types of interventions included in this review, adapted from a previous framework on nutrition actions. [247]

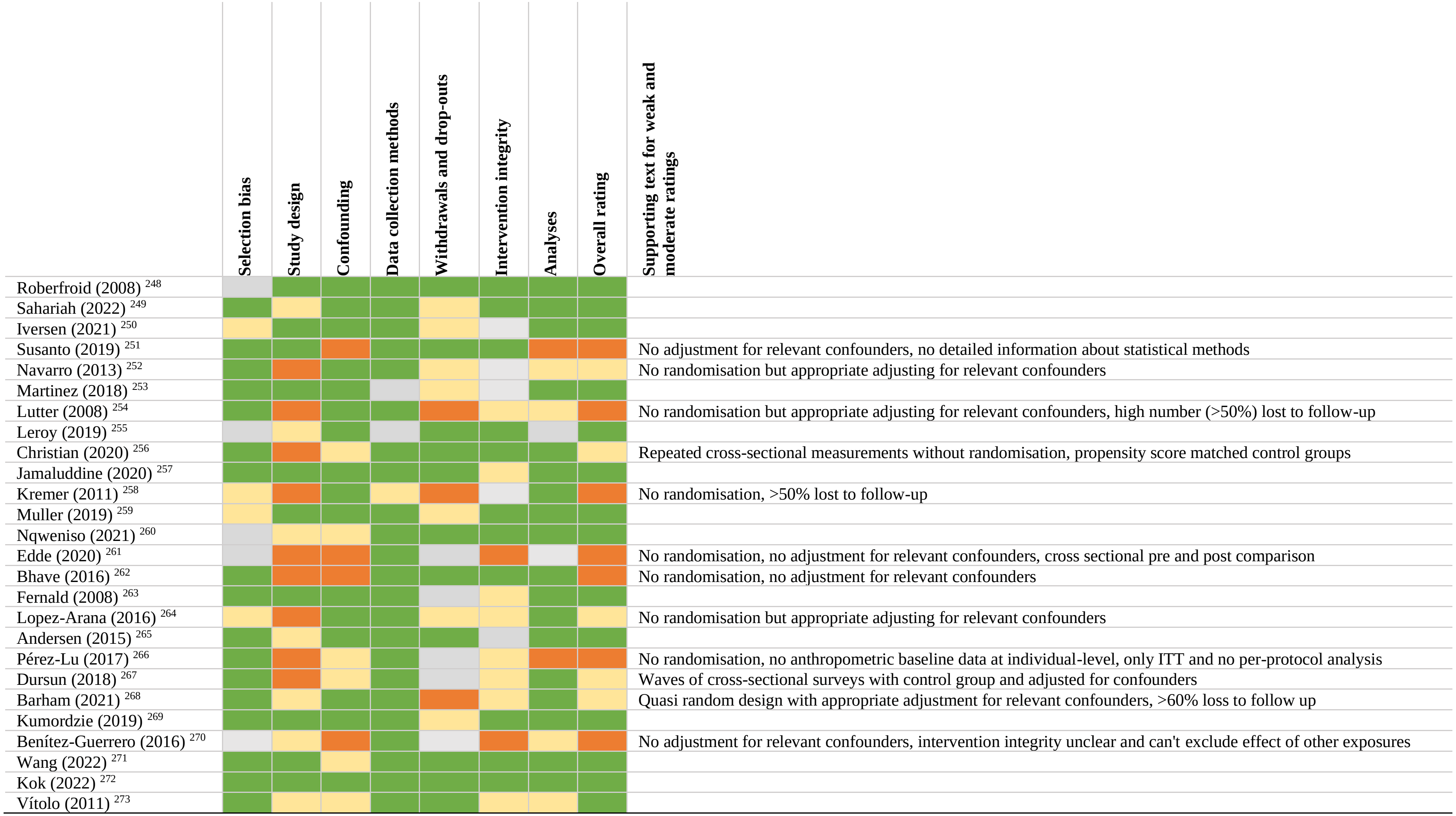

Supplementary Figure 2. Quality assessment for included studies (n=26).

Note: Green, yellow, and orange indicate strong, moderate, and weak rating, respectively. Grey indicates that not enough information was available for a judgement.

Abbreviations: ITT = intention-to-treat analysis.

Supplementary Table 3. Effect estimates and 95% CI for categorical outcomes.

Study	Sample size	Stunting	Thinness	Wasting	Underweight	Anaemic	Overweight	Obese	Effect measure
Fernald (2008) [263]	Total: 2449	**0·90 (0·85; 0·95)**	··	··	··	··	**0·92 (0·88; 0·97)**	··	OR
Lopez-Arana (2016) [264]	Total: 2874	0·92 (0·82; 1·05)	**0·25 (0·09; 0·74)**	··	··	··	1·30 (0·83; 2·03)	0·56 (0·20; 1·53)	OR
	Males: 1441	0·93 (0·80; 1·08)	0·48 (0·13; 1·80)	··	··	··	1·29 (0·73; 2·29)	0·42 (0·11; 1·56)	OR
	Females: 1433	0·93 (0·77; 1·12)	**0·19 (0·04; 0·81)**	··	··	··	1·17 (0·79; 1·76)	0·87 (0·36; 2·12)	OR
	Low maternal education: 2332	0·96 (0·83; 1·11)	**0·22 (0·07; 0·71)**	··	··	··	1·12 (0·75; 1·67)	0·59 (0·22; 1·60)	OR
	High maternal education: 542	**0·68 (0·52; 0·91)**	0·27 (0·01; 4·96)	··	··	··	1·53 (0·60; 3·96)	0·48 (0·11; 2·06)	OR
	Low household income: 1434	1·00 (0·86; 1·15)	0·68 (0·17; 2·65)	··	··	··	1·25 (0·74; 2·10)	0·49 (0·14; 1·63)	OR
	High household income: 1440	0·87 (0·72; 1·05)	**0·09 (0·02; 0·41)**	··	··	··	1·22 (0·68; 2·21)	0·76 (0·27; 2·13)	OR
Andersen (2015) [265]	Total: 338	-18·3 (-38·3; 1·59)	··	··	··	··	-8·9 (-24·7; 7·00)	··	DID
	Males: 190	-14·1 (-55·6; 27·4)	··	··	··	··	30·6 (-11·5; 72·6)	··	DID
	Females: 188	-19·0 (-38·5; 0·41)	··	··	··	··	**-22·6 (-42·5; -2·74)**	··	DID
Pérez-Lu (2017) [266]	Mothers: 5143	··	··	··	**0·39 (0·18; 0·85)**	0·89 (0·79; 1·00)	1·06 (0·98; 1·15)	··	PR
	Children: 5083	··	··	1·19 (0·57; 2·46)	··	**0·93 (0·86; 1·00)**	··	··	PR
Dursun (2018) [267]	Males: 5708	··	··	··	0·99 (0·97; 1·00)	··	1·03 (0·99; 1·07)	**1·04 (1·01; 1·06)**	OR
	Females: 6993	··	··	··	**0·97 (0·94; 0·99)**	··	0·96 (0·92; 1·01)	1·00 (0·97; 1·02)	OR
Barham (2021) [268]	4-15 y at enrollment: 524	··	··	··	-0·03 (-0·12; 0·05)	··	0·01 (-0·07, 0·09)	0·00 (-0·03; 0·04)	AD (proportion)
	16-27 y at enrollment: 679	··	··	··	-0·03 (-0·10; 0·04)	··	0·07 (0·00; 0·15)	0·01 (-0·03, 0·06)	AD (proportion)
	28-39 y at enrollment: 547	··	··	··	-0·04 (-0·11; 0·02)	··	0·04 (-0·05; 0·13)	-0·01 (-0·06; 0·05)	AD (proportion)
Roberfroid (2008) [248]	Total: 1052	SGA: 0·83 (0·65; 1·07)	··	··	LBW: 0·84 (0·58; 1·20)	··	LGA: **1·58 (1·04; 2·38)**	··	OR
Sahariah (2022) [249]	Males: 552	··	··	1·04 (0·73, 1·49)	··	··	1·31 (0·55; 3·15)	··	OR
	Females: 463	··	··	0·76 (0·52, 1·11)	··	··	3·15 (0·63; 15·77)	··	OR
Iversen (2021) [250]	Total: 307	0·95 (0·52; 1·74)	··	NA	··	··	0·52 (0·23; 1·15)	··	OR
Susanto (2019) [251]	Total: 144	**0·47 (0·23; 0·99)**	··	**0·26 (0·09; 0·76)** [b]	1·00 (0·31; 3·26)	··	0·81 (0·32; 2·01) [b]	··	OR
Navarro (2013) [252]	Total: 452	0·50 (0·22; 1·10)	··	··	··	··	**0·43 (0·23; 0·77)**	··	OR
Martinez (2018) [253]	Stunting: 1540<br>Wasting: 1516<br>Anaemia: 1491<br>Overweight: 1511	1·01 (0·96; 1·10)	··	1·00 (0·99; 1·01)	··	0·97 (0·92; 1·02)	1·00 (0·98; 1·03)	··	OR
Lutter (2008) [254]	Anthropometry: 319<br>Anaemia: 154	NS	··	NS	0·75 (NP; NP)	**0·58 (0·24; 0·75)**	p=0·843	··	OR

Leroy (2019) [255]	Total: 1191	··	··	··	0·47 (0·15; 1·52) [b]	··	1·22 (0·94; 1·58) [b]	1·27 (0·78; 2·08) [b]	OR [b]
Christian (2020) [256]	Total: 2419	-2·6 (-10·15; 4·95)	··	**-2·6 (-4·58; -0·62)**	··	-9·5 (-26·35; 7·35)	5·6 (-0·87; 12·07)	··	DID
Jamaluddine (2020) [257]	Total: 1102	1·47 (0·73; 2·97)	NA	··	··	0·88 (0·76; 1·01)	0·98 (0·65; 1·49)	1·06 (0·48; 2·30)	OR
Kremer (2011) [258]	Total: 2948	··	Unadjusted difference: 0·11 (NP; NP)	··	··	··	0·34 (-0·03; 0·71)	··	AD (proportion)
	Indigenous Fijian: 956	··	NP	··	··	··	0·38 (-0·14; 0·90)	··	AD (proportion)
	Indo-Fijian and other: 1992	··	NP	··	··	··	0·32 (-0·19; 0·83)	··	AD (proportion)
Muller (2019) [259]	Total: 519	0·76 (0·09; 6·53)	··	··	··	0·93 (0·38; 2·30)	**0·21 (0·07; 0·66)** [b]	··	OR
Nqweniso (2021) [260]	Total: 255	··	··	··	Mean difference (unadjusted) of BMIZ in underweight subjects before and after intervention is larger in all intervention groups compared to control groups but no estimates for intervention's effect is shown. Same for %FM, apart from Arm 4, where %FM decreased in underweight subjects of intervention group.	··	BMIZ in initially overweight subjects: [a]<br>Arm 1: 0·20 (-0·01; 0·41)<br>Arm 2: -0·10 (-0·29; 0·10)<br>Arm 3: -0·20 (-0·44; 0·04)<br>Arm 4: NA<br><br>Body fat (%) in initially overweight subjects: [a]<br>**Arm 1: 10·09 (5·93; 14·30)**<br>Arm 2: 0·80 (-1·99; 3.59)<br>Arm 3: -0·62 (-6·31; 5·07)<br>Arm 4: NA	··	AD [a]
Edde (2020) [261]	Total: 653	0·70 (0·36; 1·37)	0·79 (0·44; 1·42)	··	··	**0·44 (0·30; 0·65)**	0·63 (0·19; 2·11)	··	OR
Bhave (2016) [262]	Total: 491	··	··	··	0·85 (0·43; 1·70) [b]	··	0·92 (0·61; 1·37) [b]	··	OR [b]
	Males: 252	··	··	··	1·04 (0·44; 2·43) [b]	··	0·78 (0·46; 1·32) [b]	··	OR [b]
	Females: 239	··	··	··	0·69 (0·21; 2·25) [b]	··	1·36 (0·7; 2·66) [b]	··	OR [b]
Kumordzie (2019) [269]	Total: 960	1·00 (0·58, 1·73)	··	··	1·10 (0·63, 1·93)	··	1·10 (0·47, 2·54)	··	OR
Benítez-Guerrero (2016) [270]	Total: 368	··	··	··	0·81 (0·51; 1·29) [b]	··	0·97 (0·61; 1·55) [b]	0·97 (0·57; 1·66) [b]	OR [b]

Wang (2022) [271]	Total: 7783	SGA: **0·77 (0·68; 0·87)** [b]	··	··	LBW: **0·80 (0·66; 0·96)** [b]	··	LGA:1·02 (0·89; 1·17) [b]	··	OR [b]
Kok (2022) [272]	Total: 1708	−2·93 (−7·04, 1·17)	··	··	**−4·07 (−6·86, −1·28)**	··	0·20 (−1·01, 1·40)	··	AD (%)
Vítolo (2011) [273]	Total: 307	··	··	··	0·5 (0·23; 1·07) [b]		0·91 (0·58; 1·43) [b]		OR [b]

Note: a: Estimate is for control group and participation in intervention is reference group. b: No effect estimates provided in paper and unadjusted OR was recalculated by review authors (OR = odds of event in exposed group / odds of event in non-exposed group). ***Bold***: denotes statistical significance (p<0·05) based on p-values reported in the original studies. *Abbreviations:* AD = adjusted difference (proportion). OR = Odds ratio. PR = Prevalence ratio. DID = Difference-in-difference estimate (%). NA = not applicable as number of cases too low to calculate estimate. NS = not significant but no effect estimate provided in paper. NP = not provided and could not be retrieved from authors. LBW = low birthweight. SGA = small-for-gestational age. LGA = large-for-gestational age.

Supplementary Table 4. Effect estimates and 95% CI for continuous outcomes.

Study	Sample size	WAZ	WHZ	BMIZ	HAZ	Haemoglobin	BMI	Height (cm)	Weight (kg)	Others	Effect measure
Fernald (2008) [263]	Total: 2449	··	··	··	**0·20 (0·09; 0·30)**	0·90 g/L (-0·56; 2·37)	··	··	··	**BMI for age percentile: -2·85 (-5·54; -0·15)**	AD
Lopez-Arana (2016) [264]	Total: 2874	··	··	**0·14 (0·00; 0·27)**	0·00 (−0·10; 0·11)	··	··	··	··	··	DID
	Males: 1441	··	··	0·12 (-0·09; 0·33)	-0·01 (-0·08; 0·05)	··	··	··	··	··	DID
	Females: 1433	··	··	0·14 (-0·01; 0·30)	0·02 (-0·18; 0·23)	··	··	··	··	··	DID
	Low maternal education: 2332	··	··	0·08 (-0·07; 0·22)	-0·01 (-0·08; 0·06)	··	··	··	··	··	DID
	High maternal education: 542	··	··	0·32 (-0·09; 0·72)	0·02 (-0·11; 0·15)	··	··	··	··	··	DID
	Low household income: 1434	··	··	0·10 (-0·05; 0·24)	0·04 (-0·01; 0·09)	··	··	··	··	··	DID
	High household income: 1440	··	··	0·17 (-0·03; 0·37)	-0·04 (-0·20; 0·11)	··	··	··	··	··	DID
Andersen (2015) [265]	Total: 338	··	··	-0·36 (-0·79; 0·06)	0·14 (-0·20; 0·49)	··	··	··	··	··	DID
	Males: 190	··	··	-0·34 (-0·56; 0·49)	**0·43 (0·09; 0·77)**	··	··	··	··	··	DID
	Females: 188	··	··	**-0·60 (-1·0; -0·21)**	-0·19 (-0·79; 0·41)	··	··	··	··	··	DID
Dursun (2018) [267]	Males: 5708	··	··	··	··	··	**0·47 (0·22; 0·72)**	··	··	··	Reduced form effect estimate
	Females: 6993	··	··	··	··	··	-0·05 (-0·43; 0·33)	··	··	··	Reduced form effect estimate
Barham (2021) [268]	4-15 y at enrollment: 524	··	··	··	··	··	0·27 (-0·43; 0·97)	··	··	··	AD
	16-27 y at enrollment: 679	··	··	··	··	··	0·57 (-0·04; 1·18)	··	··	··	AD
	28-39 y at enrollment: 547	··	··	··	··	··	0·28 (-0·38; 0·94)	··	··	··	AD
Roberfroid (2008) [248]	Total: 1052	··	··	··	··	-0·19 g/L (-0·53; 0·14)	··	**3·60 (0·80; 6·33)**	**0·05 (0·00; 0·10)**	··	AD
Sahariah (2022) [249]	Total: 1015	··	··	0·23	0·91	··	NP	··	··	%FM: p=0·39	P-value from t-test
	Males: 552	··	··	1·00 (0·98; 1·01)	NP	··	1·00 (0·98; 1·01)	··	··	%FM: 0·98 (0·92; 1·03)	DID
	Females: 463	··	··	**0·03 (0·00; 0·05)**	NP	··	**1·02 (1·01; 1·04)**	··	··	%FM: **1·07 (1·01; 1·13)**	DID
Navarro (2013) [252]	Total: 452	··	··	**-0·31 (-0·49; -0·12)**	0·21 (-0·02; 0·44)	··	··	··	··	··	AD

Martinez (2018) [253]	HAZ: 1538<br>WAZ: 1513<br>WHZ: 1514<br>BMI: 1511	-0·06 (-0·15; 0·03)	-0·04 (-0·14; 0·06)	-0·03 (-0·13; 0·07)	-0·05 (-0·15; 0·05)	··	··	··	··	··	AD
Lutter (2008) [254]	Total: 319	··	··	··	··	··	··	0·37 (-0·17; 0·86)	NP	··	AD
	Older children (12 - 14 mo at baseline): 130	··	··	··	··	··	··	0·66 (-0·80; 1·41)	**-3·90 (-7·67; -0·12)**	··	AD
Leroy (2019) [255]	Total: 1191	··	··	··	··	··	··	··	0·54 (0·00; 1·07)	··	AD
Christian (2020) [256]	Total: 2419	··	**0·21 (0·05; 0·36)**	**0·23 (0·08; 0·38)**	0·01 (-0·21; 0·23)	0·10 g/dL (-0·45; 0·65)	··	0·04 (-0·37; 0·45)	0·13 (-0·01; 0·26)	··	DID
Jamaluddine (2020) [257]	Total: 1102	··	··	0·04 (-0·14; 0·19)	-0·01 (-0·10; 0·08)	**3·26 g/L (0·99; 5·54)**	··	··	··	··	AD
Kremer (2011) [258]	Total: 2948	··	··	0·02 (-0·02; 0·07)	··	··	0·10 (-0·03; 0·23)	··	0·05 (-0·37; 0·48)	%FM: **-1·17 (-1·73; -0·60)**	AD
	Indigenous Fijian: 956	··	··	0·03 (-0·03; 0·09)	··	··	0·02 (-0·22; 0·26)	··	0·05 (-0·81; 0·91)	%FM: **-0·93 (-1·53; -0·32)**	AD
	Indo-Fijian and other: 1992	··	··	0·02 (-0·03; 0·07)	··	··	0·12 (-0·01; 0·24)	··	0·05 (-0·40; 0·49)	%FM: **-1·26 (-1·90; -0·61)**	AD
Muller (2019) [259]	Total: 519	··	··	**-0·17 (-0·24; -0·09)**	··	··	··	··	··	**Skinfolds: -1·06 (-1·83; -0·29)**	AD
Nqweniso (2021) [260]	Total: 255	··	··	Arm 1: 0·03 (-0·14; 0·20)<br>Arm 2: -0·04 (-0·16; 0·08)<br>Arm 3: -0·09 (-0·19; 0·01)<br>**Arm 4: -0·24 (-0·33; -0·16)**	··	··	··	··	··	%FM:<br>**Arm 1: 2·35 (1·34; 3·36)**<br>**Arm 2: 0·83 (0·09; 1·58)**<br>Arm 3: -0·63 (-1·44; 0·18)<br>Arm 4: 0·53 (-0·35; 1·40)	AD [a]
Bhave (2016) [262]	Total: 491	··	··	p=0·46	··	··	p=0·50	**p<0·01**	··	**Waist circumference: p<0·01**	P-value from t-test
	Males: 252	··	··	p=0·18	··	··	p=0·49	**p<0·01**	··	**Waist circumference: p<0·01**	P-value from linear regression
	Females: 239	··	··	p=0·57	··	··	p=0·17	**p=0·02**	··	**Waist circumference: p<0·01**	P-value from linear regression
Kumordzie (2019) [269]	Total: 960	0·07 (−0·05, 0·18)	··	0·03 (−0·08, 0·14)	0·08 (−0·05, 0·21)	··	··	0·36 (−0·23, 0·95)	0·17 (−0·10, 0·43)	%FM: 0·16 (−0·49, 0·81), Fatfree mass: −0·29 (−1.02, 0·43)	AD

										MUAC: 0·07 (−0·09, 0·23)	
Benítez-Guerrero (2016) [270]	Total: 368	··	··	Females: p<0·001 males: p=0·232	··	··	··	··	··	··	p-value from t-test (pre-post intervention comparison)
Wang (2022) [271]	Total: 7783	··	··	··	··	-0·1 (-0·20; 0·00) [b]	··	0·1 (-0·15; 0·35) [b]	**64·5 (42·18; 86·82)** [b]	··	Mean difference (unadjusted) [b]
Kok (2022) [272]	Total: 1708	··	··	··	··	··	··	··	**0·20 (0·01, 0·39)**	**%FM: 49·7 (10·8, 88·7)**	Adjusted difference
Vítolo (2011) [273]	Baseline underweight: 27	··	··	··	··	··	··	··	p=0·8	··	p-value from t-test
	Baseline normal weight: 164	··	··	··	··	··	··	··	p=0·2	··	p-value from t-test
	Baseline overweight: 114	··	··	··	··	··	··	··	p=**0·01**	··	p-value from t-test

Note: a: Estimate is for control group and participation in intervention is reference in model. b: No effect estimates provided in paper and unadjusted mean difference and 95%CI was recalculated by review authors. ***Bold***: denotes statistical significance (p<0.05) based on p-values reported in the original studies. *Abbreviations:* AD = Adjusted difference. OR = Odds ratio. PR = Prevalence ratio. DID = Difference-in-difference estimate. WAZ = weight-for-age z-score. WHZ = weight-for-height z-score. HAZ = height-for-age z-score. BMIZ = body mass index-for-age z-score. NP = estimate not provided and could not be retrieved through contacting authors. FM = fat mass.

Supplementary Table 5. Covariates that were adjusted for in most adjusted model for each study.

Study	Model adjusted for
Fernald (2008) [263]	Age, sex, maternal age, maternal education, maternal height, maternal intelligence, household composition, father's education, number of people in the household, language of household head, presence of electricity and water, number of small and draft animals, asset index
Lopez-Arana (2016) [264]	Age, sex, participation in Hogares Comunitarios (home-based health care), mother's marital status, mother's age, mother's education, mother's BMI, household income, level of urbanisation, inhabitants, and region
Andersen (2015) [265]	Propensity score matching
Pérez-Lu et al (2017) [266]	Propensity score matching based on maternal, child and household-level characteristics
Dursun (2018) [267]	Clustering on region by year-of-birth, year of birth differentiated by exposure to the education reform status, survey year fixed effects, region of residence fixed effects, and region-specific birth year trends
Barham (2021) [268]	Clustering on village, baseline characteristics, attrition
Roberfroid (2008) [248]	Malaria prevention, health centre, and gestational age
Sahariah (2022) [249]	Maternal age, BMI, height, and parity, SES, and the child's birth weight and gestation
Iversen (2021) [250]	Multilevel mixed-effect model with a cluster as the random intercept
Susanto (2019) [251]	Unadjusted
Navarro (2013) [252]	Logistic model: Age, sex, maternal education, maternal height, maternal BMI; linear model: Age, sex, maternal work status, household wealth index, number of children in the home, maternal height, maternal BMI, and birthweight
Martinez (2018) [253]	Child age, child sex, child age x sex interactions, caregiver age in years, caregiver years of education, household size, monthly household per-capita income, dummy variables for water connection, sewerage connection and bathroom or latrine
Lutter (2008) [254]	Child age, child age squared, baseline length, the indices for socioeconomic level, housing, education, and employment of household head and the interaction term for child age at baseline and program
Leroy (2019) [255]	For categorical variables: unadjusted; Linear model: Number of household adult equivalents, household wealth, education level of household head and mother, mother's ability to speak Spanish, maternal age and height, health convergence centre (unit of randomisation) and wave x treatment interaction term
Christian (2020) [256]	Propensity score matching based on maternal, child and household-level characteristics
Jamaluddine (2020) [257]	Baseline measurement, age, sex, maternal education level, monthly expenditure per capita, crowding index, household food insecurity, and school clustering effect
Kremer (2011) [258]	Age at follow-up, gender, ethnic subgroup, height at follow-up, duration between measurements, clustering by school
Muller (2019) [259]	Age, sex, HAZ, haemoglobin, SES index, intestinal protozoa, and soil-transmitted helminth infection status at baseline
Nqweniso (2021) [260]	Categorical variables: unadjusted; linear model: SES, baseline values, age, and sex
Edde (2020) [261]	Year of study (2009 / 2014), school type (private, public) and location (urban, suburban), interactions terms between above mentioned variables and control / intervention
Bhave (2016) [262]	Sex
Kumordzie (2019) [269]	Child age, sex, maternal gestational age, nulliparity, maternal education, estimated pre-pregnancy BMI, maternal height, and asset score
Benítez-Guerrero (2016) [270]	Unadjusted
Wang (2022) [271]	Unadjusted
Kok (2022) [272]	Health centre and randomisation block as fixed effect to account for clustering by the study design. Adjusted models additionally contained a priori set known prognostic factors of birth outcome including maternal age, primiparity, gestational age, height, MUAC, BMI, and haemoglobin level at study enrolment.
Vítolo (2011) [273]	Unadjusted

Abbreviations: SES = Socioeconomic status. HAZ = height-for-age z-score. MUAC = mid-upper arm circumference. BMI = body mass index.

Appendix 4.2 Search strategy

Note: The initial search was conducted on March 17, 2022, followed by an updated search on August 14, 2023. The search strategy remained consistent, with the only difference being that the second search included not only English articles but also articles in Portuguese, Spanish and French. Additionally, the search filters for case-control studies were removed in the second search, as these were deemed ineligible study designs for this review. The below shows the revised strategies from August 14, 2023. Published search strategies were used to filter for study designs. [274,275]

Appendix 4.2.1 Search strategy for Ovid MEDLINE.

1.	((food or nutrition* or diet* or eating or sugar* or beverage* or drink* or fat or fats or fatty or fruit* or vegetable* or meal* or menu*) and (program* or intervention* or policy or policies or action* or plan* or law or laws or legislation or strategy or tax* or subsid* or voucher* or coupon* or educat* or campaign* or fortif* or scheme* or reformulat* or guideline* or standard* or market* or label* or packag* or advert* or transfer* or basket or supplement*)).mp.
2.	((Breastfeed* or "breast feed*" or "complementary feed*" or "child* feeding" or "infant feed*") and (protect* or support* or promot* or improv* or empower* or service* or provi*)).mp.
3.	((wom?n* or female* or "maternal health" or "maternal schooling" or "maternal education" or "family plan*" or "drink* water" or sanitation or hygiene* or child) and (protect* or support* or promot* or improv* or empower* or service* or provi* or program*)).mp.
4.	("conditional cash transfer*" or "social welfare" or "social safety net*" or "trad* agreement" or "foreign direct invest*" or "trad* tarif*" or "capacity investment*" or "domestic resource mobili?ation" or (agricultur* and (program* or intervention* or policy or policies or action* or plan* or law or laws or legislation or strategy)) or "leadership program*").mp.
5.	exp nutrition policy/ or fiscal policy/ or exp international health regulations/ or exp Legislation, Food/ or exp health promotion/ or consumer health information/ or Food Assistance/ or exp Food Supply/ or exp Food Services/ or Early Intervention, Educational/
6.	1 or 2 or 3 or 4 or 5
7.	(undernutrition or underweight or "low weight" or stunt* or "height for age" or "length for age" or "stature for age" or (short and (height or stature)) or (growth and (disorder or impair* or retard or delay*)) or wast* or "low weight for height" or thin* or "low BMI" or "low body mass index" or "iron deficienc*" or ane?mi* or "low birth weight" or LBW or "small for gestational age").mp.
8.	exp malnutrition/ or thinness/ or wasting syndrome/ or exp Growth Disorders/ or exp anemia/ or exp weight loss/
9.	7 or 8
10.	(overnutrition or overweight or "over weight" or obes* or adiposity* or ((excessive or high or abnormal) and (bodyweight or weight)) or "high weight for height" or "high bmi for age" or "high waist to hip ratio" or "high waist circumference" or "high waist to stature ratio" or "high waist to height ratio" or "macrosomia" or "large for gestational age" or "high birth weight").mp.
11.	overweight/ or exp obesity/ or overnutrition/ or weight gain/
12.	10 or 11
13.	9 and 12
14.	Afghanistan/ or Angola/ or Albania/ or Argentina/ or Armenia/ or "American Samoa"/ or Azerbaijan/ or Burundi/ or Benin/ or "Burkina Faso"/ or Bangladesh/ or Bulgaria/ or "Bosnia and Herzegovina"/ or Belarus/ or Belize/ or Bolivia/ or Brazil/ or Bhutan/ or Botswana/ or "Central African Republic"/ or China/ or "Côte d'Ivoire "/ or Cameroon/ or Congo/ or "Democratic Republic of Congo"/ or Colombia/ or Comoros/ or "Cabo Verde"/ or "Costa Rica"/ or Cuba/ or Djibouti/ or Dominica/ or "Dominican Republic"/ or Algeria/ or Ecuador/ or Egypt/ or Eritrea/ or Ethiopia/ or Fiji/ or Micronesia/ or Gabon/ or Georgia/ or Ghana/ or Guinea/ or Gambia/ or "Guinea-Bissau"/ or "Equatorial Guinea"/ or Grenada/ or Guatemala/ or Guyana/ or Honduras/ or Haiti/ or Indonesia/ or India/ or Iran/ or Iraq/ or Jamaica/ or Jordan/ or Kazakhstan/ or Kenya/ or "Kyrgyz Republic"/ or Cambodia/ or Kiribati/ or Lao People's Democratic Republic/ or Lebanon/ or Liberia/ or Libya/ or "Saint Lucia"/ or "Sri Lanka"/ or Lesotho/ or Morocco/ or Moldova/ or Madagascar/ or Maldives/ or Mexico/ or "Marshall Islands"/ or "North Macedonia"/ or Mali/ or Myanmar/ or Montenegro/ or Mongolia/ or Mozambique/ or Mauritania/ or Mauritius/ or Malawi/ or Malaysia/ or Namibia/ or Niger/ or Nigeria/ or Nicaragua/ or Nepal/ or Pakistan/ or Panama/ or Peru/ or Philippines/ or "Papua New Guinea"/ or "Democratic People's Republic of Korea"/ or Paraguay/ or "West Bank and Gaza"/ or Romania/ or "Russian Federation"/ or Russia/ or Rwanda/ or Sudan/ or Senegal/ or "Solomon Islands"/ or "Sierra Leone"/ or "El Salvador"/ or Somalia/ or Serbia/ or "South Sudan"/ or "Sao Tome and Principe"/ or Suriname/ or Eswatini/ or "Syrian Arab Republic"/ or Chad/ or Togo/ or Thailand/ or Tajikistan/ or Turkmenistan/ or "Timor-Leste"/ or Tonga/ or Tunisia/ or Turkey/ or Tuvalu/ or Tanzania/ or Uganda/ or Ukraine/ or Uzbekistan/ or "Saint Vincent and the Grenadines"/ or Vietnam/ or Vanuatu/ or Samoa/ or Kosovo/ or Yemen/ or "South Africa"/ or Zambia/ or Zimbabwe/
15.	(Afghanistan or Angola or Albania or Argentina or argentine or Armenia or "American Samoa" or Azerbaijan or Burundi or Benin or "Burkina Faso" or Bangladesh or Bulgaria or "Bosnia and Herzegovina" or Belarus or Belize or Bolivia or Brazil or brasil or Bhutan or Botswana or "Central African Republic" or China or "Cote d'Ivoire" or "ivory coast" or Cameroon or Congo or "Democratic Republic of Congo" or Colombia or Comoros or "Cabo Verde" or "Costa Rica" or Cuba or Djibouti or Dominica or "Dominican Republic" or Algeria or Ecuador or Egypt or Eritrea or Ethiopia or Fiji or Micronesia or Gabon or Georgia or Ghana or Guinea or Gambia or "Guinea-Bissau" or "Equatorial Guinea" or Grenada or Guatemala or Guyana or Honduras or Haiti or Indonesia or India or Iran or Iraq or Jamaica or Jordan or Kazakhstan or Kenya or "Kyrgyz Republic" or Cambodia or Kiribati or Lao or laos or Lebanon or Liberia or Libya or "St. Lucia" or "saint lucia" or "Sri Lanka" or Lesotho or Morocco or Moldova or Madagascar or Maldives or Mexico or "Marshall Islands" or "North Macedonia" or Mali or Myanmar or Montenegro or Mongolia or Mozambique or Mauritania or Mauritius or Malawi or Malaysia or Namibia or Niger or Nigeria or Nicaragua or Nepal or Pakistan or Panama or Peru or Philippines or "Papua New Guinea" or "Democratic People's Republic of Korea" or Paraguay or "West Bank and Gaza" or Romania or "Russian Federation" or russia or Rwanda or Sudan or Senegal or "Solomon Islands" or "Sierra Leone" or "El Salvador" or Somalia or Serbia or "South Sudan" or "Sao Tome and Principe" or Suriname or Eswatini or "Syrian Arab Republic" or Chad or Togo or Thailand or Tajikistan or Turkmenistan or "Timor Leste" or Tonga or Tunisia or Turkey or Tuvalu or Tanzania or Uganda

	or Ukraine or Uzbekistan or "St. Vincent and the Grenadines" or "saint vincent and the grenadines" or Vietnam or Vanuatu or Samoa or Kosovo or Yemen or " South Africa" or Zambia or Zimbabwe).mp.
16.	14 or 15
17.	6 and 13 and 16
18.	"randomized controlled trial".pt.
19.	(random$ or placebo$ or single blind$ or double blind$ or triple blind$).ti,ab.
20.	(random sampl$ or random digit$ or random effect$ or random survey or random regression).ti,ab. not "randomized controlled trial".pt.
21.	18 or 19
22.	21 not 20
23.	exp cohort studies/
24.	cohort$.tw.
25.	controlled clinical trial.pt.
26.	(((nonequivalent or non equivalent) adj3 control$) or posttest$ or post test$ or pre test$ or pretest$ or quasi experiment$ or quasiexperiment$ or timeseries or time series).tw.
27.	(pretest-posttest study or pretesting or pre-post tests quasi experiment* or repeat* measure* or time series or nonequivalent group* or regression discontinuity or propensity score or synthetic control method or difference-in-difference or natural experiment*).ti,ab,kw.
28.	22 or 23 or 24 or 25 or 26 or 27
29.	17 and 28
30.	exp animals/ not humans.sh.
31.	29 not 30
32.	limit 31 to yr="2000 -Current"
33.	(comment or editorial or "conference abstract" or "meeting abstract" or "meta-analysis" or review or "systematic review").pt.
34.	32 not 33
35.	limit 34 to english
36.	limit 34 to french
37.	limit 34 to spanish
38.	limit 34 to portuguese
39.	35 or 36 or 37 or 38

Appendix 4.2.2 Search strategy for Ovid Embase.

1.	((food or nutrition* or diet* or eating or sugar* or beverage* or drink* or fat or fats or fatty or fruit* or vegetable* or meal* or menu*) and (program* or intervention* or policy or policies or action* or plan* or law or laws or legislation or strategy or tax* or subsid* or voucher* or coupon* or educat* or campaign* or fortif* or scheme* or reformulat* or guideline* or standard* or market* or label* or packag* or advert* or transfer* or basket or supplement*)).mp.
2.	((Breastfeed* or "breast feed*" or "complementary feed*" or "child* feeding" or "infant feed*") and (protect* or support* or promot* or improv* or empower* or service* or provi*)).mp.
3.	((wom?n* or female* or "maternal health" or "maternal schooling" or "maternal education" or "family plan*" or "drink* water" or sanitation or hygiene* or child) and (protect* or support* or promot* or improv* or empower* or service* or provi* or program*)).mp.
4.	("conditional cash transfer*" or "social welfare program*" or "social safety net*" or "trad* agreement" or "foreign direct invest*" or "trad* tarif*" or "capacity investment*" or "domestic resource mobili?ation" or (agricultur* and (program* or intervention* or policy or policies or action* or plan* or law or laws or legislation or strategy)) or "leadership program*").mp.
5.	exp nutrition policy/ or food packaging/ or exp food assistance/ or consumer health information/ or exp health program/ or exp health promotion/ or exp health education/ or exp public health campaign/ or international health regulation/ or health legislation/ or fiscal policy/ or exp food legislation/ or nutrition labeling/ or nutrition service/ or nutritional facts label/ or nutritional facts labelling/
6.	1 or 2 or 3 or 4 or 5
7.	(undernutrition or underweight or "low weight" or stunt* or "height for age" or "length for age" or "stature for age" or (short and (height or stature)) or (growth and (disorder or impair* or retard or delay*)) or wast* or "low weight for height" or thin* or "low BMI" or "low body mass index" or "iron deficienc*" or ane?mi* or "low birth weight" or LBW or "small for gestational age").mp.
8.	malnutrition/ or exp stunting/ or exp stunting syndrome/ or exp growth retardation/ or exp short stature/ or chronic wasting disease/ or wasting syndrome/ or underweight/ or exp body weight loss/
9.	7 or 8
10.	(overnutrition or overweight or "over weight" or obes* or adiposity* or ((excessive or high or abnormal) and (bodyweight or weight)) or "high weight for height" or "high bmi for age" or "high waist to hip ratio" or "high waist circumference" or "high waist to stature ratio" or "high waist to height ratio" or macrosomia or "high birth weight" or "large for gestational age").mp.
11.	exp overnutrition/ or exp body weight gain/
12.	10 or 11
13.	Afghanistan/ or Angola/ or Albania/ or Argentina/ or Armenia/ or "American Samoa"/ or Azerbaijan/ or Burundi/ or Benin/ or "Burkina Faso"/ or Bangladesh/ or Bulgaria/ or "Bosnia and Herzegovina"/ or Belarus/ or Belize/ or Bolivia/ or Brazil/ or Bhutan/ or Botswana/ or "Central African Republic"/ or China/ or "Côte d'Ivoire "/ or Cameroon/ or Congo/ or "Democratic Republic of Congo"/ or Colombia/ or Comoros/ or "Cabo Verde"/ or "Costa Rica"/ or Cuba/ or Djibouti/ or Dominica/ or "Dominican Republic"/ or Algeria/ or Ecuador/ or Egypt/ or Eritrea/ or Ethiopia/ or Fiji/ or Micronesia/ or Gabon/ or Georgia/ or Ghana/ or Guinea/ or Gambia/ or "Guinea-Bissau"/ or "Equatorial Guinea"/ or Grenada/ or Guatemala/ or Guyana/ or Honduras/ or Haiti/ or Indonesia/ or India/ or Iran/ or Iraq/ or Jamaica/ or Jordan/ or Kazakhstan/ or Kenya/ or

	"Kyrgyz Republic"/ or Cambodia/ or Kiribati/ or Lao People's Democratic Republic/ or Lebanon/ or Liberia/ or Libya/ or "Saint Lucia"/ or "Sri Lanka"/ or Lesotho/ or Morocco/ or Moldova/ or Madagascar/ or Maldives/ or Mexico/ or "Marshall Islands"/ or "North Macedonia"/ or Mali/ or Myanmar/ or Montenegro/ or Mongolia/ or Mozambique/ or Mauritania/ or Mauritius/ or Malawi/ or Malaysia/ or Namibia/ or Niger/ or Nigeria/ or Nicaragua/ or Nepal/ or Pakistan/ or Panama/ or Peru/ or Philippines/ or "Papua New Guinea"/ or "Democratic People's Republic of Korea"/ or Paraguay/ or "West Bank and Gaza"/ or Romania/ or "Russian Federation"/ or Russia/ or Rwanda/ or Sudan/ or Senegal/ or "Solomon Islands"/ or "Sierra Leone"/ or "El Salvador"/ or Somalia/ or Serbia/ or "South Sudan"/ or "Sao Tome and Principe"/ or Suriname/ or Eswatini/ or "Syrian Arab Republic"/ or Chad/ or Togo/ or Thailand/ or Tajikistan/ or Turkmenistan/ or "Timor-Leste"/ or Tonga/ or Tunisia/ or Turkey/ or Tuvalu/ or Tanzania/ or Uganda/ or Ukraine/ or Uzbekistan/ or "Saint Vincent and the Grenadines"/ or Vietnam/ or Vanuatu/ or Samoa/ or Kosovo/ or Yemen/ or "South Africa"/ or Zambia/ or Zimbabwe/
14.	(Afghanistan or Angola or Albania or Argentina or argentine or Armenia or "American Samoa" or Azerbaijan or Burundi or Benin or "Burkina Faso" or Bangladesh or Bulgaria or "Bosnia and Herzegovina" or Belarus or Belize or Bolivia or Brazil or brasil or Bhutan or Botswana or "Central African Republic" or China or "Cote d'Ivoire" or "ivory coast" or Cameroon or Congo or "Democratic Republic of Congo" or Colombia or Comoros or "Cabo Verde" or "Costa Rica" or Cuba or Djibouti or Dominica or "Dominican Republic" or Algeria or Ecuador or Egypt or Eritrea or Ethiopia or Fiji or Micronesia or Gabon or Georgia or Ghana or Guinea or Gambia or "Guinea-Bissau" or "Equatorial Guinea" or Grenada or Guatemala or Guyana or Honduras or Haiti or Indonesia or India or Iran or Iraq or Jamaica or Jordan or Kazakhstan or Kenya or "Kyrgyz Republic" or Cambodia or Kiribati or Lao or laos or Lebanon or Liberia or Libya or "St. Lucia" or "saint lucia" or "Sri Lanka" or Lesotho or Morocco or Moldova or Madagascar or Maldives or Mexico or "Marshall Islands" or "North Macedonia" or Mali or Myanmar or Montenegro or Mongolia or Mozambique or Mauritania or Mauritius or Malawi or Malaysia or Namibia or Niger or Nigeria or Nicaragua or Nepal or Pakistan or Panama or Peru or Philippines or "Papua New Guinea" or "Democratic People's Republic of Korea" or Paraguay or "West Bank and Gaza" or Romania or "Russian Federation" or russia or Rwanda or Sudan or Senegal or "Solomon Islands" or "Sierra Leone" or "El Salvador" or Somalia or Serbia or "South Sudan" or "Sao Tome and Principe" or Suriname or Eswatini or "Syrian Arab Republic" or Chad or Togo or Thailand or Tajikistan or Turkmenistan or "Timor Leste" or Tonga or Tunisia or Turkey or Tuvalu or Tanzania or Uganda or Ukraine or Uzbekistan or "St. Vincent and the Grenadines" or "saint vincent and the grenadines" or Vietnam or Vanuatu or Samoa or Kosovo or Yemen or " South Africa" or Zambia or Zimbabwe).mp.
15.	13 or 14
16.	6 and 9 and 12 and 15
17.	(random$ or placebo$ or single blind$ or double blind$ or triple blind$).ti,ab.
18.	(random sampl$ or random digit$ or random effect$ or random survey or random regression).ti,ab. not exp randomized controlled trial/
19.	17 not 18
20.	exp cohort analysis/
21.	exp longitudinal study/
22.	exp prospective study/
23.	cohort$.tw.
24.	20 or 21 or 22 or 23
25.	(nonequivalent control group or posttesting or pretesting or pretest posttest design or pretest posttest control group design or quasi experimental methods or quasi experimental study or time series or time series analysis).sh.
26.	(((nonequivalent or non equivalent) adj3 control$) or posttest$ or post test$ or pre test$ or pretest$ or quasi experiment$ or quasiexperiment$ or timeseries or time series).tw.
27.	(time series or nonequivalent group* or regression discontinuity or propensity score or synthetic control method or difference-in-difference or natural experiment*).ti,ab,kw.
28.	exp pretest posttest control group design/ or exp pretest posttest design/ or exp quasi experimental study/ or exp time series analysis/
29.	25 or 26 or 27 or 28
30.	19 or 24 or 29
31.	16 and 30
32.	(animal$ not human$).sh,hw.
33.	31 not 32
34.	limit 33 to yr="2000 -Current"
35.	(comment or editorial or "conference abstract" or "meeting abstract" or "meta-analysis" or review or "systematic review").pt.
36.	34 not 35
37.	limit 36 to english
38.	limit 36 to french
39.	limit 36 to spanish
40.	limit 36 to portuguese
41.	37 or 38 or 39 or 40

Appendix 4.2.3 Search strategy for Ovid Global Health.

1.	((food or nutrition* or diet* or eating or sugar* or beverage* or drink* or fat or fats or fatty or fruit* or vegetable* or meal* or menu*) and (program* or intervention* or policy or policies or action* or plan* or law or laws or legislation or strategy or tax* or subsid* or voucher* or coupon* or educat* or campaign* or fortif* or scheme* or reformulat* or guideline* or standard* or market* or label* or packag* or advert* or transfer* or basket or supplement*)).mp.
2.	((Breastfeed* or "breast feed*" or "complementary feed*" or "child* feeding" or "infant feed*") and (protect* or support* or promot* or improv* or empower* or service* or provi*)).mp.
3.	((wom?n* or female* or "maternal health" or "maternal schooling" or "maternal education" or "family plan*" or "drink* water" or sanitation or hygiene* or child) and (protect* or support* or promot* or improv* or empower* or service* or provi* or program*)).mp.

4.	("conditional cash transfer*" or "social welfare" or "social safety net*" or "trad* agreement" or "foreign direct invest*" or "trad* tarif*" or "capacity investment*" or "domestic resource mobili?ation" or (agricultur* and (program* or intervention* or policy or policies or action* or plan* or law or laws or legislation or strategy)) or "leadership program*").mp.
5.	1 or 2 or 3 or 4
6.	(undernutrition or underweight or "low weight" or stunt* or "height for age" or "length for age" or "stature for age" or (short and (height or stature)) or (growth and (disorder or impair* or retard or delay*)) or wast* or "low weight for height" or thin* or "low BMI" or "low body mass index" or "iron deficienc*" or ane?mi* or "low birth weight" or LBW or "small for gestational age").mp.
7.	(overnutrition or overweight or "over weight" or obes* or adiposity* or ((excessive or high or abnormal) and (bodyweight or weight)) or "high weight for height" or "high bmi for age" or "high waist to hip ratio" or "high waist circumference" or "high waist to stature ratio" or "high waist to height ratio" or "macrosomia" or "large for gestational age" or "high birth weight").mp.
8.	6 and 7
9.	(Afghanistan or Angola or Albania or Argentina or argentine or Armenia or "American Samoa" or Azerbaijan or Burundi or Benin or "Burkina Faso" or Bangladesh or Bulgaria or "Bosnia and Herzegovina" or Belarus or Belize or Bolivia or Brazil or brasil or Bhutan or Botswana or "Central African Republic" or China or "Cote d'Ivoire" or "ivory coast" or Cameroon or Congo or "Democratic Republic of Congo" or Colombia or Comoros or "Cabo Verde" or "Costa Rica" or Cuba or Djibouti or Dominica or "Dominican Republic" or Algeria or Ecuador or Egypt or Eritrea or Ethiopia or Fiji or Micronesia or Gabon or Georgia or Ghana or Guinea or Gambia or "Guinea-Bissau" or "Equatorial Guinea" or Grenada or Guatemala or Guyana or Honduras or Haiti or Indonesia or India or Iran or Iraq or Jamaica or Jordan or Kazakhstan or Kenya or "Kyrgyz Republic" or Cambodia or Kiribati or Lao or laos or Lebanon or Liberia or Libya or "St. Lucia" or "saint lucia" or "Sri Lanka" or Lesotho or Morocco or Moldova or Madagascar or Maldives or Mexico or "Marshall Islands" or "North Macedonia" or Mali or Myanmar or Montenegro or Mongolia or Mozambique or Mauritania or Mauritius or Malawi or Malaysia or Namibia or Niger or Nigeria or Nicaragua or Nepal or Pakistan or Panama or Peru or Philippines or "Papua New Guinea" or "Democratic People's Republic of Korea" or Paraguay or "West Bank and Gaza" or Romania or "Russian Federation" or russia or Rwanda or Sudan or Senegal or "Solomon Islands" or "Sierra Leone" or "El Salvador" or Somalia or Serbia or "South Sudan" or "Sao Tome and Principe" or Suriname or Eswatini or "Syrian Arab Republic" or Chad or Togo or Thailand or Tajikistan or Turkmenistan or "Timor Leste" or Tonga or Tunisia or Turkey or Tuvalu or Tanzania or Uganda or Ukraine or Uzbekistan or "St. Vincent and the Grenadines" or "saint vincent and the grenadines" or Vietnam or Vanuatu or Samoa or Kosovo or Yemen or " South Africa" or Zambia or Zimbabwe).mp.
10.	5 and 8 and 9
11.	(random$ or placebo$ or single blind$ or double blind$ or triple blind$).ti,ab.
12.	exp randomized controlled trials/
13.	(random sampl$ or random digit$ or random effect$ or random survey or random regression).ti,ab. not exp randomized controlled trial/
14.	(11 or 12) not 13
15.	exp cohort studies/
16.	exp longitudinal studies/
17.	cohort$.tw.
18.	(((nonequivalent or non equivalent) adj3 control$) or posttest$ or post test$ or pre test$ or pretest$ or quasi experiment$ or quasiexperiment$ or timeseries or time series).tw.
19.	(time series or nonequivalent group* or regression discontinuity or propensity score or synthetic control method or difference-in-difference or natural experiment*).ti,ab,kw.
20.	(time series or quasi-experimental design).af.
21.	14 or 15 or 16 or 17 or 18 or 19 or 20
22.	10 and 21
23.	limit 22 to yr="2000 -Current"
24.	(comment or editorial or "conference abstract" or "meeting abstract" or "meta-analysis" or review or "systematic review").pt.
25.	23 not 24
26.	limit 25 to english
27.	limit 25 to french
28.	limit 25 to spanish
29.	limit 25 to portuguese
30.	26 or 27 or 28 or 29

Appendix 4.2.4 Search strategy for Web of Science.

1.	AB=(((food or nutrition* OR diet* OR eating or sugar* OR beverage* OR drink* OR fat OR fats OR fatty OR fruit* OR vegetable* OR meal* OR menu*) AND (program* OR intervention* OR policy or policies OR action* OR plan* OR law OR laws OR legislation OR strategy OR tax* OR subsid* OR voucher* OR coupon* OR educat* OR campaign* OR fortif* OR scheme* OR reformulat* OR guideline* OR standard* OR market* OR label* OR packag* OR advert* OR transfer* OR basket or supplement*)))
2.	AB=((Breastfeed* OR "breast feed*" OR "complementary feed*" OR "child* feeding" OR "infant feed*") AND (protect* OR support* OR promot* OR improv* OR empower* OR service* OR provi*))
3.	AB=((wom?n* OR female* OR "maternal health" OR "maternal schooling" OR "maternal education" OR "family plan*" OR "drink* water" OR sanitation or hygiene* OR child) and (protect* OR support* OR promot* OR improv* OR empower* OR service* OR provi* OR program*))
4.	AB=(("conditional cash transfer*" OR "social welfare program*" OR "social safety net*" OR "trad* agreement" OR "foreign direct invest*" OR "trad* tarif*" OR "capacity investment*" OR "domestic resource mobili?ation" OR ((agriculture*) AND (program* OR intervention* OR policy OR policies OR action* OR plan* OR law OR laws OR legislation or strategy)) OR "leadership program*"))

5.	#4 OR #3 OR #2 OR #1
6.	AB=((undernutrition OR underweight OR "low weight" OR stunt* OR "height for age" OR "length for age" OR "stature for age" OR (short AND (height OR stature)) OR (growth AND (disorder OR impair* OR retard OR delay*)) OR wast* OR "low weight for height" OR thin* OR "low BMI" OR "low body mass index" OR "iron deficienc*" OR ane?mi* OR "low birth weight" OR LBW OR "small for gestational age"))
7.	AB=((overnutrition OR overweight OR "over weight" OR obes* OR ((excessive OR high or abnormal) AND (bodyweight OR weight)) OR "high weight for height" OR "high bmi for age" OR "high waist to hip ratio" OR "high waist circumference" OR "high waist to stature ratio" OR "high waist to height ratio" OR macrosomia OR "high birth weight" OR "large for gestational age"))
8.	#7 AND #6
9.	AB=((Afghanistan OR Angola OR Albania OR Argentina OR argentine OR Armenia OR "American Samoa" OR Azerbaijan or Burundi OR Benin OR "Burkina Faso" OR Bangladesh OR Bulgaria OR "Bosnia and Herzegovina" OR Belarus OR Belize OR Bolivia OR Brazil OR brasil OR Bhutan OR Botswana OR "Central African Republic" OR China OR "Cote d'Ivoire" OR "ivory coast" OR Cameroon or Congo OR "Democratic Republic of Congo" OR Colombia OR Comoros OR "Cabo Verde" OR "Costa Rica" OR Cuba OR Djibouti OR Dominica OR "Dominican Republic" OR Algeria OR Ecuador OR Egypt OR Eritrea OR Ethiopia OR Fiji OR Micronesia OR Gabon OR Georgia OR Ghana OR Guinea OR Gambia OR "Guinea-Bissau" OR "Equatorial Guinea" OR Grenada OR Guatemala OR Guyana OR Honduras OR Haiti OR Indonesia OR India OR Iran OR Iraq OR Jamaica OR Jordan OR Kazakhstan OR Kenya OR "Kyrgyz Republic" OR Cambodia OR Kiribati OR Lao OR laos OR Lebanon OR Liberia OR Libya OR "St. Lucia" OR "saint lucia" OR "Sri Lanka" OR Lesotho OR Morocco OR Moldova OR Madagascar OR Maldives OR Mexico OR "Marshall Islands" OR "North Macedonia" OR Mali OR Myanmar OR Montenegro OR Mongolia OR Mozambique OR Mauritania OR Mauritius OR Malawi OR Malaysia OR Namibia OR Niger OR Nigeria OR Nicaragua OR Nepal OR Pakistan OR Panama OR Peru OR Philippines OR "Papua New Guinea" OR "Democratic People's Republic of Korea" OR Paraguay OR "West Bank and Gaza" OR Romania OR "Russian Federation" OR russia OR Rwanda OR Sudan OR Senegal OR "Solomon Islands" OR "Sierra Leone" OR "El Salvador" OR Somalia or Serbia OR "South Sudan" OR "Sao Tome and Principe" OR Suriname OR Eswatini OR "Syrian Arab Republic" OR Chad OR Togo OR Thailand OR Tajikistan OR Turkmenistan OR "Timor Leste" OR Tonga OR Tunisia OR Turkey OR Tuvalu OR Tanzania OR Uganda OR Ukraine OR Uzbekistan OR "St. Vincent and the Grenadines" OR "saint vincent and the grenadines" OR Vietnam OR Vanuatu OR Samoa OR Kosovo OR Yemen OR " South Africa" OR Zambia OR Zimbabwe))
10.	ALL=(randomised OR randomized OR randomisation OR randomization OR placebo* OR (random* AND (allocat* OR assign*)) OR (blind* AND (single OR double OR treble OR triple)))
11.	ALL=(((cohort* OR longitudinal* OR prospective OR observational) AND (stud* OR analysis)) OR follow up)
12.	ALL=(((nonequivalent OR non equivalent) AND (control*)) OR posttest* OR post test* OR pre test* OR pretest* OR quasi experiment* OR quasiexperiment* OR timeseries OR time series)
13.	#12 OR #11 OR #10
14.	#13 AND #9 AND #8 AND #5

Note: Language filters (English, Spanish, Portuguese and French) were applied manually after running the search query.

Appendix 4.3 Data extraction

Appendix 4.3.1 Data extraction template for descriptive information of studies.

**Data item**	**Extracted information**
**General information**	
Title	
Year of publication	
First author (Last name)	
Journal	
Country in which study was conducted	
Aim of study	
Study design	
Other methodological info that seems relevant	
**Intervention**	
Type of intervention	- Nutrition-direct intervention<br>- Nutrition-sensitive intervention<br>- Combined
Institution that initiated intervention(s)	
Describe intervention	
Timing of intervention and data collection	
**Population**	
Data source	
Population description	
Comparison	
Comparator group?	- Yes<br>- No
Description of comparator group (only if yes above)	
**Key Messages**	
Summarize main findings	
Conclusion (2 - 3 sentences)	
**Limitations**	
Main limitation mentioned in study	
**Disclosure statements**	
Study funding sources	
Possible conflicts of interest for study authors	
Anything else considered relevant and not included above	

Appendix 4.3.2 Data extraction template for categorical variables.

	N	Unadjusted effect size (Estimate, CI, p-value)	Adjusted effect size (Estimate, Ci, P-value)	Variables controlled for	Type of effect measure
Underweight					
Wasting					
Thinness					
Stunted					
Low birth weight					
Small for gestational age					
Anaemic					
Overweight					
Obesity					
Macrosomia					
Other outcome 1					
Other outcome 2					
Other outcome 3					
Definition categorical measures if not according to WHO cut-off:					
Definition other outcomes if applicable					

Appendix 4.3.3 Data extraction template for continuous variables.

	N	Unadjusted EE (estimate, CI, p-value)	Adjusted EE (estimate, CI, p-value)	Variables controlled for	Type of effect estimate
weight-for-age z-score					
weight-for-height z-score					
bmi-for-age z-score					
height-for-age z-score					
Waist circumference (unit)					
Haemoglobin level (unit)					

BMI					
Weight					
Height					
Other outcome 1					
Other outcome 2					
Other outcome 3					
Definition categorical measures if not according to WHO cut-off:					
Definition other outcomes if applicable					

Appendix 4.3.4 Data extraction template for stratified results for categorical variables.

	N str.1	Effect size str.1	N str.2	Effect size str.2	N str.3	Effect size str.3	N str.4	Effect size str.4	N str.5	Effect size str.5	Type of effect measure
Underweight											
Wasting											
Thinness											
Stunted											
Low birth weight											
Anaemia											
Overweight											
Obesity											
Macrosomia											
Other 1											
Other 2											
Other 3											

Appendix 4.3.5 Data extraction template for stratified results for continuous variables.

	N str.1	Effect size str.1	N str.2	Effect size str.2	N str.3	Effect size str.3	N str.4	Effect size str.4	N str.5	Effect size str.5	Type of effect measure
Weight-for-age z score											
BMI-for-age z score											
Height-for-age z score											
Waist circumference											
Haemoglobin level											
BMI											
Weight											
Height											
Other 1											
Other 2											
Other 3											

Appendix 4.4 Quality assessment template [276]

**Selection Bias**	
1. Are the individuals selected to participate in the study likely to be representative of the target population?	- Very likely<br>- Somewhat likely<br>- Not likely
2. What percentage of selected individuals agreed to participate?	- 80 - 100% agreement<br>- 60 - 79% agreement<br>- less than 60% agreement<br>- Not applicable<br>- Can't tell
3. Overall rating for selection bias	- Strong<br>- Moderate<br>- Weak
**Study design**	
1. Indicate the study design	- Randomized Controlled Trial<br>- Controlled clinical trial<br>- Cohort analytic (two group pre + post)<br>- Case-control<br>- Cohort (one group pre + post (before and after))<br>- Interrupted time series<br>- Other specify<br>- Can't tell
2. Was the study described as randomised? If NO, go to confounders directly.	- Yes<br>- No
3. If yes, was the method of randomisation described? (See dictionary)	- Yes<br>- No
4. If yes, was the method appropriate? (See dictionary)	- Yes<br>- No
5. Overall rating for study design	- Strong<br>- Moderate<br>- Weak
**Confounders**	
1. Were there important differences between groups prior to the intervention?	- Yes<br>- No<br>- Can't tell
2. If yes, indicate the percentage of relevant confounders that were controlled (either in the design (e.g., stratification, matching) or analysis)? (Relevant confounders are Race, Sex, Marital status / family, Age, SES, education, health status, pre-intervention score on outcome measure)	- 80 – 100% (most)<br>- 60 – 79% (some)<br>- Less than 60% (few or none)<br>- Can’t Tell
3. Overall handling of confounding	- Strong<br>- Moderate<br>- Weak
**Data collection methods**	
1. Were data collection tools shown to be valid?	- Yes<br>- No<br>- Can't tell
2. Were data collection tools shown to be reliable?	- Yes<br>- No<br>- Can't tell
3. Overall rating data collection methods	- Strong<br>- Moderate<br>- Weak
**Withdrawals and drop-outs**	
1. Were withdrawals and drop-outs reported in terms of numbers and/or reasons per group?	- Yes<br>- No<br>- Can't tell<br>- N/A (i.e., one-time surveys or interviews)
2. Indicate the percentage of participants completing the study. (If the percentage differs by groups, record the lowest).	- 80 -100%<br>- 60-79%<br>- less than 60%<br>- Can't tell<br>- N/A (i.e., Retrospective case-control)
3. Overall rating Withdrawals and drop-outs	- Strong<br>- Moderate<br>- Weak
**Intervention integrity**	
1. What percentage of participants received the allocated intervention of interest?	- 80 -100%<br>- 60-79%

	- less than 60%<br>- Can't tell
2. Was the consistency of the intervention measured?	- Yes<br>- No<br>- Can’t tell
3. Is it likely that subjects received an unintended intervention (contamination or co-intervention) that may have influenced the results)	- Yes<br>- No<br>- Can’t tell
**Analyses**	
1. Indicate the unit of allocation	- Community<br>- Organisation / institution<br>- Practice / office<br>- Individual
2. Indicate the unit of analysis	- Community<br>- Organisation / institution<br>- Practice / office<br>- Individual
3. Are the statistical methods appropriate for the study design?	- Yes<br>- No<br>- Can’t tell
4. Is the analysis performed by intervention allocation status (i.e., intention to treat) rather than actual intervention received?	- Yes<br>- No<br>- Can’t tell
**Overall quality rating paper**	- High (no weak ratings)<br>- Moderate (one weak rating)<br>- Low (two or more weak ratings)

References

1. Burlandy L, Rocha C, Maluf R. Integrating Nutrition into Agricultural and Rural Development Policies: The Brazilian Experience of Building an Innovative Food and Nutrition Security Approach. International Symposium on Food and Nutrition Security - Food-Based Approaches for Improving Diets and Raising Levels of Nutrition; 2010 Dec 07-09; United Nat Headquarters, Rome, ITALY: Cabi Publishing-C a B Int; 2010. p. 101-12.
2. Sanchez B, Mendoza ME, Avila Rosas H. [Assessment of a preconception preventive program in a community]. *Ginecol Obstet Mex* 2000; **68**: 20-6.
3. Okezie CA, Nwosu AZ. The effect of agricultural commercialization on the nutritional status of cocoa growing households in Ikwuano LGA of Abia State Nigeria. *International Journal of Agriculture and Rural Development* 2007; **9**: 12-5.
4. Fenn B, Sangrasi GM, Puett C, Trenouth L, Pietzsch S. The REFANI Pakistan study-a cluster randomised controlled trial of the effectiveness and cost-effectiveness of cash-based transfer programmes on child nutrition status: study protocol. *BMC Public Health* 2015; **15**: 10.
5. Acero CG, Martinez S, Perez-Exposito A, Winters S. Effect of an innovative behavioural change strategy and small-quantity lipid-based nutrient supplements on stunting and obesity in children in Baja Verapaz, Guatemala: protocol for a randomised control trial. *Bmj Open* 2020; **10**(7): 9.
6. Kittisakmontri K, Lanigan J, Wells JCK, Fewtrell M. The Impact of Dietary Protein in Complementary Foods on Infant Growth and Body Composition in a Population Facing the Double Burden of Malnutrition: Protocol for a Multicenter, Prospective Cohort Study. *JMIR Res Protoc* 2020; **9**(9): e18112.
7. Mahmudiono T, Nindya TS, Andrias DR, Megatsari H, Rosenkranz RR. The effectiveness of nutrition education for overweight/obese mothers with stunted children (NEO-MOM) in reducing the double burden of malnutrition in Indonesia: study protocol for a randomized controlled trial. *BMC Public Health* 2016; **16**: 486.
8. Pesu H, Mutumba R, Mbabazi J, et al. The Role of Milk Protein and Whey Permeate in Lipid-based Nutrient Supplements on the Growth and Development of Stunted Children in Uganda: A Randomized Trial Protocol (MAGNUS). *Curr* 2021; **5(5) (no pagination)**.
9. Sangalang SO, Medina SAJ, Ottong ZJ, et al. Study protocol protocol for a trial assessing the impacts of school-based wash interventions on children's health literacy, handwashing, and nutrition status in low-and middle-income countries. *Int J Environ Res Public Health* 2021; **18(1)**: 1-19.
10. Malpeli A, Ferrari MG, Varea A, et al. Short-term evaluation of the impact of a fortified food aid program on the micronutrient nutritional status of Argentinian pregnant women. *Biol Trace Elem Res* 2013; **155**(2): 176-83.
11. Sekiyama M, Roosita K, Ohtsuka R. Locally sustainable school lunch intervention improves hemoglobin and hematocrit levels and body mass index among elementary schoolchildren in rural West Java, Indonesia. *Nutrients* 2017; **9(8) (no pagination)**.
12. Varea A, Malpeli A, Etchegoyen G, et al. Short-term evaluation of the impact of a food program on the micronutrient nutritional status of Argentinean children under the age of six. *Biol Trace Elem Res* 2011; **143**(3): 1337-48.
13. Al-Zeidaneen SA, Al-Bayyari NS, Al-Zidaneen MA. The impact of maternal employment on infant weight-, length- and BMI-for-age based upon WHO growth chart standards. *International Journal of Child Health and Nutrition* 2017; **6**(3): 116-22.
14. Cheng G, Sha TT, Gao X, et al. Effects of Maternal Prenatal Multi-Micronutrient Supplementation on Growth and Development until 3 Years of Age. *Int J Environ Res Public Health* 2019; **16**(15): 16.

15. DeLacey E, Hilberg E, Allen E, et al. Nutritional status of children living within institution-based care: a retrospective analysis with funnel plots and control charts for programme monitoring. *BMJ Open* 2021; **11**(12).
16. Dong Y, Lau PWC, Dong B, et al. Trends in physical fitness, growth, and nutritional status of Chinese children and adolescents: a retrospective analysis of 1.5 million students from six successive national surveys between 1985 and 2014. *Lancet Child Adolesc Health* 2019; **3**(12): 871-80.
17. Faber M, Jaarsveld PJv, Kunneke E, Kruger HS, Schoeman SE, Stuijvenberg MEv. Vitamin A and anthropometric status of South African preschool children from four areas with known distinct eating patterns. *Nutrition* 2015; **31**(1): 64-71.
18. Gurung TR, Kodkany BS, Shivanand M, Neginhal VS. Association between standard of living and underweight & overweight among likely to conceive women in rural North Karnataka-baseline result of DBW cohort. *Indian Journal of Community Health* 2018; **30**(2): 145-50.
19. Haschke F, Binder C, Huber-Dangl M, Haiden N. Early-Life Nutrition, Growth Trajectories, and Long-Term Outcome. 90th Nestle-Nutrition-Institute Workshop; 2017 Oct 30-Nov 01; Lausanne, SWITZERLAND: Karger; 2017. p. 107-20.
20. Ibrahim KHH, S. L.; Schonfeldt, H. The effect of smallholder land tenure on child malnutrition in Nigeria. *Land Use Pol* 2022; **119**.
21. Khonje MG, Ecker O, Qaim M. Effects of modern food retailers on adult and child diets and nutrition. *Nutrients* 2020; **12(6)**: 1-17.
22. Lee SJ, Hashmi AH, Min AM, et al. Short maternal stature and gestational weight gain among refugee and migrant women birthing appropriate for gestational age term newborns: a retrospective cohort on the Myanmar-Thailand border, 2004-2016. *BMJ Global Health* 2021; **6**(2): 02.
23. Locks LM, Manji KP, Kupka R, et al. High Burden of Morbidity and Mortality but Not Growth Failure in Infants Exposed to but Uninfected with Human Immunodeficiency Virus in Tanzania. *J Pediatr* 2017; **180**: 191-9.e2.
24. Po JYT, Bukania Z, Muhammad L, Hickey GM. Associations between Maternal Participation in Agricultural Decision-Making and Child Nutrition in Semiarid Kenya. *Journal of Hunger and Environmental Nutrition* 2020; **15(5)**: 712-37.
25. Potdar RD, Sahariah SA, Gandhi M, et al. Improving women's diet quality preconceptionally and during gestation: Effects on birth weight and prevalence of low birth weight - A randomized controlled efficacy trial in India (Mumbai maternal nutrition project). *American Journal of Clinical Nutrition* 2014; **100(5)**: 1257-68.
26. Santos AP, Mazzeti CMD, Franco MDP, et al. Nutritional status and environmental and health conditions of Pataxo indigenous children, Minas Gerais State, Brazil. *Cad Saude Publica* 2018; **34**(6): 8.
27. Silva ND, Ribeiro-Silva RD, Rasella D, et al. Shifts towards overweight and double burden of malnutrition among socio-economically vulnerable children: a longitudinal ecological analysis of Brazilian municipalities. *Public Health Nutr* 2021; **24**(15): 4908-17.
28. Tian Y, Jiang C, Cai R, et al. BMI, leisure-time physical activity, and physical fitness in adults in China: Results from a series of national surveys, 2000-14. *The Lancet Diabetes and Endocrinology* 2016; **4(6)**: 487-97.
29. Zhang XH, Xu J, Chen DQ, Guo LF, Qiu LQ. Effectiveness of treatment to improve pregnancy outcomes among women with syphilis in Zhejiang Province, China. *Sex Transm Infect* 2016; **92**(7): 537-41.
30. Zheng X, Wu Q, Weng D, Fu Y, Yue D, Wang Y. Adherence to supplemental vitamin D intake and infant weight gain: a retrospective cohort study in rural southwestern China. *J Int Med Res* 2020; **48**(11): 300060520969311.

31. Abreu AM, Young RR, Buchanan A, et al. Maternal Blood Pressure in Relation to Prenatal Lipid-Based Nutrient Supplementation and Adverse Birth Outcomes in a Ghanaian Cohort: A Randomized Controlled Trial and Cohort Analysis. *J Nutr* 2021; **151**(6): 1637-45.
32. Abril E, Rascon Loreto C, Arenas Monreal L, Bonilla Fernandez P, Hernandez Perez H, Cuevas Bahena S. Promotion of healthy eating habits in a primary school in Hermosillo, Son., Mexico. [Spanish]. *Revista Salud Publica y Nutricion* 2009; **10**(1).
33. Adom T, Steiner-Asiedu M, Sakyi-Dawson E, Anderson AK. Effect of fortification of maize with cowpea and iron on growth and anaemia status of children. *African Journal of Food Science* 2010; **4**(4): 136-42.
34. Agdeppa IA, Zamora JAT. The effects of coconut skim milk and coco-dairy milk blend on the nutritional status of schoolchildren. *Journal of Nutrition and Metabolism* 2022; **6793866**(36).
35. Agrasada GV, Gustafsson J, Kylberg E, Ewald U. Postnatal peer counselling on exclusive breastfeeding of low-birthweight infants: a randomized, controlled trial. *Acta Paediatrica* 2005; **94**(8): 1109-15.
36. Aguila DV, Dorado JB, Capanzana MV. Government-industry-academia alliance: a multisectoral collaboration for improved nutrition of children and well-being of mothers. *Malays J Nutr* 2023; **29**(1): 63-75.
37. Ahmad A, Madanijah S, Dwiriani CM, Kolopaking R. Effect of nutrition education and multi-nutrient biscuit interventions on nutritional and iron status: A cluster randomized control trial on undernourished children aged 6-23 months in Aceh, Indonesia. *J Nutr Sci Vitaminol (Tokyo)* 2020; **66(Supplement)**: S380-S90.
38. Akeredolu IA, Osisanya JO, Okafor JC, Seriki-Mosadolorun J. Pregnancy outcomes of women in Lagos State: Is nutrition education responsible? *Pakistan Journal of Nutrition* 2014; **13(1)**: 7-11.
39. Aldana-Parra F, Olaya G, Fewtrell M. Effectiveness of a new approach for exclusive breastfeeding counselling on breastfeeding prevalence, infant growth velocity and postpartum weight loss in overweight or obese women: protocol for a randomized controlled trial. *Int Breastfeed J* 2020; **15**: 2.
40. Andrade S, Lachat C, Cardon G, et al. Two years of school-based intervention program could improve the physical fitness among Ecuadorian adolescents at health risk: subgroups analysis from a cluster-randomized trial. *BMC Pediatr* 2016; **16**(51).
41. Angeles-Agdeppa I, Magsadia CR, Capanzana MV. Fortified juice drink improved iron and zinc status of schoolchildren. *Asia Pacific Journal of Clinical Nutrition* 2011; **20**(4): 535-43.
42. Arsenault JE, Mora-Plazas M, Forero Y, et al. Provision of a school snack is associated with vitamin B-12 status, linear growth, and morbidity in children from Bogota, Colombia. *J Nutr* 2009; **139**(9): 1744-50.
43. Argaw A, de Kok B, Toe LC, et al. Fortified balanced energy-protein supplementation during pregnancy and lactation and infant growth in rural Burkina Faso: A 2 x 2 factorial individually randomized controlled trial. *PLoS Med* 2023; **20(2) (no pagination)**.
44. Arunambika C, Akash S, Ramesh A, Anu T, Ashok D, Jeeva S. Fortification of breast milk with preterm formula powder vs human milk fortifier in preterm neonates: a randomized noninferiority trial. *Jama, Pediatr* 2021; **175**(8): 790-6.
45. Ashorn P, Alho L, Ashorn U, et al. The impact of lipid-based nutrient supplement provision to pregnant women on newborn size in rural Malawi: a randomized controlled trial. *Am J Clin Nutr* 2015; **101**(2): 387-97.
46. Assis AM, Costa PR, da Silva Mda C, et al. Effectiveness of the Brazilian Conditional Cash Transfer Program--Bolsa Alimentacao--on the variation of linear and ponderal increment in children from northeast of Brazil. *Nutr Hosp* 2015; **31**(6): 2786-94.

47. Augusto RA, Souza JM. Effectiveness of a supplementary feeding program in child weight gain. *Rev Saude Publica* 2010; **44**(5): 793-801.
48. Azimi F, Esmaillzadeh A, Alipoor E, Moslemi M, Yaseri M, Hosseinzadeh-Attar MJ. Effect of a newly developed ready-to-use supplementary food on growth indicators in children with mild to moderate malnutrition. *Public Health* 2020; **185**: 290-7.
49. Barber SL, Gertler PJ. The impact of Mexico's conditional cash transfer programme, Oportunidades, on birthweight. *Tropical Medicine and International Health* 2008; **13**(11): 1405-14.
50. Barber SL, Gertler PJ. Empowering women: how Mexico's conditional cash transfer programme raised prenatal care quality and birth weight. *J* 2010; **2**(1): 51-73.
51. Barennes H, Houdart L, de Courville C, Barennes F. Spirulina as a daily nutritional supplement of young pre-school Cambodian children of deprived settings: a single-blinded, placebo-controlled, cross-over trial. *BMC Pediatr* 2022; **22**(1): 8.
52. Barth-Jaeggi T, Moretti D, Kvalsvig J, et al. In-home fortification with 2.5 mg iron as NaFeEDTA does not reduce anaemia but increases weight gain: a randomised controlled trial in Kenyan infants. (Special Issue: Policy, program and innovation in complementary feeding.). *Maternal and Child Nutrition* 2015; **11**(s4): 151-62.
53. Beckmann J, Nqweniso S, Ludyga S, et al. Evaluation of a Physical Activity and Multi-Micronutrient Intervention on Cognitive and Academic Performance in South African Primary Schoolchildren. *Nutrients* 2022; **14(13) (no pagination)**.
54. Behrman JR, Calderon MC, Preston SH, Hoddinott J, Martorell R, Stein AD. Nutritional supplementation in girls influences the growth of their children: Prospective study in Guatemala. *Am J Clin Nutr* 2009; **90(5)**: 1372-9.
55. Bisimwa G, Owino VO, Bahwere P, et al. Randomized controlled trial of the effectiveness of a soybean-maize-sorghum-based ready-to-use complementary food paste on infant growth in South Kivu, Democratic Republic of Congo. *Am J Clin Nutr* 2012; **95**(5): 1157-64.
56. Bliss J, Jensen N, Thiede B, et al. Factors Associated With the Risk of Acute Malnutrition Among Children Aged 6 to 36 Months in Households Targeted by an Emergency Cash Transfer Program. *Food Nutr Bull* 2016; **37**(3): 387-400.
57. Bliznashka L, Sudfeld CR, Garba S, et al. Prenatal supplementation with multiple micronutrient supplements or medium-quantity lipid-based nutrient supplements has limited effects on child growth up to 24 months in rural Niger: a secondary analysis of a cluster randomized trial. *Am J Clin Nutr* 2022; **115**(3): 738-48.
58. Briaux J, Martin-Prevel Y, Carles S, et al. Evaluation of an unconditional cash transfer program targeting children's first-1,000-days linear growth in rural Togo: A cluster-randomized controlled trial. *PLoS Med* 2020; **17**(11): 29.
59. Brits H, Joubert G, Eyman K, et al. An assessment of the integrated nutrition programme for malnourished children aged six months to five years at primary healthcare facilities in mangaung, free state, South Africa. *South African Family Practice* 2017; **59(6)**: 214-8.
60. Brown KH, Romana DLd, Arsenault JE, Peerson JM, Penny ME. Comparison of the effects of zinc delivered in a fortified food or a liquid supplement on the growth, morbidity, and plasma zinc concentrations of young Peruvian children. (Special Section: Maternal nutrition and optimal infant feeding practices.). *American Journal of Clinical Nutrition* 2007; **85**(2): 538-47.
61. Brown N, Sasidharan CK, Fisher D. Early growth and markers of cardiovascular risk in Keralan children in the Integrated Child Development Scheme. *Public Health Nutrition* 2010; **13**(7): 1042-8.

62. Callaghan-Gillespie M, Schaffner AA, Garcia P, et al. Trial of ready-to-use supplemental food and corn-soy blend in pregnant Malawian women with moderate malnutrition: a randomized controlled clinical trial. *Am J Clin Nutr* 2017; **106**(4): 1062-9.
63. Cameron L, Olivia S, Manisha S. Scaling up sanitation: evidence from an RCT in Indonesia. *Journal of Development Economics* 2019; **138**: 1-16.
64. Campbell RK, Hurley KM, Shamim AA, et al. Effect of complementary food supplementation on breastfeeding and home diet in rural Bangladeshi children. *American Journal of Clinical Nutrition* 2016; **104**(5): 1450-8.
65. Cao J, Wei X, Tang X, et al. Effects of egg and vitamin A supplementation on hemoglobin, retinol status and physical growth levels of primary and middle school students in Chongqing, China. *Asia Pacific Journal of Clinical Nutrition* 2013; **22**(2): 214-21.
66. Carrasco Quintero MdR, Ortiz Hernandez L, Roldan Amaro JA, Chavez Villasana A, Aguirre Arenas J, Raul Aguilar Carrasco F. Effect of consumption of corn flour enriched with soja on nutrition status of indigenous women of Mexico. [Spanish]. *Rev Esp Salud Publica* 2013; **87**(3): 293-302.
67. Carvalhaes MAdL, D'Aquino Benicio MH, Barros AJD. Social support and infant malnutrition: A case-control study in an urban area of Southeastern Brazil. *British Journal of Nutrition* 2005; **94(3)**: 383-9.
68. Chanani S, Waingankar A, Shah More N, Pantvaidya S, Fernandez A, Jayaraman A. Effectiveness of NGO-government partnership to prevent and treat child wasting in urban India. *Maternal and Child Nutrition* 2019; **15(Supplement 1) (no pagination)**.
69. Chen J, Jin M. The effectiveness of an egg-based intervention on improving the nutrition of poor school-age children in China: a quasi-experimental assessment. *Nutrition* 2023; **109**(44).
70. Christian P, Shaikh S, Shamim AA, et al. Effect of fortified complementary food supplementation on child growth in rural Bangladesh: A cluster-randomized trial. *Int J Epidemiol* 2015; **44(6)**: 1862-76.
71. Chowdhury M, Raynes-Greenow C, Kelly P, et al. The Impact of Antenatal Balanced Plate Nutrition Education for Pregnant Women on Birth Weight: A Cluster Randomised Controlled Trial in Rural Bangladesh. *Nutrients* 2022; **14**(21).
72. Colunga DP, Martinez JG, Hernandez JR. Effect of the "ESNUT" Nutritional Stabilization Program on food consumption, BMI, body composition and physical activity in Mexican university students. *Nutricion Clinica Y Dietetica Hospitalaria* 2021; **41**(1): 108-15.
73. Da Silva Rocha D, Diniz Capanema F, Pereira Netto M, et al. Effectiveness of fortification of drinking water with iron and vitamin C in the reduction of anemia and improvement of nutritional status in children attending day-care centers in Belo Horizonte, Brazil. *Food Nutr Bull* 2011; **32(4)**: 340-6.
74. Dawood HAW, Aziz AR. Efficacy of the health promotion model-based intervention in enhancing the health responsibility of middle school female student: A randomized controlled trial. *Indian Journal of Public Health Research and Development* 2019; **10(9)**: 670-5.
75. DeBoer MD, Elwood SE, Platts-Mills JA, et al. Sex Differences in Early Childhood Growth in a Resource-Limited Setting: A Secondary Analysis of the Early Life Interventions in Childhood Growth and Development in Tanzania (ELICIT) Study. *J Nutr* 2022; **152**(2): 579-86.
76. Deepthi K, Rajiv S, Prabhu S, et al. The first 1000 days of life: prenatal and postnatal risk factors for morbidity and growth in a birth cohort in southern India. *BMJ Open* 2014; **4**(7).

77. Delisle HF, Receveur O, Agueh V, Nishida C. Pilot project of the Nutrition-Friendly School Initiative (NFSI) in Ouagadougou, Burkina Faso and Cotonou, Benin, in West Africa. *Glob Health Promot* 2013; **20**(1): 39-49.
78. Dennis EG, Sie A, Ouermi L, et al. Short-term weight gain among preschool children in rural Burkina Faso: a secondary analysis of a randomised controlled trial. *BMJ Open* 2019; **9**(7): e029634.
79. Dereje T, Dessalegn T, Tefera B. Effect of a theory-based nutrition education intervention during pregnancy through male partner involvement on newborns' birth weights in southwest Ethiopia. A three-arm community based quasi-experimental study. *PLoS ONE* 2023; **18**(1).
80. Desalegn M, Terefe B, Bikila H. Effect of food insecurity and other possible factors associated with low birth weight among mothers who gave birth to live newborns in West Ethiopia: a facility-based unmatched case-control study. *Nutrition and Dietary Supplements* 2021; **13**(133-143): 133-43.
81. Destaw Z, Wencheko E, Kidane S, et al. Impact of school meals on educational outcomes in Addis Ababa, Ethiopia. *Public Health Nutr* 2022; **25**(9): 2614-24.
82. Dhaded SM, Hambidge KM, Ali SA, et al. Preconception nutrition intervention improved birth length and reduced stunting and wasting in newborns in South Asia: The Women First Randomized Controlled Trial. *PLoS One* 2020; **15(1) (no pagination)**.
83. Dijkhuizen MA, Wieringa FT, West CE, Martuti S, Muhilal. Effects of iron and zinc supplementation in Indonesian infants on micronutrient status and growth. *J Nutr* 2001; **131(11)**: 2860-5.
84. Dijkhuizen MA, Winichagoon P, Wieringa FT, et al. Zinc supplementation improved length growth only in anemic infants in a multi-country trial of iron and zinc supplementation in South-East Asia. *Journal of Nutrition* 2009; **139**(10): 1969-75.
85. Ding YX, Ma L, Jia P, et al. Longitudinal effects of school policies on children's eating behaviors and weight status: findings from the childhood obesity study in China megacities. *Int J Obes* 2023; **47**(1): 17-23.
86. Ebrahimi S, Pormahmodi A, Kamkar A. Study of zinc supplementation on growth of schoolchildren in Yasuj, Southwest of Iran. *Pakistan Journal of Nutrition* 2006; **5**(4): 341-2.
87. Echague G, Funes P, Diaz V, et al. Evaluation of anemia after nutritional interventions in children living in rural communities in Caazapa, Paraguay. [Spanish]. *Pediatria* 2019; **46**(2): 103-9.
88. Ekoe T, Bianpambe OI, Nguefack F, et al. Efficacy of an iron-fortified infant cereal to reduce the risk of iron deficiency anemia in young children in East Cameroon. *Food Sci Nutr* 2020; **8**(7): 3566-77.
89. Engebretsen IM, Jackson D, Fadnes LT, et al. Growth effects of exclusive breastfeeding promotion by peer counsellors in sub-Saharan Africa: the cluster-randomised PROMISE EBF trial. *BMC Public Health* 2014; **14**(633).
90. Fabiansen C, Phelan KPQ, Cichon B, et al. Short Malnourished Children and Fat Accumulation With Food Supplementation. *Pediatrics* 2018; **142**(3): 09.
91. Fatima S, Malkova D, Wright C, Gerasimidis K. Impact of therapeutic food compared to oral nutritional supplements on nutritional outcomes in mildly underweight healthy children in a low-medium income society. *Clin Nutr* 2018; **37(3)**: 858-63.
92. Fenn B, Myatt M, Mates E, Black RE, Wilkinson C, Khara T. Effects on child growth of a reduction in the general food distribution ration and provision of small-quantity lipid-based nutrient supplements in refugee camps in eastern Chad. *BMJ nutr* 2021; **4**(1): 235-42.
93. Fernald LCH, Gertler PJ, Neufeld LM. 10-year effect of Oportunidades, Mexico's conditional cash transfer programme, on child growth, cognition, language, and behaviour: a longitudinal follow-up study. *Lancet* 2009; **374**(9706): 1997-2005.

94. Fianu A, Bourse L, Naty N, et al. Long-Term Effectiveness of a Lifestyle Intervention for the Primary Prevention of Type 2 Diabetes in a Low Socio-Economic Community--An Intervention Follow-Up Study on Reunion Island. *PLoS ONE [Electronic Resource]* 2016; **11**(1): e0146095.
95. Filio FAR, Aguilar MAQ. Nutritional status of children under five years old beneficiaries of the food support program in the state of Chiapas, Mexico. [Spanish]. *Revista Espanola de Nutricion Comunitaria* 2023; **29**(2).
96. Finkelstein JL, Mehta S, Villalpando S, et al. A randomized feeding trial of iron-biofortified beans in school children in Mexico. *Nutrients* 2019; **11(2) (no pagination)**.
97. Frith AL, Naved RT, Persson LA, Frongillo EA. Early prenatal food supplementation ameliorates the negative association of maternal stress with birth size in a randomised trial. *Maternal and Child Nutrition* 2015; **11**(4): 537-49.
98. Galasso E, Weber AM, Stewart CP, Ratsifandriharnanana L, Fernald LCH. Effects of nutritional supplementation and home visiting on growth and development in young children in Madagascar: a cluster-randomised controlled trial. *Lancet Global Health* 2019; **7**(9): E1257-E68.
99. Ganmaa D, Bromage S, Khudyakov P, Erdenenbaatar S, Delgererekh B, Martineau AR. Influence of vitamin D supplementation on growth, body composition, and pubertal development among school-aged children in an area with a high prevalence of vitamin D deficiency: a randomized clinical trial. *Jama, Pediatr* 2023; **177**(1): 32-41.
100. Gibson RS, Yeudall F, Drost N, Mtitimuni BM, Cullinan TR. Experiences of a community-based dietary intervention to enhance micronutrient adequacy of diets low in animal source foods and high in phytate: a case study in rural Malawian children. *Journal of Nutrition* 2003; **133**(11 Suppl 2): 3992S-9S.
101. Gitau R, Makasa M, Kasonka L, et al. Maternal micronutrient status and decreased growth of Zambian infants born during and after the maize price increases resulting from the southern African drought of 2001-2002. *Public Health Nutr* 2005; **8**(7): 837-43.
102. Goyena EA, Barba CVC, Talavera MTM, Paunlagui MM, Rola AC, Tandang NA. Effects of micronutrient powder and complementary food blend on growth and micronutrient status of Filipino rural children: a randomised controlled trial. *Malaysian Journal of Nutrition* 2018; **24**(4): 475-92.
103. Hall A, Hanh TT, Farley K, Quynh TP, Valdivia F. An evaluation of the impact of a school nutrition programme in Vietnam. *Public Health Nutr* 2007; **10**(8): 819-26.
104. Harris S, Jack S. Home-based treatment of acute malnutrition in Cambodian urban poor communities. *Food Nutr Bull* 2011; **32**(4): 333-9.
105. Heo J, Krishna A, Perkins JM, et al. Community Determinants of Physical Growth and Cognitive Development among Indian Children in Early Childhood: A Multivariate Multilevel Analysis. *Int J Environ Res Public Health* 2020; **17**(1): 12.
106. Hettiarachchi M, Liyanage C, Wickremasinghe R, Hilmers DC, Abrams SA. The efficacy of micronutrient supplementation in reducing the prevalence of anaemia and deficiencies of zinc and iron among adolescents in Sri Lanka. *Eur J Clin Nutr* 2008; **62(7)**: 856-65.
107. Huda TM, Alam A, Tahsina T, et al. Shonjibon cash and counselling: a community-based cluster randomised controlled trial to measure the effectiveness of unconditional cash transfers and mobile behaviour change communications to reduce child undernutrition in rural Bangladesh. *BMC Public Health* 2020; **20**(1): 1776.
108. Hung LQ, de Vries PJ, Giao PT, et al. Nutritional status following malaria control in a Vietnamese ethnic minority commune. *Eur J Clin Nutr* 2005; **59**(8): 891-9.

109. Hurley KM, Phuka J, Kang Y, et al. A longitudinal impact evaluation of a comprehensive nutrition program for reducing stunting among children aged 6-23 months in rural Malawi. *Am J Clin Nutr* 2021; **114**(1): 248-56.
110. Hyder SMZ, Haseen F, Khan M, et al. A multiple-micronutrient-fortified beverage affects hemoglobin, iron, and vitamin A status and growth in adolescent girls in rural Bangladesh. *Journal of Nutrition* 2007; **137(9)**: 2147-53.
111. Iannotti LL, Henretty NM, Delnatus JR, et al. Ready-to-use supplementary food increases fat mass and BMI in Haitian school-aged children. *J Nutr* 2015; **145**(4): 813-22.
112. Inayati DA, Scherbaum V, Purwestri RC, et al. Improved nutrition knowledge and practice through intensive nutrition education: A study among caregivers of mildly wasted children on Nias Island, Indonesia. *Food and Nutrition Bulletin* 2012; **33**(2): 117-27.
113. Kandpal E, Alderman H, Friedman J, Filmer D, Onishi J, Avalos J. A conditional cash transfer program in the Philippines reduces severe stunting. *Journal of Nutrition* 2016; **146**(9): 1793-800.
114. Khan GN, Ariff S, Kureishy S, et al. Effectiveness of wheat soya blend supplementation during pregnancy and lactation on pregnancy outcomes and nutritional status of their infants at 6 months of age in Thatta and Sujawal districts of Sindh, Pakistan: a cluster randomized-controlled trial. *Eur J Nutr* 2021; **60(2)**: 781-9.
115. Kiplagat S, Anisa K, Sheehan DM, et al. Evaluating the moderating role of Accredited Social Health Activists on adverse birth outcomes in rural India. *Sexual and Reproductive Healthcare* 2022; **34**(36).
116. Kowalski AJ, Mayen VA, Ponce Sd, et al. The effects of multiple micronutrient fortified beverage and responsive caregiving interventions on early childhood development, hemoglobin, and ferritin among infants in rural Guatemala. *Nutrients* 2023; **15**(9).
117. Krahenbuhl JD, Schutz Y, Jequier E. High fat versus high carbohydrate nutritional supplementation: a one year trial in stunted rural Gambian children. *European Journal of Clinical Nutrition* 1998; **52**(3): 213-22.
118. Kulwa KBM, Mamiro PS, Kolsteren PW. Nutrition education package focusing on infant and young child feeding in Tanzania. *Journal of Nutrition Education and Behavior* 2023; **55**(7): 493-508.
119. Labib JR, Abd El Fatah SAM, Razik MMA, Shalaby SF. Role of Dietary Habits Modification in Improving Haemoglobin of Anaemic Children in a Rural Village in Egypt. *Journal of Clinical and Diagnostic Research* 2019; **13**(3): SC23-SC8.
120. Labrecque JA, Kaufman JS, Balzer LB, et al. Effect of a conditional cash transfer program on length-for-age and weight-for-age in Brazilian infants at 24 months using doubly-robust, targeted estimation. *Social Science & Medicine* 2018; **211**: 9-15.
121. Langendorf C, Roederer T, de Pee S, et al. Preventing acute malnutrition among young children in crises: a prospective intervention study in Niger. *PLoS Medicine / Public Library of Science* 2014; **11**(9): e1001714.
122. Lechtig A, Cornale G, Ugaz ME, Arias L. Decreasing stunting, anemia, and vitamin A deficiency in Peru: Results of The Good Start in Life Program. *Food Nutr Bull* 2009; **30**(1): 37-48.
123. Lelijveld N, Godbout C, Krietemeyer D, et al. Treating high-risk moderate acute malnutrition using therapeutic food compared with nutrition counseling (Hi-MAM Study): a cluster-randomized controlled trial. *The American journal of clinical nutrition* 2021; **114(3)**: 955-64.
124. Leroy JL, Olney DK, Nduwabike N, Ruel MT. Tubaramure, a Food-Assisted Integrated Health and Nutrition Program, Reduces Child Wasting in Burundi: A Cluster-Randomized Controlled Intervention Trial. *J Nutr* 2021; **151**(1): 197-205.

125. Li J, Huang D, Liang J, et al. Physical activity during pregnancy and adverse birth outcome: a prospective cohort study in China. *J Matern Fetal Neonatal Med* 2023; **36**(1): 2162819.
126. Lind T, Lonnerdal B, Stenlund H, et al. A community-based randomized controlled trial of iron and zinc supplementation in Indonesian infants: effects on growth and development. *American Journal of Clinical Nutrition* 2004; **80**(3): 729-36.
127. Lind T, Seswandhana R, Persson LA, Lonnerdal B. Iron supplementation of iron-replete Indonesian infants is associated with reduced weight-for-age. *Acta Paediatrica* 2008; **97**(6): 770-5.
128. Lisboa CS, Santana JD, Ribeiro-Silva RD, et al. Bolsa Familia Program and Perinatal Outcomes: NISAMI Cohort. *Int J Environ Res Public Health* 2022; **19**(9): 10.
129. Long KZ, Beckmann J, Lang C, et al. Impact of a school-based health intervention program on body composition among South African primary schoolchildren: results from the KaziAfya cluster-randomized controlled trial. *BMC Med* 2022; **20(1) (no pagination)**.
130. Longfils P, Monchy D, Weinheimer H, Chavasit V, Nakanishi Y, Schumann K. A comparative intervention trial on fish sauce fortified with NaFe-EDTA and FeSO4+citrate in iron deficiency anemic school children in Kampot, Cambodia. *Asia Pac J Clin Nutr* 2008; **17(2)**: 250-7.
131. Lucas ADP, Ferreira MD, Lucas TDP, Salari P. The intergenerational relationship between conditional cash transfers and newborn health. *BMC Public Health* 2022; **22**(1): 12.
132. Mahdavi R, Taghipour S, Ostadrahimi A, Nikniaz L, Hezaveh SJG. A pilot study of synbiotic supplementation on breast milk mineral concentrations and growth of exclusively breast fed infants. *J Trace Elem Med Biol* 2015; **30**: 25-9.
133. Mahmudiono T, Al Mamun A, Nindya TS, Andrias DR, Megatsari H, Rosenkranz RR. The effectiveness of nutrition education for overweight/obese mother with stunted children (NEO-MOM) in reducing the double burden of malnutrition. *Nutrients* 2018; **10(12) (no pagination)**.
134. Mamiro PS, Kolsteren PW, van Camp JH, Roberfroid DA, Tatala S, Opsomer AS. Processed complementary food does not improve growth of hemoglobin status of rural Tanzanian infants from 6-12 months of age in Kilosa District, Tanzania. *J Nutr* 2004; **134**(5): 1084-90.
135. Manary MJ, Ndekha MJ, Ashorn P, Maleta K, Briend A. Home based therapy for severe malnutrition with ready-to-use food. *Arch Dis Child* 2004; **89**(6): 557-61.
136. Marquis GS, Colecraft EK, Sakyi-Dawson O, et al. An Integrated Microcredit, Entrepreneurial Training, and Nutrition Education Intervention Is Associated with Better Growth Among Preschool-Aged Children in Rural Ghana. *J Nutr* 2015; **145**(2): 335-43.
137. Marquis GS, Colecraft EK, Kanlisi R, et al. An agriculture-nutrition intervention improved children's diet and growth in a randomized trial in Ghana. (Special Issue: Eggs: a high potential food for improving maternal and child nutrition.). *Maternal and Child Nutrition* 2018; **14**(s3).
138. Maselko J, Sikander S, Bhalotra S, et al. Effect of an early perinatal depression intervention on long-term child development outcomes: follow-up of the Thinking Healthy Programme randomised controlled trial. *Lancet Psychiatry* 2015; **2**(7): 609-17.
139. Matias SL, Vargas-Vasquez A, Perez RB, et al. Effects of lipid-based nutrient supplements v. micronutrient powders on nutritional and developmental outcomes among Peruvian infants. *Public Health Nutr* 2017; **20**(16): 2998-3007.
140. Mayhew M, Ickx P, Stanekzai H, Mashal T, Newbrander W. Improving nutrition in Afghanistan through a community-based growth monitoring and promotion programme: a pre-post evaluation in five districts. *Glob Public Health* 2014; **9 Suppl 1**: S58-75.

141. McLellan KCP, Bianchessi ALV, Rinaldi AE, Michelin E, Burini RC. Improvements of nutrition behavior fitness and body fatness with a short-term after school intervention program. (Special issue on public health nutrition initiatives.). *Food and Nutrition Sciences* 2013; **4**(10A): 18-24.
142. Medeiros DA, Hadler MCCM, Sugai A, Torres VM. The effect of folic acid supplementation with ferrous sulfate on the linear and ponderal growth of children aged 6-24 months: a randomized controlled trial. *European Journal of Clinical Nutrition* 2015; **69**(2): 198-204.
143. Megally R, Ghoneim H. Evaluation of health intervention: a case of preschool children in Egypt. *Palgr Commun* 2020; **6**(1): 8.
144. Mehta K, Mukherjee SG, Bhattacharjee I, et al. Systems strengthening approach during antenatal care improves maternal nutrition and reduces childhood stunting in West Bengal, India. *medRxiv* 2022; **17**.
145. Miller LC, Neupane S, Joshi N, Lohani M. MILK Symposium review: Milk consumption is associated with better height and weight in rural Nepali children over 60 months of age and better head circumference in children 24 to 60 months of age. *Journal of Dairy Science* 2020; **103**(11): 9700-14.
146. Mousa S, Kimiagar SM, Shahbazi M, Mehrabi Y, Kolahi AA. Assessing the impact of nutrition education on growth indices of Iranian nomadic children: an application of a modified beliefs, attitudes, subjective-norms and enabling-factors model. *British Journal of Nutrition* 2004; **91**(5): 779-87.
147. Mridha MK, Matias SL, Chaparro CM, et al. Lipid-based nutrient supplements for pregnant women reduce newborn stunting in a cluster-randomized controlled effectiveness trial in Bangladeshi. *Am J Clin Nutr* 2016; **103**(1): 236-49.
148. Muller O, Garenne M, Reitmaier P, Baltussen van Zweeden A, Kouyate B, Becher H. Effect of zinc supplementation on growth in West African children: A randomized double-blind placebo-controlled trial in rural Burkina Faso. *International Journal of Epidemiology* 2003; **32(6)**: 1098-102.
149. Muthayya S, Thankachan P, Hirve S, et al. Iron Fortification of Whole Wheat Flour Reduces Iron Deficiency and Iron Deficiency Anemia and Increases Body Iron Stores in Indian School-Aged Children¹⁻⁴. *J Nutr* 2012; **142(11)**: 1997-2003.
150. Mwale M, Smith A, von Fintel D. Child nutrition and farm input subsidies: The complementary role of early healthcare and nutrition programs in Malawi. *Food Policy* 2022; **113**: 12.
151. Nabwera HM, Fulford AJ, Moore SE, Prentice AM. Growth faltering in rural Gambian children after four decades of interventions: a retrospective cohort study. *Lancet Glob Health* 2017; **5**(2): e208-e16.
152. Nahar S, Mascie-Taylor CG, Begum HA. Impact of targeted food supplementation on pregnancy weight gain and birth weight in rural Bangladesh: an assessment of the Bangladesh Integrated Nutrition Program (BINP). *Public Health Nutr* 2009; **12**(8): 1205-12.
153. Nesamvuni AE, Vorster HH, Margetts BM, Kruger A. Fortification of maize meal improved the nutritional status of 1-3-year-old African children. (Special issue: Focus on South Africa. To Celebrate the 18th International Congress of Nutrition. September 19-23, 2005. Durban, South Africa.). *Public Health Nutrition* 2005; **8**(5): 461-7.
154. Nikiema L, Huybregts L, Martin-Prevel Y, et al. Effectiveness of facility-based personalized maternal nutrition counseling in improving child growth and morbidity up to 18 months: A cluster-randomized controlled trial in rural Burkina Faso. *PLoS One* 2017; **12**(5): 26.
155. Obatolu V. Impact of soybean utilization project on nutritional status of under five children. *Pakistan Journal of Nutrition* 2006; **5**(4): 348-54.

156. Ojha S, Szatkowski L, Sinha R, et al. Rojiroti microfinance and child nutrition: a cluster randomised trial. *Arch Dis Child* 2020; **105**(3): 229-35.
157. Ouedraogo HZ, Traore T, Zeba A, Dramaix-Wilmet M, Hennart P, Donnen P. A local-ingredient-based, processed flour to improve the energy, iron and zinc intakes of young children: a community-based intervention. *Int J Food Sci Nutr* 2009; **60 Suppl 4**: 87-98.
158. Owais A, Schwartz B, Kleinbaum DG, et al. A Nutrition Education Program in Rural Bangladesh Was Associated with Improved Feeding Practices but Not with Child Growth. *J Nutr* 2017; **147**(5): 948-54.
159. Paes-Sousa R, Santos LMP, Miazaki ES. Effects of a conditional cash transfer programme on child nutrition in Brazil. *Bull World Health Organ* 2011; **89**(7): 496-503.
160. Parakh A, Dubey AP, Gahlot N, Rajeshwari K. Efficacy of modified WHO feeding protocol for management of severe malnutrition in children: a pilot study from a teaching hospital in New Delhi, India. *Asia Pac J Clin Nutr* 2008; **17**(4): 608-11.
161. Penny ME, Creed-Kanashiro HM, Robert RC, Narro MR, Caulfield LE, Black RE. Effectiveness of an educational intervention delivered through the health services to improve nutrition in young children: a cluster-randomised controlled trial. *Lancet* 2005; **365**(9474): 1863-72.
162. Perera UD, Inder BA. Midday meals as an early childhood nutrition intervention: evidence from plantation communities in Sri Lanka. *BMC Public Health* 2021; **21**(1): 22.
163. Phu PV, Hoan NV, Salvignol B, et al. A six-month intervention with two different types of micronutrient-fortified complementary foods had distinct short- and long-term effects on linear and ponderal growth of Vietnamese infants. *Journal of Nutrition* 2012; **142**(9): 1735-40.
164. Phuong Hong N, Kim SS, Sanghvi T, et al. Integrating nutrition interventions into an existing Maternal, Neonatal, and Child Health program increased maternal dietary diversity, micronutrient intake, and exclusive breastfeeding practices in Bangladesh: results of a cluster-randomized program evaluation. *Journal of Nutrition* 2017; **147**(12): 2326-37.
165. Pickering AJ, Djebbari H, Lopez C, Coulibaly M, Alzua ML. Effect of a community-led sanitation intervention on child diarrhoea and child growth in rural Mali: A cluster-randomised controlled trial. *Lancet Glob Health* 2015; **3(11)**: e701-e11.
166. Prawirohartono EP, Nystrom L, Ivarsson A, Stenlund H, Lind T. The impact of prenatal vitamin A and zinc supplementation on growth of children up to 2 years of age in rural Java, Indonesia. *Public Health Nutrition* 2011; **14**(12): 2197-206.
167. Prawirohartono EP, Nystrom L, Nurdiati DS, Hakimi M, Lind T. The impact of prenatal vitamin A and zinc supplementation on birth size and neonatal survival - a double-blind, randomized controlled trial in a rural area of Indonesia. *International Journal for Vitamin and Nutrition Research* 2013; **83**(1): 14-25.
168. Quinones S, Mendola P, Tian L, et al. Ghana's Livelihood Empowerment Against Poverty (1000) Program Seasonally Impacts Birthweight: A Difference-in-Differences Analysis. *International Journal of Public Health* 2023; **68**: 1605336.
169. Rahman A, Abid M, Siham S, Roberts C, Creed F. Cognitive behaviour therapy-based intervention by community health workers for mothers with depression and their infants in rural Pakistan: a cluster-randomised controlled trial. *Lancet* 2008; **372**(9642): 902-9.
170. Rahman A, Bhuiyan MB, Das SK. Effect of short-term educational intervention on complementary feeding index among infants in rural Bangladesh: a randomized control trial. *BMC Nutr* 2022; **8**(1): 10.
171. Ramakrishnan U, Gonzalez-Cossio T, Neufeld LM, Rivera J, Martorell R. Multiple micronutrient supplementation during pregnancy does not lead to greater infant birth size than does iron-only supplementation: a randomized controlled trial in a semirural community in Mexico. *American Journal of Clinical Nutrition* 2003; **77**(3): 720-5.

172. Rasmussen KM, Habicht JP. Maternal supplementation differentially affects the mother and newborn. *J Nutr* 2010; **140**(2): 402-6.
173. Rockers PC, Fink G, Zanolini A, et al. Impact of a community-based package of interventions on child development in Zambia: a cluster-randomised controlled trial. *BMJ Global Health* 2016; **1**(3).
174. Rosado JL, Lopez P, Garcia OP, Alatorre J, Alvarado C. Effectiveness of the nutritional supplement used in the Mexican Oportunidades programme on growth, anaemia, morbidity and cognitive development in children aged 12-24 months. *Public Health Nutrition* 2011; **14**(5): 931-7.
175. Roux IMl, Rotheram-Borus MJ, Stein J, Tomlinson M. The impact of paraprofessional home visitors on infants' growth and health at 18 months. *Vulnerable Children and Youth Studies* 2014; **9**(4): 291-304.
176. Saville NM, Shrestha BP, Style S, et al. Impact on birth weight and child growth of Participatory Learning and Action women's groups with and without transfers of food or cash during pregnancy: Findings of the low birth weight South Asia cluster-randomised controlled trial (LBWSAT) in Nepal. *PLoS One* 2018; **13**(5): 29.
177. Schroeder DG, Pachon H, Dearden KA, et al. An integrated child nutrition intervention improved growth of younger, more malnourished children in northern Vietnam. (The Positive Deviance Approach To Improve Health Outcomes: Experience And Evidence From The Field.). *Food and Nutrition Bulletin* 2002; **23**(4, Supplement): 53-61.
178. Seneviratne SN, Sachchithananthan S, Gamage PSA, Peiris R, Wickramasinghe VP, Somasundaram N. Effectiveness and acceptability of a novel school-based healthy eating program among primary school children in urban Sri Lanka. *BMC Public Health* 2021; **21**(1): 2083.
179. Shaheen R, de Francisco A, El Arifeen S, Ekstrom EC, Persson LA. Effect of prenatal food supplementation on birth weight: an observational study from Bangladesh. *Am J Clin Nutr* 2006; **83**(6): 1355-61.
180. Siddiqua TJ, Roy AK, Akhtar E, et al. Prenatal nutrition supplementation and growth biomarkers in preadolescent Bangladeshi children: A birth cohort study. *Matern Child Nutr* 2022; **18**(1): e13266.
181. Singh V, Ahmed S, Dreyfuss ML, et al. An integrated nutrition and health program package on IYCN improves breastfeeding but not complementary feeding and nutritional status in rural northern India: A quasi-experimental randomized longitudinal study. *PLoS ONE* 2017; **12(9) (no pagination)**.
182. Siswati T, Iskandar S, Pramestuti N, Raharjo J, Rubaya AK, Wiratama BS. Impact of an integrative nutrition package through home visit on maternal and children outcome: finding from locus stunting in Yogyakarta, Indonesia. *Nutrients* 2022; **14**(16).
183. Smuts CM, Matsungo TM, Malan L, et al. Effect of small-quantity lipid-based nutrient supplements on growth, psychomotor development, iron status, and morbidity among 6- to 12-mo-old infants in South Africa: a randomized controlled trial. *American Journal of Clinical Nutrition* 2019; **109**(1): 55-68.
184. Som SV, van der Hoeven M, Laillou A, et al. Adherence to Child Feeding Practices and Child Growth: A Retrospective Cohort Analysis in Cambodia. *Nutrients* 2021; **13**(1): 16.
185. Somasse YE, Dramaix M, Traore B, et al. The WHO recommendation of home fortification of foods with multiple-micronutrient powders in children under 2 years of age and its effectiveness on anaemia and weight: a pragmatic cluster-randomized controlled trial. *Public Health Nutr* 2018; **21(7)**: 1350-8.
186. Soni A, Fahey N, Bhutta Z, et al. Association of trends in child undernutrition and implementation of the National Rural Health Mission in India: A nationally representative serial cross-sectional study on data from 1992 to 2015. *PLoS Med* 2022; **19**(4): 14.

187. Sreeparna Ghosh M, Nagma Nigar S, Pia S. Ensuring pregnancy weight gain: an integrated community-based approach to tackle maternal nutrition in India. *Field Exchange Emergency Nutrition Network ENN* 2019; **61**: 14-7.
188. Stewart CP, Christian P, LeClerq SC, West Jr KP, Khatry SK. Antenatal supplementation with folic acid + iron + zinc improves linear growth and reduces peripheral adiposity in school-age children in rural Nepal. *American Journal of Clinical Nutrition* 2009; **90(1)**: 132-40.
189. Su Y, Heitner J, Si Y, Wang D, Zhou Z, Yuan C. The effects on inappropriate weight for gestational age of an SMS based educational intervention for pregnant women in Xi'an China: A quasi- randomized controlled trial. *International Journal of Environmental Research and Public Health* 2020; **17(5) (no pagination)**.
190. Sunawang, Utomo B, Hidayat A, Kusharisupeni, Subarkah. Preventing low birthweight through maternal multiple micronutrient supplementation: a cluster-randomized, controlled trial in Indramayu, West Java. (Special Issue: Multiple micronutrient supplementation during pregnancy in developing country settings.). *Food and Nutrition Bulletin* 2009; **30**(4 (Supplement): S488-S95.
191. Suprapto B, Dewi YLR. Long-term effect of iodized water and iodized oil supplementation on total goitre rate and nutritional status of school children in Ngargoyoso sub-district, Karanganyar regency, Central Java, Indonesia. *Journal of Biology, Agriculture and Healthcare* 2012; **2**(10): 128-35.
192. Susiloretni KA, Subandriani DN, Ulfiana E, Sunarto, Astuti T, Smith ER. Low-cost local food supplements could improve maternal and birth outcomes in Indonesia: a pilot randomised controlled trial. *Nutrition Bulletin* 2021; **46**(3): 321-31.
193. Tamana SK, Gombojav E, Kanlic A, et al. Portable HEPA filter air cleaner use during pregnancy and children's body mass index at two years of age: The UGAAR randomized controlled trial. *Environ Int* 2021; **156 (no pagination)**.
194. Taneja S, Upadhyay RP, Chowdhury R, et al. Impact of supplementation with milk-cereal mix during 6-12 months of age on growth at 12 months: a 3-arm randomized controlled trial in Delhi, India. *The American journal of clinical nutrition* 2022; **115(1)**: 83-93.
195. Teo CH, Chin YS, Lim PY, Masrom SAH, Shariff ZM. Impacts of a school-based intervention that incorporates nutrition education and a supportive healthy school canteen environment among primary school children in malaysia. *Nutrients* 2021; **13(5) (no pagination)**.
196. Thakur SK, Roy SK, Paul K, Khanam M, Khatun W, Sarker D. Effect of nutrition education on exclusive breastfeeding for nutritional outcome of low birth weight babies. *European Journal of Clinical Nutrition* 2011; **16**.
197. Thakur SK, Roy SK, Paul K, Khanam M, Khatun W, Sarker D. Effect of nutrition education on exclusive breastfeeding for nutritional outcome of low birth weight babies. *Eur J Clin Nutr* 2012; **66**(3): 376-81.
198. Tomedi A, Rohan-Minjares F, McCalmont K, Ashton R, Opiyo R, Mwanthi M. Feasibility and effectiveness of supplementation with locally available foods in prevention of child malnutrition in Kenya. *Public Health Nutrition* 2012; **15**(4): 749-56.
199. Tran Thuy N, Winichagoon P, Dijkhuizen MA, et al. Multi-micronutrient-fortified biscuits decreased prevalence of anemia and improved micronutrient status and effectiveness of deworming in rural Vietnamese school children. *Journal of Nutrition* 2009; **139**(5): 1013-21.
200. Vellakkal S, Fledderjohann J, Basu S, et al. Food Price Spikes Are Associated with Increased Malnutrition among Children in Andhra Pradesh, India. *Journal of Nutrition* 2015; **145**(8): 1942-9.

201. Wang W, Yan H, Zeng L, Cheng Y, Wang D, Li Q. No effect of maternal micronutrient supplementation on early childhood growth in rural western China: 30 month follow-up evaluation of a double blind, cluster randomized controlled trial. *European Journal of Clinical Nutrition* 2012; **66**(2): 261-8.
202. Wang J, Hernandez MA, Deng G. Large-scale school meal programs and student health evidence from rural China. *IFPRI Discussion Papers* 2021; **40**.
203. Ward A, Guillot A, Nepomnyashchiy LE, et al. Seasonal malaria chemoprevention packaged with malnutrition prevention in northern Nigeria: A pragmatic trial (SMAMP study) with nested case-control. *PLoS ONE [Electronic Resource]* 2019; **14**(1): e0210692.
204. Xie H, Qiao L, Zhao Y, et al. Nutrition education with or without oral nutrition supplements has contrasting effects on nutrition status in older adults: a randomized controlled study. (Special Issue: Key events affecting nutrition support.). *Nutrition in Clinical Practice* 2023; **38**(1): 138-47.
205. Xu S, Zhang J, Dong Y, et al. The PROMOTE study (High-protein and resistance-training combination in overweight and obesity) for short-term weight loss and long-term weight maintenance for Chinese people: a protocol for a pilot randomized controlled trial. *Trials [Electronic Resource]* 2020; **21**(1): 47.
206. Yeudall F, Gibson RS, Kayira C, Umar E. Efficacy of a multi-micronutrient dietary intervention based on haemoglobin, hair zinc concentrations, and selected functional outcomes in rural Malawian children. *Eur J Clin Nutr* 2002; **56**(12): 1176-85.
207. Yue A, Cui ML, Yang ZL, Shi YJ, Guo C, Song QN. Formula feeding: Evidence for health, nutrition and early childhood development during the critical first 1000 days from rural China. *J Asian Econ* 2023; **85**: 17.
208. Zhang X, Chen K, Qu P, Liu Y, Li T. Effect of biscuits fortified with different doses of vitamin A on indices of vitamin A status, haemoglobin and physical growth levels of pre-school children in Chongqing. *Public Health Nutrition* 2010; **13**(9): 1462-71.
209. Anindo M, Arun M, Parthibane S, Revadi G. Young, obese, and underweight patients show up inadequately at scheduled appointments: findings from a record-based study on diabetic, hypertensive diabetic, and hypertensive patients attending a primary care clinic of Puducherry. *Journal of Family Medicine and Primary Care* 2019; **8**(3): 1090-7.
210. Ballout G, Al-Shorbaji N, Zeidan W, et al. The impact of e-health system implementation on UNWRA health services: an observational study. *Lancet (London, England)* 2021; **398(Supplement 1)**: S17.
211. Dabone C, Delisle HF, Receveur O. Poor nutritional status of schoolchildren in urban and peri-urban areas of Ouagadougou (Burkina Faso). *Nutr J* 2011: 34.
212. das Chagas DC, Silva AAM, Batista RFL, et al. Prevalence and factors associated to malnutrition and excess weight among under five year-olds in the six largest cities of Maranhao. *Rev* 2013; **16(1)**: 146-56.
213. Egbi G, Glover-Amengor M, Tohouenou MM, Zotor F. Contribution of Amaranthus cruentus and Solanum macrocarpon Leaves Flour to Nutrient Intake and Effect on Nutritional Status of Rural School Children in Volta Region, Ghana. *J Nutr Metab* 2020; **2020 (no pagination)**.
214. Fall CHD, Fisher DJ, Osmond C, Margetts BM. Multiple micronutrient supplementation during pregnancy in low-income countries: a meta-analysis of effects on birth size and length of gestation. (Special Issue: Multiple micronutrient supplementation during pregnancy in developing country settings.). *Food and Nutrition Bulletin* 2009; **30**(4 (Supplement): S533-S46.
215. Ford ND, Behrman JR, Hoddinott JF, et al. Exposure to improved nutrition from conception to age 2 years and adult cardiometabolic disease risk: a modelling study. *Lancet Glob Health* 2018; **6**(8): e875-e84.

216. Garcia-Guerra A, Neufeld LM, Arenas AB, et al. Closing the Nutrition Impact Gap Using Program Impact Pathway Analyses to Inform the Need for Program Modifications in Mexico's Conditional Cash Transfer Program. *Journal of Nutrition* 2019; **149**(12): 2281S-9S.
217. Gurri FD. The Disruption of Subsistence Agricultural Systems in Rural Yucatan, Mexico may have Contributed to the Coexistence of Stunting in Children with Adult Overweight and Obesity. *Coll Antropol* 2015; **39**(4): 847-54.
218. Kusum B, Manoj K, Pramila P. Impact of socio-economic status (SES) and nutrition health education on nutritional status of adolescent girls. *Food Science Research Journal* 2020; **11**(2): 134-9.
219. Ravi Y, Usha R. Nutri-farms for nutritional security of farm women: a study in Chamarajanagara district of Karnataka. *Mysore Journal of Agricultural Sciences* 2017; **51**(3): 516-21.
220. Sanchez Bernal SF, Aguilar A, Romero L, et al. Nutritional evolution of children under 5 years of age users of an Integrated Nutritional Food Program in Paraguay. *Pediatria* 2017; **44**(1): 15-22.
221. Shaveta M, Rajbir S, Anita K. Clinical and haematological profile of urban working women as influenced by nutritional counselling. *Journal of Human Ecology* 2007; **22**(2): 149-52.
222. Silveira BC, Nolasco SAVN, Lopes VAA, Netto MP, Costa FMd. Impact of food complement supplied by a food bank on the nutritional status of children aged 1 to 6 years in a daycare center in Ibirite/Minas Gerais. *Nutrire Revista da Sociedade Brasileira de Alimentacao e Nutricao* 2011; **36**(1): 23-35.
223. Sperandio N, Rodrigues C, Franceschini S, do C, Priore S. Impact of Bolsa Familia Program on the nutritional status of children and adolescents from two Brazilian regions. *Revista de Nutricao* 2017; **30**(4): 477-87.
224. Vasquez-Garibay EM, Miranda-Rios L, Romero-Velarde E, et al. Stunting, overweight and obesity during the nutrition transition in schoolchildren of Arandas, Jalisco, Mexico. *Rev Med Inst Mex Seguro Soc* 2018; **56**(1): 6-11.
225. Vinod W, Rao BS, Devkinandan R. A study of the health status of early adolescent girls residing in social welfare hostels in Vizianagaram district of Andhra Pradesh State, India. *International Journal of Collaborative Research on Internal Medicine and Public Health* 2012; **4**(1): 72-83.
226. Bhandari N, Mazumder S, Bahl R, Martines J, Black RE, Bhan MK. An educational intervention to promote appropriate complementary feeding practices and physical growth in infants and young children in rural Haryana, India. *J Nutr* 2004; **134**(9): 2342-8.
227. Christian P, Khatry SK, Katz J, et al. Effects of alternative maternal micronutrient supplements on low birth weight in rural Nepal: double blind randomised community trial. *Bmj* 2003; **326**(7389): 571.
228. Cogswell ME, Parvanta I, Ickes L, Yip R, Brittenham GM. Iron supplementation during pregnancy, anemia, and birth weight: a randomized controlled trial. *Am J Clin Nutr* 2003; **78**(4): 773-81.
229. Cook A, Chambel A, McWhinney D, Ashraf S, Senyonga R. Strategic Evaluation of WFP’s work on Nutrition and HIV/AIDS. Rome, Italy: World Food Programme, 2023.
230. Cruz S. Rwanda, Home-Grown School Feeding (2020-2025): Evaluation. Rome, Italy: World Food Programme, 2022.
231. Draper CE, de Villiers A, Lambert EV, et al. HealthKick: a nutrition and physical activity intervention for primary schools in low-income settings. *BMC Public Health* 2010; **10**: 398.
232. Cruz ADL. EVALUACIÓN CONJUNTA DE LA ACTIVIDAD ARTICULADA DE PROGRESANDO CON SOLIDARIDAD Y EL SERVICIO NACIONAL DE SALUD, CON

APOYO DEL PROGRAMA MUNDIAL DE ALIMENTOS, PARA LA PREVENCIÓN DE LA DESNUTRICIÓN Y LA ANEMIA EN POBLACIÓN NUTRICIONALMENTE VULNERABLE DE LA REPÚBLICA DOMINICANA 2014 - 2020. Rome, Italy: World Food Programme, 2021.
233. Friis H, Gomo E, Nyazema N, et al. Effect of multimicronutrient supplementation on gestational length and birth size: a randomized, placebo-controlled, double-blind effectiveness trial in Zimbabwe. *Am J Clin Nutr* 2004; **80**(1): 178-84.
234. Gupta P, Ray M, Dua T, Radhakrishnan G, Kumar R, Sachdev HP. Multimicronutrient supplementation for undernourished pregnant women and the birth size of their offspring: a double-blind, randomized, placebo-controlled trial. *Arch Pediatr Adolesc Med* 2007; **161**(1): 58-64.
235. Joshi S, Schultz TP. Family planning and women's and children's health: long-term consequences of an outreach program in Matlab, Bangladesh. *Demography* 2013; **50**(1): 149-80.
236. Kaul T, Husain S, Tyrrell T, Gaarder M, Jimenez E. Four Evaluations of the Impact of WFP Programmes on Nutrition in Humanitarian Contexts in the Sahel: A Synthesis. Rome, Italy: World Food programme, 2018.
237. Kouam CE, Roberts C, Henrique JM. End-Term Evaluation of treatment of Moderate Acute Malnutrition in Timor-Leste. Timor-Leste: World Food Programme, 2018.
238. Osrin D, Vaidya A, Shrestha Y, et al. Effects of antenatal multiple micronutrient supplementation on birthweight and gestational duration in Nepal: double-blind, randomised controlled trial. *Lancet* 2005; **365**(9463): 955-62.
239. Luzot A-C. Kenya, Cash-Based Transfers and Gender: Impact Evaluation. Rome, Italy: World Food Programme - Office of Evaluation, 2023.
240. Penny ME, Creed-Kanashiro HM, Robert RC, Narro MR, Caulfield LE, Black RE. Effectiveness of an educational intervention delivered through the health services to improve nutrition in young children: a cluster-randomised controlled trial. *Lancet* 2005; **365**(9474): 1863-72.
241. Ramakrishnan U, González-Cossío T, Neufeld LM, Rivera J, Martorell R. Multiple micronutrient supplementation during pregnancy does not lead to greater infant birth size than does iron-only supplementation: a randomized controlled trial in a semirural community in Mexico. *Am J Clin Nutr* 2003; **77**(3): 720-5.
242. Rivera JA, Habicht JP. Effect of supplementary feeding on the prevention of mild-to-moderate wasting in conditions of endemic malnutrition in Guatemala. *Bulletin of the World Health Organization* 2002; **80**(12): 926-32.
243. Robertson L, Mushati P, Eaton JW, et al. Effects of unconditional and conditional cash transfers on child health and development in Zimbabwe: a cluster-randomised trial. *Lancet* 2013; **381**(9874): 1283-92.
244. Taylor RW, McAuley KA, Barbezat W, Strong A, Williams SM, Mann JI. APPLE Project: 2-y findings of a community-based obesity prevention program in primary school age children. *Am J Clin Nutr* 2007; **86**(3): 735-42.
245. Vaidya A, Saville N, Shrestha BP, Costello AM, Manandhar DS, Osrin D. Effects of antenatal multiple micronutrient supplementation on children's weight and size at 2 years of age in Nepal: follow-up of a double-blind randomised controlled trial. *Lancet* 2008; **371**(9611): 492-9.
246. Zagré NM, Desplats G, Adou P, Mamadoultaibou A, Aguayo VM. Prenatal multiple micronutrient supplementation has greater impact on birthweight than supplementation with iron and folic acid: a cluster-randomized, double-blind, controlled programmatic study in rural Niger. *Food Nutr Bull* 2007; **28**(3): 317-27.

247. Black RE, Victora CG, Walker SP, et al. Maternal and child undernutrition and overweight in low-income and middle-income countries. *Lancet* 2013; **382**(9890): 427-51.
248. Roberfroid D, Huybregts L, Henry MC, et al. Effects of maternal multiple micronutrient supplementation on fetal growth: A double-blind randomized controlled trial in rural Burkina Faso. *Am J Clin Nutr* 2008; **88(5)**: 1330-40.
249. Sahariah SA, Gandhi M, Chopra H, et al. Body Composition and Cardiometabolic Risk Markers in Children of Women who Took Part in a Randomized Controlled Trial of a Pre-conceptional Nutritional Intervention in Mumbai, India. *The Journal of nutrition* 2022; **07**.
250. Iversen PO, Ngari M, Westerberg AC, Muhoozi G, Atukunda P. Child stunting concurrent with wasting or being overweight: A 6-y follow up of a randomized maternal education trial in Uganda. *Nutrition* 2021; **89**: 111281.
251. Susanto T, Yunanto RA, Rasny H, Susumaningrum LA, Nur KRM. Promoting Children Growth and Development: A community-based cluster randomized controlled trial in rural areas of Indonesia. *Public Health Nursing* 2019; **36**(4): 514-24.
252. Navarro JI, Sigulem DM, Ferraro AA, Polanco JJ, Barros AJ. The double task of preventing malnutrition and overweight: a quasi-experimental community-based trial. *BMC Public Health* 2013; **13**: 212.
253. Martinez S, Johannsen J, Gertner G, et al. Effects of a home-based participatory play intervention on infant and young child nutrition: a randomised evaluation among low-income households in El Alto, Bolivia. *BMJ glob* 2018; **3**(3).
254. Lutter CK, Rodriguez A, Fuenmayor G, Avila L, Sempertegui F, Escobar J. Growth and micronutrient status in children receiving a fortified complementary food. *Journal of Nutrition* 2008; **138**(2): 379-88.
255. Leroy JL, Olney DK, Ruel MT. PROCOMIDA, a Food-Assisted Maternal and Child Health and Nutrition Program, Contributes to Postpartum Weight Retention in Guatemala: A Cluster-Randomized Controlled Intervention Trial. *Journal of Nutrition* 2019; **149**(12): 2219-27.
256. Christian P, Hurley KM, Phuka J, et al. Impact Evaluation of a Comprehensive Nutrition Program for Reducing Stunting in Children Aged 6-23 Months in Rural Malawi. *J Nutr* 2020; **150**(11): 3024-32.
257. Jamaluddine Z, Choufani J, Masterson AR, Hoteit R, Sahyoun NR, Ghattas H. A Community-Based School Nutrition Intervention Improves Diet Diversity and School Attendance in Palestinian Refugee Schoolchildren in Lebanon. *Curr* 2020; **4**(11).
258. Kremer P, Waqa G, Vanualailai N, et al. Reducing unhealthy weight gain in Fijian adolescents: results of the Healthy Youth Healthy Communities study. *Obes Rev* 2011; **12**: 29-40.
259. Muller I, Schindler C, Adams L, et al. Effect of a multidimensional physical activity intervention on body mass index, skinfolds and fitness in south african children: Results from a cluster-randomised controlled trial. *Int J Environ Res Public Health* 2019; **16**(2).
260. Nqweniso S, Du Randt R, Adams L, et al. Effect of school-based interventions on body composition of grade-4 children from lower socioeconomic communities in Gqeberha, South Africa. *SAJCH South African Journal of Child Health* 2021; **15**(2): 89-98.
261. Edde CE, Delisle H, Dabone C, Batal M. Impact of the Nutrition-Friendly School Initiative: analysis of anthropometric and biochemical data among school-aged children in Ouagadougou. *Glob Health Promot* 2020; **27**(2): 26-34.
262. Bhave S, Pandit A, Yeravdekar R, et al. Effectiveness of a 5-year school-based intervention programme to reduce adiposity and improve fitness and lifestyle in Indian children; the SYM-KEM study. *Arch Dis Child* 2016; **101**(1): 33-41.

263. Fernald LC, Gertler PJ, Neufeld LM. Role of cash in conditional cash transfer programmes for child health, growth, and development: an analysis of Mexico's Oportunidades. *The Lancet* 2008; **371(9615)**: 828-37.
264. Lopez-Arana S, Avendano M, Forde I, Lenthe FJv, Burdorf A. Conditional cash transfers and the double burden of malnutrition among children in Colombia: a quasi-experimental study. *Br J Nutr* 2016; **115**(10): 1780-9.
265. Andersen CT, Reynolds SA, Behrman JR, et al. Participation in the Juntos Conditional Cash Transfer Program in Peru Is Associated with Changes in Child Anthropometric Status but Not Language Development or School Achievement. *J Nutr* 2015; **145**(10): 2396-405.
266. Perez-Lu JE, Carcamo C, Nandi A, Kaufman JS. Health effects of 'Juntos', a conditional cash transfer programme in Peru. *Matern Child Nutr* 2017; **13**(3).
267. Dursun B, Cesur R, Mocan N. The Impact of Education on Health Outcomes and Behaviors in a Middle-Income, Low-Education Country. *Economics and Human Biology* 2018; **31**: 94-114.
268. Barham T, Champion B, Foster AD, et al. Thirty-five years later: Long-term effects of the Matlab maternal and child health/family planning program on older women's well-being. *Proc Natl Acad Sci U S A* 2021; **118**(28): 13.
269. Kumordzie SM, Adu-Afarwuah S, Arimond M, et al. Maternal and Infant Lipid-Based Nutritional Supplementation Increases Height of Ghanaian Children at 4-6 Years Only if the Mother Was Not Overweight Before Conception. *J Nutr* 2019; **149**(5): 847-55.
270. Benitez-Guerrero V, Vazquez-Arambula IdJ, Sanchez-Gutierrez R, Velasco-Rodriguez R, Ruiz-Bernes S, Medina-Sanchez MdJ. Educational intervention on nutritional status and knowledge on diet and physical activity in school children. *Revista de Enfermeria del Instituto Mexicano des Seguro Social* 2016; **24**(1): 37-43.
271. Wang D, Natchu UCM, Darling AM, et al. Effects of prenatal and postnatal maternal multiple micronutrient supplementation on child growth and morbidity in Tanzania: a double-blind, randomized-controlled trial. *International Journal of Epidemiology* 2022; **51(6)**: 1761-74.
272. Kok Bd, Toe LC, Hanley-Cook G, et al. Prenatal fortified balanced energy-protein supplementation and birth outcomes in rural Burkina Faso: a randomized controlled efficacy trial. *PLoS Medicine* 2022; **19**(5).
273. Vitolo MR, Bueno MSF, Gama CM. Impact of a dietary counseling program on the gain weight speed of pregnant women attended in a primary care service. *Revista Brasileira de Ginecologia e Obstetricia* 2011; **33**(1): 13-9.
274. BMJ Best Practice - Study design search filters. https://bestpractice.bmj.com/info/toolkit/learn-ebm/study-design-search-filters/ (accessed February 22, 2022.
275. Glanville J, Eyers J, Jones AM, et al. Quasi-experimental study designs series-paper 8: identifying quasi-experimental studies to inform systematic reviews. *J Clin Epidemiol* 2017; **89**: 67-76.
276. Quality assessment tool for quantitative studies https://www.ephpp.ca/quality-assessment-tool-for-quantitative-studies/ (accessed February 22, 2022.
